# Dog-assisted interventions for children and adults with mental health or neurodevelopmental conditions: systematic review

**DOI:** 10.1192/bjp.2025.8

**Published:** 2026-02

**Authors:** Emily Shoesmith, Sophie Hall, Amanda Sowden, Heidi Stevens, Jodi Pervin, Jenny Riga, Dean McMillan, Daniel Mills, Chris Clarke, Qi Wu, Selina Gibsone, Elena Ratschen

**Affiliations:** Department of Health Sciences, University of York, UK; Nottingham Clinical Trials Unit, University of Nottingham, UK; Centre for Reviews and Dissemination, University of York, UK; Department of Life Sciences, University of Lincoln, UK; Foss Park Hospital, Tees, Esk and Wear Valleys NHS Foundation Trust, UK; Dogs for Good, The Frances Hay Centre, Banbury, UK

**Keywords:** Canine-assisted interventions, dog-assisted interventions, mental health conditions, neurodevelopmental conditions, systematic review

## Abstract

**Background:**

Dog-assisted interventions (DAIs) to improve health-related outcomes for people with mental health or neurodevelopmental conditions are becoming increasingly popular. However, DAIs are not based on robust scientific evidence.

**Aims:**

To determine the effectiveness of DAIs for children and adults with mental health or neurodevelopmental conditions, assess how well randomised controlled trials (RCTs) are reported, and examine the use of terminology to classify DAIs.

**Methods:**

A systematic search was conducted in Embase, PsycINFO, PubMed, CINAHL, Web of Science and the Cochrane Library. RCTs were grouped by commonly reported outcomes and described narratively with forest plots reporting standardised mean differences and 95% confidence intervals without a pooled estimate. The quality of reporting of RCTs and DAIs was evaluated by assessing adherence to CONSORT and the Template for Intervention Description and Replication (TIDieR) guidelines. Suitability of use of terminology was assessed by mapping terms to the intervention content described.

**Results:**

Thirty-three papers were included, reporting 29 RCTs (with five assessed as overall high quality); a positive impact of DAIs was found by 57% (8/14) for social skills and/or behaviour, 50% (5/10) for symptom frequency and/or severity, 43% (6/14) for depression and 33% (2/6) for agitation. The mean proportion of adherence to the CONSORT statement was 48.6%. The TIDieR checklist also indicated considerable variability in intervention reporting. Most DAIs were assessed as having clear alignment for terminology, but improvement in reporting information is still required.

**Conclusions:**

DAIs may show promise for improving mental health and behavioural outcomes for those with mental health or neurodevelopmental conditions, particularly for conditions requiring social skill support. However, the quality of reporting requires improvement.

Mental health conditions constitute a leading cause of disability worldwide.^1^ The World Health Organization^2^ defines the term ‘mental disorders’ as describing a range of mental and behavioural conditions that fall within the ICD-11.^3^ These include disorders that cause a high burden of disease such as depression, bipolar affective disorder, schizophrenia, anxiety disorders, dementia, intellectual disabilities, and developmental and behavioural disorders with onset usually occurring in childhood and adolescence (e.g. autism spectrum condition (ASC)).^2,3^ The need to develop and test new interventions to improve outcomes and quality of life related to these conditions is widely acknowledged.^4–7^

## Animal-assisted interventions for mental health and neurodevelopmental conditions

Animal-assisted interventions (AAIs) have been receiving increasing interest as (complementary) interventions to improve health-related outcomes, especially those focused on mental health, across various age groups.^8–11^ In a health-focused context, AAIs intentionally include animals in health, education and social services contexts for therapeutic or other ameliorative purposes. Health-focused AAIs include animal-assisted therapy, which is goal-orientated, structured, documented and delivered by trained professionals; and animal-assisted activities, which are also goal-orientated but typically based on spontaneous interaction and delivered usually by volunteers and non-specialist trained animals. Although a variety of species (e.g. dogs, horses, small mammals, farm animals) can be involved in AAIs in research and practice, dog-assisted interventions (DAIs) are the most commonly provided and researched type of AAI.^12^

Research suggests that DAIs might improve a range of mental health and behavioural outcomes such as anxiety, agitation, and feelings of depression and loneliness, while enhancing positive social interaction.^13–16^ Although overall poorly understood, mechanisms underlying these effects have been hypothesised to be related to, for example, the calming and motivating effects of the dog’s presence, which in turn might catalyse participants’ engagement with therapy.^17^ Recently, there has been much enthusiasm for and a rapid increase in the provision of DAIs for a wide range of mental health and neurodevelopmental conditions in practice,^14,15,18–21^ with DAIs being increasingly offered by third-sector organisations or by teams affiliated with health and social care or educational settings.

## Current limitations in animal-assisted intervention research

However, DAIs are currently not based on robust evidence. Although findings from generally small randomised controlled trials (RCTs) have been reported,^15,22–24^ evidence synthesis has unanimously highlighted common methodological problems and a lack of rigour in study design.^8,25–27^ Key issues include small sample sizes and consequently a lack of statistical power, as well as an absence of manualised intervention protocols and well-designed control conditions.^8,28,29^ Design issues are further compounded by limited intervention reporting, restricting the opportunity for reproducibility and comparability.^28,30^ The complex nature of DAIs in health-related contexts, involving inter-species interactions between several actors including a dog and a vulnerable patient, also requires consideration of welfare and safety for the participants, dog and handler that exceeds current design and reporting practice in the field.^29,31^ Notably, common terminological and conceptual confusion with regard to the definition of DAIs and their application in practice and research contexts has been identified, further compounding transparency.^32^

Several evidence syntheses have been conducted to explore the impact of DAIs in populations with mental health and neurodevelopmental conditions,^25,29,33,34^ with wide variation in review focus (e.g. on specific diagnostic groups, settings or age groups), methodological quality and terminology used. No existing systematic review has formally evaluated the reporting quality of RCTs delivering DAIs by assessing adherence to gold standard reporting guidelines such as CONSORT^35^ or evaluated the quality and completeness of reporting DAIs, for example, by assessing intervention reporting in accordance with the Template for Intervention Description and Replication (TIDieR) guide.^36^ Likewise, no existing systematic review has examined how these interventions are described, practised and reported. Thus, the research aims for this review were:

- to examine the use of terminology and definitions chosen to classify DAIs in the included RCTs;
- to determine the effectiveness of mental-health-focused DAIs for populations with mental health and neurodevelopmental conditions in clinical and community (including educational) settings;
- to assess how well RCTs delivering DAIs to people with mental health and neurodevelopmental conditions are reported based on internationally recognised gold standard reporting guidelines (CONSORT and TIDieR).

## Methods

We report methodology in accordance with the Preferred Reporting Items for Systematic Reviews and Meta-analyses guidelines,^37^ following a preregistered International Prospective Register of Systematic Reviews protocol (CRD42024526375). An amendment to the protocol was made to add the first review question. We believed this was an important addition owing to the ambiguity and inconsistent terminology for DAIs used across this research area.

### Inclusion criteria

Studies were assessed for inclusion based on the population, intervention, comparator, outcome and study design (Table 1).^38^

**Table 1:** Inclusion criteria based on population, intervention, comparator, outcome and study design

Population	Studies that included children (aged up to 18 years) and/or adults (aged 18 years and above) with a diagnosis of a mental health or neurodevelopmental condition (as defined by the ICD-11^3^), in clinical and community (including educational) settings. Studies evaluating dog-assisted interventions (DAIs) delivered to participants with dementia were included, as a diagnosis of dementia is categorised among ‘mental, behavioural or neurodevelopmental disorders’ in the ICD-11^3^
Intervention	DAIs (including dog-assisted therapy and dog-assisted activity) delivered to participants with a diagnosis of a mental health and/or neurodevelopmental condition
Comparator	Studies with the following controls were considered: normal practice (‘usual care’), waiting-list control or any other intervention described by the authors as a comparator
Outcomes	Studies that reported: mental health and behavioural outcomes (e.g. agitation, anxiety, social behaviour, verbalisation)
Study designs	Randomised controlled trials (including randomised feasibility and pilot trials)

### Exclusion criteria

Studies were excluded if: (a) they described or evaluated the impact of living with pet dogs or assistance dogs; (b) the DAIs were primarily education interventions with educational outcomes (e.g. reading), as DAIs were only included if they were delivered for health-related and/or therapeutic purposes; (c) interventions involved species other than dogs; (d) interventions involved robotic dogs; (e) they did not assess the impact on outcomes for people with a mental health or neurodevelopmental condition; or (f) they were systematic reviews, theses, dissertations or not original research.

### Search strategy

**Fig. 1:**
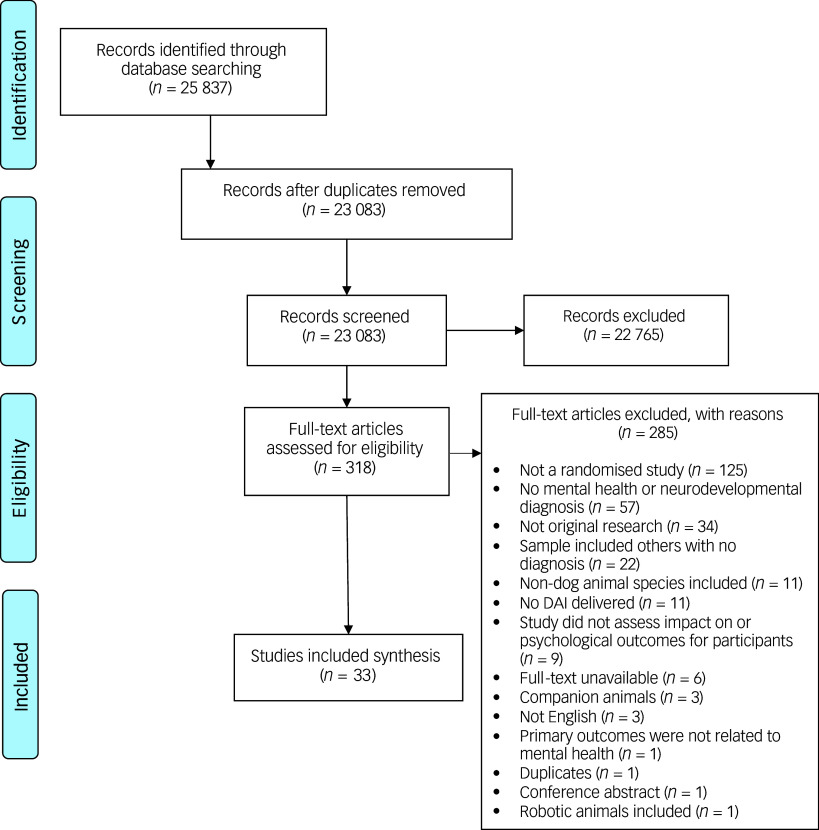
PRISMA diagram of paper selection process. DAI, dog-assisted intervention.

Embase, PsycINFO, PubMed, CINAHL, Web of Science and the Cochrane Library were searched up to 30 April 2024. A comprehensive search strategy was developed using subject headings and words related to DAIs (e.g. dog-assisted therapy, dog-assisted activities, dog-assisted interventions, animal-assisted interventions, therapy dogs, therapy animals) and mental health or neurodevelopmental conditions in children and adult populations. Searches were limited to studies published in English. The search strategy for Embase is provided in Supplementary Material 1 available at https://doi.org/10.1192/bjp.2025.8 and was adapted for the other included databases. Reference lists of included papers and systematic reviews of DAIs for mental health and neurodevelopmental conditions were manually screened to identify potential further studies. Covidence was used to record publications at all stages of the selection process (Fig. 1). Titles and abstracts were screened independently by two authors (E.S. and J.P.). If there was a disagreement, studies were included in the full-text review. Full-text screening was undertaken independently by two authors (E.S. and J.P.), and any disagreements were resolved with a third author (E.R.).

### Data extraction

Using a predefined data extraction worksheet in Microsoft Excel, relevant data were extracted by one author (E.S.). Information included research methodology; sample size; follow-up periods; type and content of the intervention and control groups; mode of delivery; frequency and duration; participant details including diagnosis; diagnostic criteria; role of animal handlers; aspects related to selection, training and safety of the animals involved; and outcomes of the intervention. A complete list of data extracted is provided in Supplementary Material 2. Data extraction commenced on 12 May 2024.

### Risk of bias assessment

Two authors (E.S. and J.P.) independently assessed the risk of bias of each RCT using the Cochrane risk of bias tool.^39^ Consensus was reached through discussion between the two authors. Data from the risk of bias assessment were entered into Review Manager 5.3^40^ to generate a summary figure. Risk of bias was used for critique of the research evidence and not as an exclusion criterion. The risk of bias for all domains was summarised to produce an overall risk of bias for each RCT. RCTs were classified as having an overall high risk of bias if they scored ‘unclear’ or ‘high’ in any bias domain other than performance bias, as the nature of DAIs made blinding of participants and personnel difficult.

### Data synthesis

As per the protocol, a meta-analysis was planned, given low heterogeneity as assessed using the *I* ^2^ statistic. However, owing to clinical and methodological heterogeneity, we determined that a statistical meta-analysis would have been inappropriate; therefore, a narrative synthesis was performed to summarise the effectiveness of DAIs. Trials were grouped by commonly reported mental health and behavioural outcomes, and findings were described narratively, using forest plots to report standardised mean difference (SMD) plus 95% confidence interval without a pooled estimate. SMD is the mean difference in outcome scores between the intervention and control group divided by the pooled standard deviation at follow-up, resulting in a unit-free effect size. By convention, SMD effect sizes of 0.2, 0.5 and 0.8 are considered to indicate small, medium and large intervention effects, respectively.^41^ The direction of effect was assessed based on the effects reported by authors of the included studies and the forest plots produced. The direction of effect (or lack of a difference between intervention and control) was used to determine the effectiveness of DAIs. Findings are presented for three categories: mental health conditions, neurodevelopmental conditions and dementia. Although a diagnosis of dementia is categorised in ‘mental, behavioural or neurodevelopmental disorders’ in the ICD-11,^3^ studies involving adults with dementia are presented separately to those with other mental health conditions owing to the distinct aetiology of the condition.

The CONSORT statement^42^ was used to assess the quality of reporting of RCTs. Two authors (E.S. and H.S.) individually assessed each paper, and each item was scored ‘yes’ if adequately reported or ‘no’ if inadequately, inconsistently or not at all reported. Reporting of an item in supplementary material was considered to be acceptable only if this was clearly cited in the main text. In addition, the TIDieR checklist^36^ was used for appraisal of quality and completeness of reporting intervention details. Data in each of the papers and any supplementary material cited within the papers were used. Two authors (E.S. and J.P.) individually assessed each study, and each item was scored ‘yes’ if adequately reported or ‘no’ if inadequately or inconsistently reported, or not applicable. Cohen’s kappa (κ) was calculated to assess the agreement between reviewers for both appraisals using the CONSORT statement and the TIDieR checklist. Interpretation of the coefficient was as follows: ‘none’ 0–0.20; ‘minimal’ 0.21–0.39; ‘weak’ 0.40–0.59; ‘moderate’ 0.60–0.79’; ‘strong’ 0.80–0.90; and ‘almost perfect’ ≥0.90.^43^ Data were analysed in IBM SPSS version 28.^44^

Terminology used by authors to classify DAIs as ‘dog-assisted therapy’ or ‘dog-assisted activity’ was extracted for each study and assessed for suitability of use by two authors (E.S. and H.S.) based on the intervention content described, using internationally recognised definitions from the International Association of Human-Animal Interaction Organisations^45^ and Animal-Assisted Intervention International.^46^ We did not use the new terminology proposed in early 2024,^32^ as this would not have corresponded to terminology and classifications used in the studies, all of which were conducted before the 2024 recommendations were published. We assessed alignment between study terminology and conceptual definitions using three categories: (a) clear alignment of content and terminology, (b) unclear alignment of content and terminology (e.g. owing to limited information in the manuscript) and (c) misalignment of content and terminology (e.g. a DAI was described as therapy, but the content description clearly depicted activity).

## Results

### Description of studies

Database searches yielded a total of 25 837 records. After removal of duplicates and screening of titles, abstracts and full-text papers, 33 papers were included in the review (Fig. 1), reporting a total of 29 studies. The independent screening of titles and abstracts and full-text papers both yielded a Cohen’s kappa of 0.77. Two papers evaluating a DAI delivered to adults with ASC referred to the same RCT,^21,23^ two papers delivered to adults with schizophrenia referred to the same RCT,^14,47^ and three papers delivered to children with attention-deficit hyperactivity disorder (ADHD) referred to the same RCT.^15,48,49^ All of these papers were included, as they assessed different relevant outcomes. A list of all included papers is provided in Supplementary Material 3. Thirty-three papers described 29 small-scale RCTs (intervention sample size range: 5–186; control sample size range: 4–185). Study follow-up ranged from immediately post-intervention^14,16,18,19,24,47,50–63^ to 3 months.^20,64–67^

DAIs were delivered to a variety of study populations, including individuals with dementia (*n* = 11), schizophrenia (*n* = 5), ASC (*n* = 3), ADHD (*n* = 2), any acute psychiatric diagnosis (*n* = 2), fetal alcohol spectrum disorder (*n* = 2), intellectual disabilities (*n* = 1), anxiety or depression (*n* = 1), post-traumatic stress disorder (*n* = 1) or mixed diagnoses (e.g. ASC, ADHD, intellectual disabilities) (*n* = 1). DAIs were delivered to a variety of age groups, including children (4–12 years; *n* = 5), children and adolescents (6–17 years; *n* = 5), adults (18–65 years; *n* = 8) and older adults (65+ years; *n* = 11). For those including children, all participants were diagnosed with a neurodevelopmental condition (ASC or ADHD), and for those including older adults, all participants were diagnosed with dementia.

In 28 studies (96.6%), just over half of all participants were female (*n* = 784, 54.9%). One study did not report participant gender.^68^ Only five studies (17.2%) reported ethnicity,^13,15,24,48,49,59^ and in these studies, two-thirds of participants were White Caucasians (*n* = 141, 66.5%). In 25 studies (86.2%) reporting participant age, the mean age was 42.7 years (s.d. = 32.8). Four studies (13.8%) did not provide information on participant age.^52,61,62,68^ For those reporting diagnosis severity at baseline data collection (*n* = 13, 44.8%), participants with dementia^16,57–65,69^ were most commonly diagnosed with mild, moderate or mild–moderate dementia (*n* = 678, 96.9%), and participants with schizophrenia^47,50^ were most commonly considered to be ‘mildly ill’ according to Positive and Negative Syndrome Scale scores (*n* = 64, 100%). Six studies (20.7%) reported participant characteristics related to animal ownership.^19,57,63–65,70^ Of the participants in these studies, 196 (68.7%) reported they were current or previous animal owners or enjoyed interaction with animals. Table 2 presents demographics reported by the studies.

**Table 2:** Participant demographics available in included studies, separated by age group

	Gender (*n*, %)	Ethnicity (*n*, %)	Mean age in years	Age (s.d.)	Experience with animals (*n*, %)
Children and adolescents					
Mental health conditions	Female (49, 56.3)	White (21, 63.6)	14.8	1.8	Not reported
Neurodevelopmental conditions	Female (85, 26.5)	White (83, 59.3)	8.7	2.4	Yes (18, 81.8)
Adults					
Mental health conditions	Female (120, 52.4)	Not reported	50.1	2.8	Not reported
Neurodevelopmental conditions	Female (46, 50.0)	Not reported	38.2	1.2	Yes (18, 33.9)
Older adults					
Dementia	Female (484, 69.1)	White (37, 94.9)	83.7	2.1	Yes (160, 76.2)

The majority of studies were conducted in Europe (*n* = 17; 58.6%), followed by Asia (*n* = 5; 17.2%), the USA (*n* = 4; 13.8%), Australia (*n* = 2; 6.9%) and the UK (*n* = 1; 3.5%). Study settings varied substantially and included hospitals (*n* = 11) and care facilities (e.g. nursing homes, care homes; *n* = 10). Supplementary Material 4 provides an overview of study characteristics.

### Intervention characteristics

Interventions varied by type (therapy or activity), content, role of intervention providers, group size, and frequency and duration (Supplementary Material 5). Of the 29 studies, DAIs included were described by authors as therapy (*n* = 23; 79.3%) and activities (*n* = 6; 20.7%). Studies used various controls, including the same therapy or activity without the presence of a dog,^13,15,24,48,49,51–53,55,61,63^ usual care activities or treatment,^14,16,18–20,22,47,50,60,62,64,65,68,71^ waiting-list control groups,^21,23^ relaxation or reminiscing interventions,^54,56,59^ and a discussion group about animals.^67^ Two studies did not provide detailed information regarding the control group content.^57,58^

#### Risk of bias assessment

**Fig. 2:**
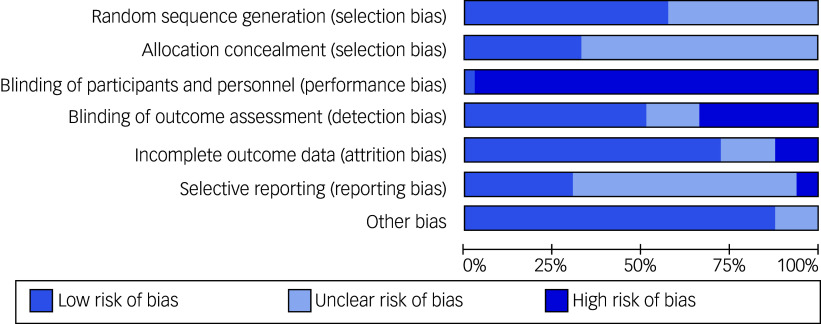
Risk of bias graph: review authors’ judgements about each risk of bias item presented as percentages across all included studies.

Risk of bias results are presented in Fig. 2. Thirteen studies (44.8%) were judged to show unclear risk of bias for random sequence generation owing to insufficient information regarding the method of randomisation (dementia, *n* = 5; neurodevelopmental, *n* = 4; mental health, *n* = 4). The remaining studies reported that participants were allocated using various methods (e.g. computer randomisation, coin-flip method) and were judged as being at low risk of bias. Twenty studies (69.0%) did not provide a statement regarding allocation concealment so were judged as having an unclear risk of bias (dementia, *n* = 10; mental health, *n* = 6; neurodevelopmental, *n* = 4). Twenty-eight studies (96.6%) reported in 33 papers were judged to show high risk of bias for blinding of participants and personnel owing to the inability to blind individuals to the presence of a dog. Only one study (3.5%) was judged to be of low risk of bias as both participants and personnel were blinded. Psychiatric rehabilitation institutions were randomised, and in those randomised to the control group, participants watched animal documentaries.^67^ No information was provided regarding whether the participants were debriefed about the blinding and their group allocation once participation had concluded.^67^

Just over half of the studies (*n* = 15; 51.7%) reported blinding outcome assessments and so were judged as having low risk of bias. However, nine studies (31.0%; reported in 12 papers focusing on different outcomes) did not blind outcome assessors and were judged as showing high risk of bias (mental health, *n* = 4; neurodevelopmental, *n* = 3; dementia, *n* = 2). Six of these reported that blinding was impossible owing to the nature of the intervention or limited resources,^14,19,47,50,64,65^ and four involved self-report or the child’s parents completing the outcome measures, so blinding could not be used.^13,15,48,52^ Five studies (17.2%) were judged as having unclear risk of bias owing to insufficient information related to blinding of outcome assessments (dementia, *n* = 4; neurodevelopmental, *n* = 1).

Most studies (*n* = 21; 72.4%) were judged as having low risk of bias for incomplete outcome data as more than 85% of participants completed the study. Four studies (13.8%) were judged as showing high risk of bias owing to withdrawals and exclusions that may have imbalanced groups and a lack of an intention-to-treat analysis and/or use of a per protocol analysis (mental health, *n* = 2; neurodevelopmental, *n* = 1; dementia, *n* = 1).^18,50,54,68^ The remaining four studies (13.8%) were judged as having unclear risk of bias owing to insufficient information (dementia, *n* = 2; mental health, *n* = 2).

Eighteen studies (62.1%; reported in 20 papers) were judged to have unclear bias regarding selective outcome reporting owing to the absence of a pre-published registration or protocol explicitly stating the primary outcomes and assessment time points (dementia, *n* = 9; neurodevelopmental, *n* = 6, mental health, *n* = 3). Two studies (6.9%) were judged to have high risk of bias for selective outcome reporting. One study reported an aim of investigating physiological and psychological aspects of schizophrenia, but no physiological measures were reported.^68^ One study reported that depression was measured pre- and post-intervention for participants with anxiety or mixed anxiety–depression disorders, but depression scores were not available.^52^ The remaining nine studies (31.0%; reported in 12 papers) were judged as showing low risk of bias as they cited a pre-published registration and/or protocol clearly stating the primary outcomes and assessment time points (neurodevelopmental, *n* = 6; mental health, *n* = 4; dementia, *n* = 2). Risk of bias across individual studies is presented in Supplementary Material 6.

#### What terminology and definitions are used to classify DAIs in the included RCTs?

For the 23 studies (79.3%) evaluating dog-assisted therapy, 20 (69.0%) were assessed as showing clear alignment of content and terminology (mental health, *n* = 5; neurodevelopmental, *n* = 9; dementia, *n* = 6) based on internationally accepted definitions.^45,46^ One study (3.4%) delivered to adults with schizophrenia was assessed as misaligned, as a member of the research team delivered the sessions, no goals were reported and the sessions were described as ‘activities’.^50^ Last, two studies (6.9%) delivered to participants with dementia were assessed as having unclear alignment, as limited information was reported on the training or experience of the dog-handler team and on how content was developed to meet goals.^58,61^ All six of the studies reporting dog-assisted activities (100%) were classed as showing clear alignment (mental health, *n* = 2; neurodevelopmental, *n* = 1; dementia, *n* = 3). Supplementary Material 7 presents content from studies that describes details related to goals and/or content and the dog-handler team, and whether the study was assessed as having clear alignment, unclear alignment or misalignment.

#### What is the effectiveness of DAIs for populations with mental health and neurodevelopmental conditions?

Studies included a wide range of mental health and behavioural outcome measures (Supplementary Material 4), most commonly evaluating depression (*n* = 14; 48.3%), social skills (*n* = 14; 48.3%), symptom frequency and/or severity (*n* = 10; 34.5%) and agitation (*n* = 6; 20.7%). For all of the commonly reported outcomes, findings were mixed. Although this is likely to have been due to the small sample sizes, it may also be attributable to the diverse range of DAIs delivered, as they varied considerably by type (therapy or activity), characteristics of provision (such as group size, frequency and duration) and intervention content (Supplementary Material 5). There was also substantial variation in intervention intensity (Table 3), with total intervention intensity ranging from 0.3 to 48 h for participants with mental health conditions, 3 to 54 h for participants with neurodevelopmental conditions, and 8 to 70 h for participants with dementia.

**Table 3:** Intervention frequency, duration and intensity for each study, and average intervention intensity for dementia, neurodevelopmental conditions and mental health conditions

Authors (year)	Duration, weeks	Frequency	Session length, min	Intervention intensity, h	Average intervention intensity, h
Mental health conditions					
Allen et al (2021)^24^	12	1 × weekly	90	18	14.8
Calvo et al (2016)^50^	24	2 × weekly	60	48	
Chen et al (2021; 2022)^14,47^	12	1 × weekly	60	12	
Chu et al (2009)^68^	8	1 × weekly	50	7	
Shih et al (2023)^67^	12	1 × weekly	60	12	
Stefanini et al (2015)^66^	12	1 × weekly	45	9	
Stefanini et al (2016)^20^	12	1 × weekly	45	9	
Villalta-Gil et al (2009)^51^	12	2 × weekly	45	18	
Wolynczyk-Gmaj et al (2021)^52^	1	Once	20	0.3	
Neurodevelopmental disorders					
Fung et al (2014)^53^	7	3 × weekly	20	7	18.3
Hill et al (2020)^19^	9	1 × weekly	60	9	
Meints et al (2022)^54^	4	2 × weekly	20	3	
Scorzato et al (2017)^55^	20	1 × weekly	30	10	
Schuck et al (2015)^13^	12	2 × weekly	120/150	54	
Schuck et al (2018; 2018)^15,48^, Nieforth et al (2024)^49^	12	2 × weekly	120/150	54	
Vidal et al (2020)^71^	12	1 × weekly	45	9	
Vidal et al (2023)^56^	12	1 × weekly	45	9	
Wijker et al (2020; 2021)^23,70^	10	1 × weekly	60	10	
Dementia					
Baek et al (2020)^57^	8	2 × weekly	60	16	25
Bono et al (2015)^58^	35	2 × weekly	60	70	
Briones et al (2021)^69^	39	1 × weekly	50	33	
Friedmann et al (2015)^59^	12	2 × weekly	90	36	
Majic et al (2013)^60^	10	1 × weekly	45	8	
Menna et al (2019)^61^	12	1 × weekly	NS	N/A	
Olsen et al (2016a)^64^	12	2 × weekly	30	12	
Olsen et al (2016b)^65^	12	2 × weekly	30	12	
Parra et al (2021)^62^	35	1 × weekly	45	26	
Parra et al (2022)^16^	26	1 × weekly	45	20	
Travers et al (2015)^63^	11	2 × weekly	45	17	

NS, not specified; N/A, not applicable.

### Depression

Fourteen studies (48.3%) reported depression as an outcome (dementia, *n* = 10; mental health, *n* = 3; neurodevelopmental, *n* = 1). The measures used to assess depression varied (Supplementary Material 4), but for participants with dementia, depression was most commonly evaluated using the Cornell Scale for Depression in Dementia (*n* = 5) or the Geriatric Depression Scale (*n* = 3).

Six studies showed a positive impact on depression compared with the control group (dementia, *n* = 5; mental health, *n* = 1).^16,20,58,61,62,65^ Of these, five evaluated individual or group dog-assisted therapy delivered by a professionally trained animal handler, but in only was the handler accompanied by an experienced therapist.^16^ The remaining study evaluated group dog-assisted activities delivered by a professionally trained animal handler.^65^ Seven studies showed no benefits of DAIs in terms of depression scores compared with the control group (dementia, *n* = 4; mental health, *n* = 2; neurodevelopmental, *n* = 1).^24,47,57,59,60,63,71^ Of these, six evaluated group or individual dog-assisted therapy delivered by an experienced therapist and professional animal handler,^47,63,71^ a professional animal handler alone,^57,60^ or an experienced clinician trained by an animal handler.^24^ One evaluated group dog-assisted activities delivered by a staff nurse.^59^ Last, one study aimed to evaluate depression, but post-intervention depression scores were not reported.^52^

**Fig. 3:**
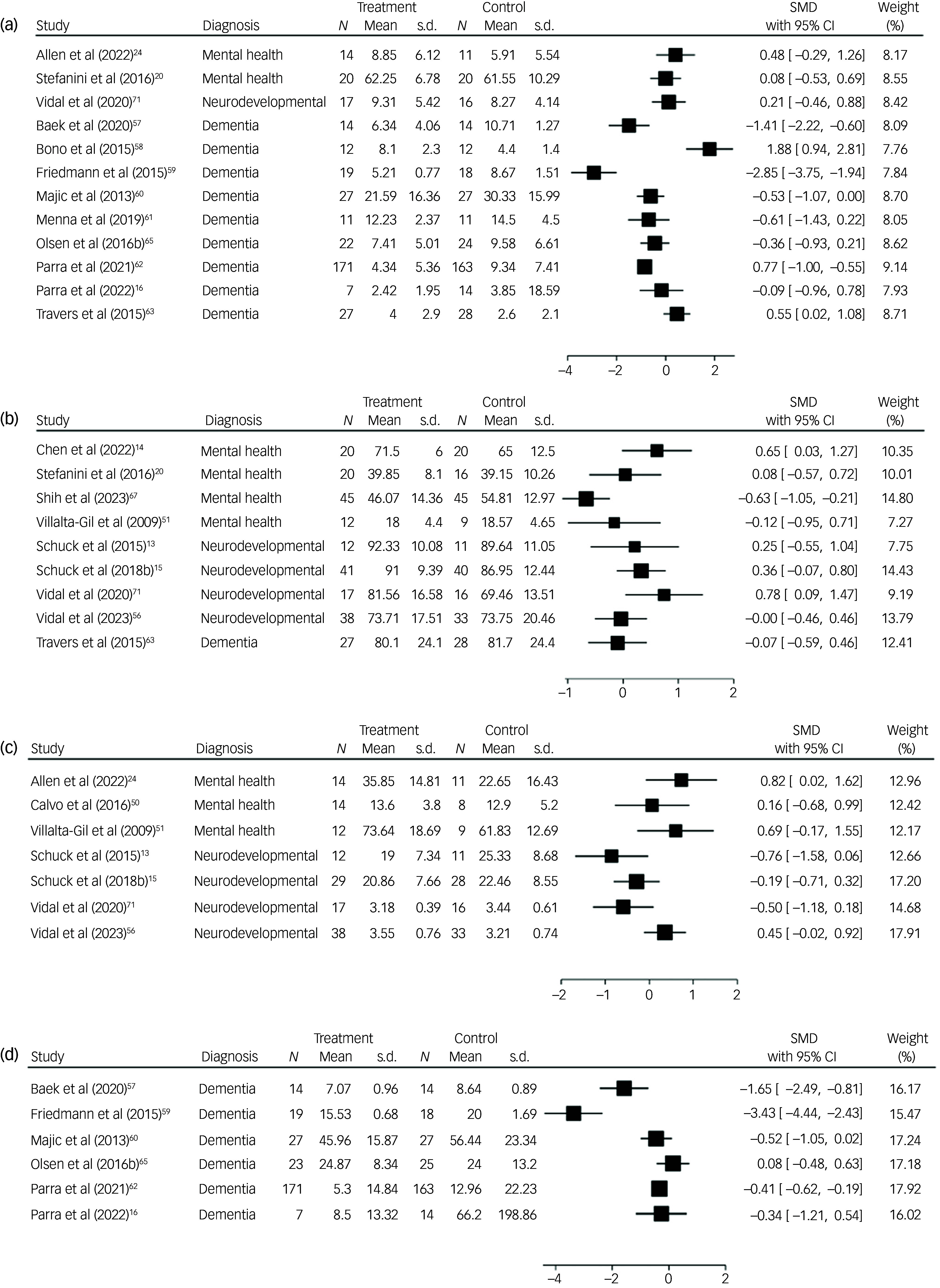
Forest plots for comparison of depression (a), social skills and/or behaviour (b), symptom frequency and/or severity (c) and agitation (d) at longest follow-up. SMD, standardised mean difference.

A forest plot showing a comparison of depression at longest follow-up is presented in Fig. 3(a). As improvement in depressive symptoms was associated with lower scores on all outcome measures, SMDs less than zero indicate improvements for the intervention arm. Of the 14 studies evaluating this outcome, two trials were excluded, as one did not report mean values or standard deviations,^47^ and the other did not provide post-intervention depression scores.^52^

### Social skills and/or behaviour

Fourteen studies (48.3%) evaluated the impact of DAIs on social skills and/or behaviour using various measures (Supplementary Material 4). Of these, eight studies showed a positive impact of group dog-assisted therapy on social skills and/or behaviour compared with the control group (neurodevelopmental, *n* = 6; mental health, *n* = 2).^13–15,49,55,56,67,71^ Interventions in all eight studies were delivered by a professional animal handler, and in three, the handler was accompanied by an experienced therapist or psychologist.^14,56,71^

Six studies showed no benefits of group or individual dog-assisted therapy with respect to social skills and/or behaviour compared with the control group (mental health, *n* = 3; neurodevelopmental, *n* = 2; dementia, *n* = 1).^20,21,51,53,63,66^ Of these, four involved interventions delivered by a professional animal handler.^20,51,53,66^ In one, the handler was accompanied by an experienced psychologist,^51^ and two were delivered by a therapist who had been trained in dog behaviour and welfare, working with either their own accredited dogs^63^ or dogs provided by a service dog foundation.^21^

A forest plot showing a comparison of social skills and/or behaviours at longest follow-up is presented in Fig. 3(b). As improvement in social skills and/or behaviour was associated with higher scores on all outcome measures, SMDs greater than zero indicate improvements for the intervention arm. Of the 14 studies evaluating this outcome, five trials were excluded, as post-intervention means and standard deviations were not reported.^21,49,53,55,66^

### Symptom frequency and/or severity

Ten studies (34.5%) measured changes in symptom frequency and/or severity. Measures varied by diagnosis (Supplementary Material 4); however, all those involving interventions delivered to participants with schizophrenia evaluated symptomology using the Positive and Negative Syndrome Scale, and those involving participants with ADHD used the ADHD Rating Scale. Five studies showed a positive impact of DAIs on symptom frequency and/or severity compared with the control group (neurodevelopmental, *n* = 3; mental health, *n* = 2).^13,15,47,68,71^ Of these, four studies evaluated group dog-assisted therapy delivered by a professional animal handler and an experienced therapist or psychologist^47,71^ or a professional animal handler alone.^13,15^ The remaining study evaluated group dog-assisted activities delivered by a member of the research team.^68^

Five studies showed no benefits of dog-assisted therapy in terms of symptom frequency and/or severity compared with the control group (mental health, *n* = 3; neurodevelopmental, *n* = 2).^24,50,51,56,70^ Of these, one intervention was delivered on an individual basis by experienced therapists who had completed advanced courses in dog behaviour and welfare,^21^ one was group-based and delivered by a professional animal handler,^50^ one was delivered to groups and on an individual basis by a professional animal handler and psychologist,^56^ and two were delivered by experienced clinicians trained by an animal handler to a group or on an individual basis, respectively.^24,51^

A forest plot showing a comparison of symptom frequency and/or severity at longest follow-up is presented in Fig. 3(c). As improvement in symptom frequency and/or severity was associated with lower scores on all outcome measures, SMDs less than zero indicate improvements for the intervention arm. Of the ten studies evaluating this outcome, three trials were excluded from the forest plot, as means and standard deviations were not reported.^21,47,68^

### Agitation

Six studies (20.7%) of participants with dementia evaluated the impact of DAIs on agitation using various measures (Supplementary Material 4). Two studies showed a positive impact of dog-assisted therapy compared with the control group,^16,62^ and in both, interventions were delivered by a professional animal handler and experienced therapist. However, four studies showed no benefits of DAIs with respect to agitation compared with the control group.^57,59,60,65^ Of these, two evaluated group dog-assisted activities delivered by a professional animal handler^65^ or a nurse practitioner.^59^ Two evaluated dog-assisted therapy delivered by a professional animal handler only; in one of these, the therapy was delivered on a group basis,^57^ and the other did not specify whether the sessions were group-based or on a one-to-one basis.^60^

A forest plot showing a comparison of agitation at longest follow-up is presented in Fig. 3(d). As improvement in agitation was associated with lower scores on all outcome measures, SMDs less than zero indicate improvements for the intervention arm.

#### How well reported are RCTs delivering DAIs to people with mental health and neurodevelopmental conditions?

The mean proportion of adherence to the CONSORT statement was calculated to be 48.6% with a standard deviation of 13.4% (minimum and maximum adherence proportions were 13.51% and 75.7%, respectively). Only nine items were reported in more than 75% of the included RCTs. Notably, 17 papers (51.5%) were published across 13 journals that did not explicitly require authors to follow the CONSORT statement.

**Fig. 4:**
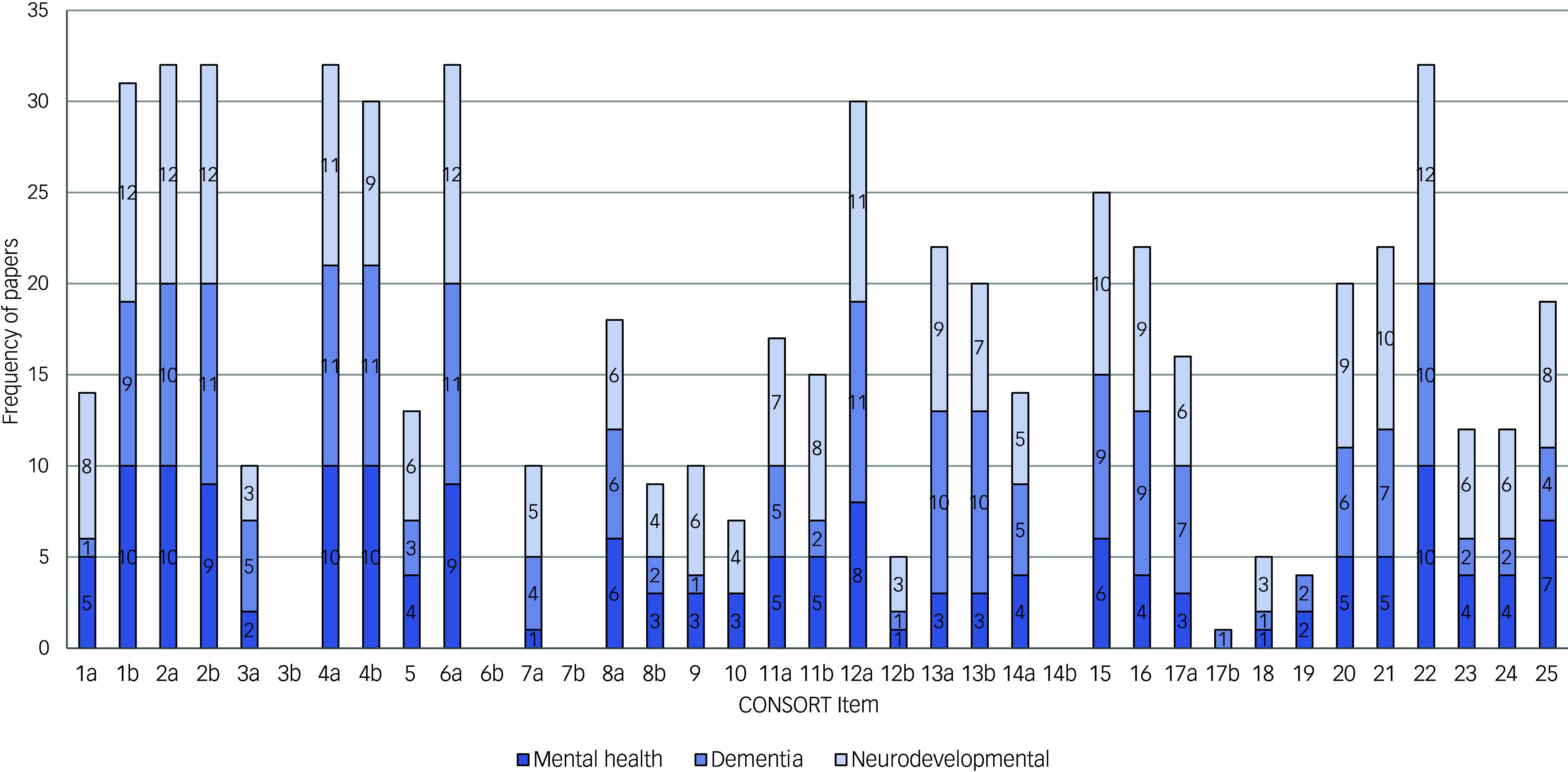
Graphical presentation of CONSORT compliance per item and by diagnosis category.

Compliance per CONSORT item is presented in Table 4 and Fig. 4. Overall, Cohen’s kappa indicated a statistically significant ‘strong’ level of agreement (κ = 0.88 (95% CI = 0.53–1.23, *P* < 0.001). Cohen’s kappa was also calculated to assess the agreement by CONSORT item (Table 4). Four items were assessed as ‘no’, as they were not applicable to the included RCTs. These included changes to methods after trial commencement, changes to trial outcomes after the trial commenced, explanation of any interim analyses and stopping guidelines, and why the trial ended or was stopped. In addition, many of the RCTs did not report binary outcomes, so item 17b (presentation of effect sizes for binary outcomes) was not applicable to the majority of the studies (*n* = 23). The lowest scoring item was ‘important harms or unintended effects’ (item 19; *n* = 4, 12.1%).

**Table 4:** Assessment of the quality of reporting of randomised controlled trials using the CONSORT statement

Item; subitem	Item	Checklist	Number of randomised controlled trials (%)	Cohen’s κ	Significance (*P*-value)
Title and abstract					
	1a	Identification as a randomised controlled trial in the title	14 (42.4)	1.00	0.001
	1b	Structured summary of trial design, methods, results and conclusions	31 (93.9)	1.00	0.001
Introduction					
Background	2a	Scientific background and explanation of rationale	32 (96.9)	1.00	0.001
Objectives	2b	Specific objectives or hypotheses	32 (96.9)	1.00	0.001
Methods					
Trial design	3a	Description of trial design (such as parallel, factorial) including allocation ratio	10 (30.3)	0.82	0.001
	3b	Important changes to methods after trial commencement (such as eligibility criteria), with reasons	0 (0)	1.00	0.001
Participants	4a	Eligibility criteria for participants	32 (96.9)	1.00	0.001
	4b	Settings and locations where the data were collected	30 (90.9)	1.00	0.001
Interventions	5	Interventions for each group with sufficient details to allow replication, including how and when they were actually administered	13 (39.4)	0.64	0.071
Outcomes	6a	Completely defined prespecified primary and secondary outcome measures, including how and when they were assessed	32 (96.9)	1.00	0.001
	6b	Any changes to trial outcomes after the trial commenced, with reasons	0 (0)	1.00	0.001
Sample size	7a	How sample size was determined	10 (30.3)	1.00	0.001
	7b	When applicable, explanation of any interim analyses and stopping guidelines	0 (0)	1.00	0.001
Randomisation: sequence generation	8a	Method used to generate the random allocation sequence	18 (54.6)	0.82	0.001
	8b	Type of randomisation; details of any restriction (such as blocking and block size)	9 (27.3)	1.00	0.001
Randomisation: allocation concealment mechanism	9	Mechanism used to implement the random allocation sequence (such as sequentially numbered containers), describing any steps taken to conceal the sequence until interventions were assigned	10 (30.3)	1.00	0.001
Randomisation: implementation	10	Who generated the random allocation sequence, who enrolled participants and who assigned participants to interventions	7 (21.2)	1.00	0.001
Blinding	11a	If done, who was blinded after assignment to interventions (for example, participants, care providers, those assessing outcomes) and how	17 (51.5)	1.00	0.001
	11b	If relevant, description of the similarity of interventions	15 (45.5)	0.64	0.071
Statistical methods	12a	Statistical methods used to compare groups for primary and secondary outcomes	30 (90.9)	1.00	0.001
	12b	Methods for additional analyses, such as subgroup analyses and adjusted analyses	5 (15.2)	1.00	0.001
Results					
Participant flow	13a	For each group, numbers of participants who were randomly assigned, received intended treatment and were analysed for the primary outcome	22 (66.7)	0.93	0.001
	13b	For each group, losses and exclusions after randomisation, together with reasons	20 (60.6)	1.00	0.001
Recruitment	14a	Dates defining the periods of recruitment and follow-up	14 (42.4)	1.00	0.001
	14b	Why the trial ended or was stopped	0 (0)	1.00	0.001
Baseline data	15	A table showing baseline demographic and clinical characteristics for each group	25 (75.8)	0.93	0.001
Numbers analysed	16	For each group, number of participants (denominator) included in each analysis and whether the analysis was by original assigned groups	22 (66.7)	0.93	0.001
Outcomes and estimation	17a	For each primary and secondary outcome, results for each group and the estimated effect size and its precision (such as 95% confidence interval)	16 (48.5)	0.93	0.001
	17b	For binary outcomes, presentation of both absolute and relative effect sizes is recommended	1 (3.0)	1.00	0.001
Ancillary analyses	18	Results of any other analyses performed, including subgroup analyses and adjusted analyses, distinguishing prespecified from exploratory	5 (15.2)	0.82	0.001
Harms	19	All important harms or unintended effects in each group	4 (12.1)	0.93	0.001
Discussion					
Limitations	20	Trial limitations, addressing sources of potential bias, imprecision and, if relevant, multiplicity of analyses	20 (60.6)	0.76	0.001
Generalisability	21	Generalisability (external validity, applicability) of the trial findings	22 (66.7)	0.82	0.001
Interpretation	22	Interpretation consistent with results, balancing benefits and harms, and considering other relevant evidence	32 (96.9)	1.00	0.001
Other information					
Registration	23	Registration number and name of trial registry	12 (36.4)	1.00	0.001
Protocol	24	Where the full trial protocol can be accessed, if available	12 (36.4)	1.00	0.001
Funding	25	Sources of funding and other support (such as supply of drugs), role of funders	19 (57.6)	1.00	0.001

For studies that reported important harms or unintended effects, three (10.3%) reported adverse events related to the DAI. These included treatment-disrupting events due to the dog,^24^ participants exhibiting behaviours that threatened to compromise the welfare of the dog^50^ and participants presenting fearful reactions to the dog.^60^ One study reported an adverse event unrelated to the DAI, indicating that an infectious outbreak may have negatively influenced outcomes.^63^ Although adverse events related to DAIs were reported in only three studies (10.3%), selection criteria for the dogs were reported in 20 studies (69.0%; e.g. free of veterinary infectious diseases, certified in accordance with a national standard, completion of vaccinations, previous participation in DAIs, and appropriate ratings on aptitude and temperament tests). Ten studies (34.5%) specifically reported information about training, in varying detail. Eight reported only that dogs were trained to work with people,^24,50,58,60,64,68,70,71^ whereas two reported information about the dog being trained on specific exercises of the intervention.^19,62^ Information on dog safety and welfare was most commonly reported in studies delivering interventions to participants with neurodevelopmental conditions. These studies reported that the dogs’ working time was limited per day,^23,53,54,70^ and/or dog welfare and stress behaviours were documented or monitored.^23,54,55,70^

Fewer than half of the RCTs (*n* = 13, 39.4%) adequately reported details relating to the intervention according to the CONSORT statement, and further assessment using the TIDieR checklist indicated considerable variability in intervention reporting (Table 5). Only one of the 33 papers reported all of the information expected.^19^ Items most likely to achieve a ‘yes’ agreement included intervention name (100%), rationale (100%), procedures and processes (100%) and frequency (100%). Those least likely to achieve a ‘yes’ agreement included items relating to the description of the intervention provider (51.5%), location (51.5%) and materials (18.2%).

**Table 5:** Frequency of papers achieving ‘yes’, ‘no’ or ‘N/A’ agreement for each Template for Intervention Description and Replication (TIDieR) checklist item

TIDieR checklist item	*n*	Yes, % (*n*)	No, % (*n*)	N/A, % (*n*)	Cohen’s κ	Significance (*P*-value)
Name or phrase describing the intervention	33	100 (33)	0 (0)	0 (0)	1.00	0.001
Intervention rationale	33	100 (33)	0 (0)	0 (0)	1.00	0.001
Description of intervention materials	33	18.2 (6)	81.8 (27)	0 (0)	1.00	0.001
Intervention procedures and processes	33	100 (33)	0 (0)	0 (0)	1.00	0.001
Intervention provider	33	51.5 (17)	48.5 (16)	0 (0)	0.93	0.001
Mode(s) of delivery	33	87.9 (29)	12.1 (4)	0 (0)	1.00	0.001
Intervention location	33	51.5 (17)	48.5 (16)	0 (0)	1.00	0.001
Intervention frequency	33	100 (33)	0 (0)	0 (0)	1.00	0.001
If undertaken, tailoring of the intervention	33	6.1 (2)	30.3 (10)	63.6 (21)	0.74	0.005
If undertaken, modification of the intervention	0	N/A	N/A	N/A	N/A	N/A
How intervention adherence or fidelity was assessed where appropriate	33	12.2 (4)	3.0 (1)	84.8 (28)	1.00	0.001
If undertaken, actual intervention adherence or fidelity	33	15.2 (5)	0 (0)	84.8 (28)	1.00	0.00

N/A, not applicable.

Overall, Cohen’s kappa indicated a statistically significant ‘almost perfect’ level of agreement (κ = 0.99 (95% CI = 0.97–1.01, *P* < 0.001). Cohen’s kappa was also calculated to assess agreement by TIDieR checklist item (Table 5).

## Discussion

The aims of this review were to synthesise findings of published research to determine whether DAIs are effective for people with mental health or neurodevelopmental conditions and to formally assess the quality of reporting and use of terminology in RCTs for the first time. Findings for the effectiveness of DAIs across outcome categories were mixed, as determined using direction of effect and forest plots. However, they clearly signalled promise and indicated opportunities to improve future research in this area (e.g. through the development of guidelines for clear terminology and reporting standards, and through rigorous RCTs with larger sample sizes to ensure studies are adequately powered). Owing to small sample sizes, heterogeneity of study quality and outcome measures, and variation in the types of DAI provided (in terms of content and delivery), it was challenging or impossible to interpret results in terms of the potential benefits of DAIs for a specific population. However, taking into consideration the three core outcome groups (depression, social skills and agitation, recognising that symptom frequency and/or severity is not symptom specific), 57% (8/14) of the studies reported positive outcomes of DAI for social skills, whereas 43% (6/14) reported positive outcomes for depression and 33% (2/6) for agitation. Of 14 studies evaluating social skills, only two were rated as overall high quality;^56,71^ both of these reported positive outcomes. Of the 14 studies evaluating depression, only one study was rated as overall high quality,^24^ and no benefits of the DAI were reported. No studies evaluating agitation were rated as overall high quality.

Without further investigation of potential mechanistic pathways, which would be beyond the scope of this review, we therefore tentatively propose that DAIs show particular promise for conditions that might benefit from social skill support. Future research should investigate potential mechanisms of action of DAIs (and AAIs in general) in greater detail, so these can be closely linked to specific outcome measures and populations, including hypotheses involving longer-term impact beyond intervention completion. It will be important to justify any hypotheses more rigorously according to which symptoms of mood disorders (e.g. depression or anxiety) would still be improved 6 months post-intervention, considering any mediating or ‘catalysing’ factors such as improved engagement and rapport-building facilitated by the DAI compared with standard care.

### Methodological considerations

Whereas some RCTs found improvements for depression, social skills and/or behaviour, symptom frequency and/or severity, and agitation, other trials did not find benefits of DAIs with respect to these outcomes. Although the quality of the evidence base is improving, there is largely an absence of the rigorous methodology that would enable demonstration of the potential effectiveness of DAIs. For example, the studies frequently included small or very small sample sizes, rendering studies inadequately powered to detect potential differences in effect sizes between study groups and probably undermining the internal and external validity of the studies.^72^ Other examples of limited rigour include generally short follow-up periods, or no follow-up, for assessing outcomes and an overall high risk of bias for the majority of included studies (*n* = 25).

In addition, there were several limitations in relation to generalisability to our study population groups. First, for children and adolescents with neurodevelopmental conditions, females were notably underrepresented (*n* = 85, 26.5%). This could have been because males are more likely to be diagnosed with ASC or ADHD than females;^73,74^ future research should aim to include more female participants to adequately reflect the population of children and young people with neurodevelopmental conditions.^75^ Second, only five studies reported information regarding participant ethnicity. Collection and reporting of ethnicity data are essential for understanding the generalisability of findings and the probable impact of an intervention for particular ethnic groups.^76^ Likewise, information regarding the severity of a participant’s condition was only reported for those with dementia or schizophrenia. As severity is not consistently reported, it cannot be determined whether the effects of DAIs should be attributed to the intervention or the severity of the condition.^77^ This limitation has been highlighted in previous systematic reviews exploring AAIs for ASC^75^ and schizophrenia.^78^

Although our findings cannot offer definitive conclusions about the effectiveness of DAIs for our study population groups, they clearly signal the potential of DAIs to improve a variety of psychosocial outcomes, consistent with findings from previous observational studies.^79–82^ Recent evidence syntheses also indicate the potential of DAIs to improve outcomes for a range of mental health and neurodevelopmental conditions, including schizophrenia,^78^ mental health conditions,^8,83,84^ post-traumatic stress disorder and trauma,^9,85^ ASC^11,34^ and ADHD.^86^ Despite this, evidence syntheses have unanimously emphasised the need for more rigorous and sufficiently powered RCTs^25,78,87,88^ to determine the true impact of DAIs for these populations. However, the focus on determining effectiveness raises an important issue: reporting of RCTs of DAIs is often insufficiently accurate, comprehensive and transparent. For example, authors often have not reported data on intervention implementation (e.g. adaptation or tailoring of the intervention to specific groups or materials used to support intervention implementation). Inadequate reporting can make it challenging for researchers to replicate trials, for intervention developers to design effective interventions and for providers to implement interventions in practice.^89^ A lack of sharing of protocols, outcome data and intervention materials may have limited the ability of human–animal interaction researchers to reproduce trial procedures, replicate trial results and effectively synthesise evidence on these interventions.^90^

The present review also found that many CONSORT items were poorly reported in the DAI literature. Such items included descriptions of trial design, information about how sample size was determined, randomisation information, and important harms or unintended effects in each group. Only 14 of 27 journals included referenced reporting guidelines in their instructions to authors. This inefficient use of resources for research has probably contributed to suboptimal dissemination of potentially effective interventions and overestimation of intervention efficacy. As in other areas of research, transparent, detailed and adequately subject-specific reporting of DAI RCTs is needed to minimise reporting biases and maximise the credibility and utility of this research evidence.^91^

### Beyond effectiveness

It is important to extend this focus beyond ‘what works’ and consider ‘under what circumstances and how these interventions work’.^92^ The effect of complex DAIs (or AAIs generally), which involve poorly understood interspecies interactions between several actors including a dog, may depend on elements of difficult-to-control, dynamic systems in which they occur.^93^ For example, aspects related to the physical environment in which interventions take place, which may vary greatly between or even within study settings but may have substantial effects on the dogs involved; considerations relating to ‘matching’ dogs and participants, and the role of all actors involved (participant, handler and/or therapist) would be important to investigate. To unlock the true potential of DAIs (and AAIs generally) in the future, it will be crucial to complement evidence from applied intervention research with findings from well-designed and well-conducted observational studies focused on exploring layers of AAIs/DAIs (such as mechanistic impact-outcome pathways; environmental aspects; the role of all actors, and interspecies reciprocity)^94^ that have so far received little attention but will be fundamental in advancing this promising area. Future research needs to explore how and why these interventions work, for whom, and under what conditions.^95^ Interdisciplinary mixed-method research and process evaluations conducted alongside outcome evaluations could facilitate our understanding of how DAIs may work and highlight issues that may impact effectiveness in real-world settings.

### Intervention terminology, practice and reporting

Despite expansion of practice, inconsistencies remain in how DAIs are described, practised, and reported upon within the evidence base.^32^ While most DAIs described in studies in this review were assessed as having clear alignment for content and terminology, improvement in reporting certain information was still required (e.g., training of the dog-handler team, measures used to assess dog aptitude, temperament and behaviour, access to intervention materials to identify how content was developed to align with goals, in the case of therapy). The absence of this detailed information makes it challenging to ascertain the preparation, training, and expectations of the handler and the dogs that work in different roles. Recent research has argued these difficulties may have hindered the development of the field in terms of establishing agreed standards of practice, qualifications and competencies, and adopting good animal welfare practices.^32^ As a result, new uniform terminology has been suggested to improve clarify for those involved in the delivery and receipt of DAIs.^32^ This review uses original terminology to be consistent with the taxonomy and definitions reported in the included RCTs. Seeing the extensive variety of intervention content, engagement and delivery modalities reported for DAIs (Supplementary Material 5), future work could usefully focus on efforts to classify further subtypes of DAIs, building on the classification by Binder et al,^32^ specifying the role of the dog and type of intervention content. This would allow future evidence syntheses to summarise study findings more specifically in relation to the effectiveness of ‘DAI types’ for specific populations and would further facilitate our understanding of what works for whom under what circumstances.

### Limitations

First, the clinical and methodological heterogeneity did not allow for meta-analyses to definitively determine the benefits of DAIs for participants with mental health or neurodevelopmental conditions. Analyses to separate studies by those evaluating dog-assisted therapy and those evaluating dog-assisted activities were considered. However, owing to a number imbalance, this was not possible. For example, only two of the 14 studies evaluating depression involved dog-assisted activities (compared the 12 which involved therapy). For studies evaluating symptom frequency and/or severity, only one of ten studies evaluated dog-assisted activity, and all studies evaluating social skills delivered dog-assisted therapy only. Therefore, the effectiveness results should be interpreted with some caution. Second, although this review aimed to determine the effectiveness of DAIs for individuals with neurodevelopmental and mental health conditions across all age groups, a significant proportion of the studies included focused on older participants with dementia. Subsequently, the findings related to depression and agitation are not generalisable to populations of younger individuals with mental health or neurodevelopmental conditions. Future research targeting these subgroups is required to clarify the impact of DAIs across diverse age ranges and conditions. Last, only papers published in English were included; inclusion of non-English-language studies may have contributed to further understanding.

### Future implications

The implementation of DAIs for a wide range of mental health and neurodevelopmental conditions has been rapidly increasing in practice. The existing body of evidence indicates that DAIs may have the potential to improve mental health and behavioural outcomes for these population groups, possibly specifically for conditions that benefit from improved social skills; however, there are considerable methodological concerns regarding the current literature. There remains significant room for improvement in relation to the design and reporting of DAI RCTs, with the potential to develop DAI (or AAI)-specific extensions to existing guidelines. Further rigorous interdisciplinary research is required to help advance research in this field.

## Supporting information

Shoesmith et al. supplementary material 1 Shoesmith et al. supplementary material

Shoesmith et al. supplementary material 2 Shoesmith et al. supplementary material

Shoesmith et al. supplementary material 3 Shoesmith et al. supplementary material

Shoesmith et al. supplementary material 4 Shoesmith et al. supplementary material

Shoesmith et al. supplementary material 5 Shoesmith et al. supplementary material

Shoesmith et al. supplementary material 6 Shoesmith et al. supplementary material

Shoesmith et al. supplementary material 7 Shoesmith et al. supplementary material

## Data Availability

Data availability is not applicable to this article as no new data were created or analysed in this study.
