# Supplementary material for: Dog-assisted interventions for children and adults with mental health or neurodevelopmental conditions: systematic review
Source: Br J Psychiatry. 2025 Apr 14;228(2):150–63. doi: 10.1192/bjp.2025.8 (PMC7617605; doi:10.1192/bjp.2025.8)
Supplement: Shoesmith et al. supplementary material 1 — Shoesmith et al. supplementary material [file S000712502500008Xsup001.docx]

**Supplementary Material 1: Search Strategy for EMBASE**

1. Emot$ OR ment$ OR psychological$ OR psychiatr$ OR risk OR disorder
2. ((Chronic$ or severe$ or persistent$) adj (mental$ OR psychological$) adj (disorder$ or ill$))
3. Exp Neuro$ Disorders/
4. Exp Neurodevelopmental Disorders/
5. Exp Intellectual Disability/
6. Exp autism spectrum disorder/ OR exp autistic disorder/
7. Autistic or autism or Asperger$
8. Exp Attention deficit disorder/ OR disruptive behaviour/ OR Attention deficit disorder with hyperactivity/
9. 1 OR 2 OR 3 OR 4 OR 5 OR 6 OR 7 OR 8
10. ((pet$ OR animal$ OR dog$ or canine$) adj2 (therap$ or activit$ or intervention$))
11. Exp animal assisted intervention/
12. Therap$ pet
13. Therap$ animal
14. Therap$ dog
15. Human animal bond$
16. Human animal interaction$
17. ((Assistance or service) adj2 (animal$ OR dog$))
18. 10 OR 11 OR 12 OR 13 OR 14 OR 15 OR 16 OR 17
19. 9 and 18
20. Limit 19 to English Language
