# Supplementary material for: Dog-assisted interventions for children and adults with mental health or neurodevelopmental conditions: systematic review
Source: Br J Psychiatry. 2025 Apr 14;228(2):150–63. doi: 10.1192/bjp.2025.8 (PMC7617605; doi:10.1192/bjp.2025.8)
Supplement: Shoesmith et al. supplementary material 2 — Shoesmith et al. supplementary material [file S000712502500008Xsup002.docx]

**Supplementary Material 2.** Complete list of data extracted from included papers

| 1 | Author/Year |
| --- | --- |
| 2 | Country |
| 3 | Research design |
| 4 | Registration number/name of trial registry |
| 5 | Setting |
| 6 | Participant demographics (e.g., age, gender, ethnicity) |
| 7 | Sample size (intervention/control) |
| 8 | Participant diagnosis |
| 9 | Diagnostic criteria |
| 10 | Dog-related inclusion/exclusion criteria |
| 11 | Recruitment rate |
| 12 | Attrition rate |
| 13 | Intervention attendance |
| 14 | Randomisation procedure |
| 15 | Blinding |
| 16 | Intervention name (e.g., therapy, activity) |
| 17 | Intervention content (including any tailoring/adaptation) |
| 18 | Intervention frequency/duration |
| 19 | Intervention space |
| 20 | Mode of delivery (e.g., group/individual) |
| 21 | Number and breed of dogs involved |
| 22 | Facilitator role |
| 23 | Facilitator training |
| 24 | Reference to infection control |
| 25 | Dog selection process |
| 26 | Dog training process |
| 27 | Information related to dog safety |
| 28 | Adverse events |
| 29 | Control name |
| 30 | Control content |
| 31 | Control frequency/duration |
| 32 | Control space |
| 33 | Control mode of delivery (e.g., group/individual) |
| 34 | Control facilitator role |
| 35 | Control facilitator training |
| 36 | Follow-up length |
| 37 | Outcome measures |
| 38 | Key findings |
| 39 | Key outcome means and standard deviations |
| 40 | Reported barriers |
| 41 | Reported enablers |
| 42 | Perceptions of intervention |
| 43 | Source of funding/other support |
