# Supplementary material for: Dog-assisted interventions for children and adults with mental health or neurodevelopmental conditions: systematic review
Source: Br J Psychiatry. 2025 Apr 14;228(2):150–63. doi: 10.1192/bjp.2025.8 (PMC7617605; doi:10.1192/bjp.2025.8)
Supplement: Shoesmith et al. supplementary material 3 — Shoesmith et al. supplementary material [file S000712502500008Xsup003.docx]

**Supplementary Material 3.** List of included papers (1-33)

1. Allen B, Shenk CE, Dreschel NE, Wang M, Bucher AM, Desir MP, et al. Integrating Animal-Assisted Therapy Into TF-CBT for Abused Youth With PTSD: A Randomized Controlled Feasibility Trial. Child maltreatment. 2022;27(3):466-77.

2. Baek SM, Lee Y, Sohng KY. The psychological and behavioural effects of an animal-assisted therapy programme in Korean older adults with dementia. Psychogeriatrics. 2020;20(5):645-53.

3. Bono AV, Benvenuti C, Buzzi M, Ciatti R, Chiarelli V, Chiambretto P, et al. Effects of animal assisted therapy (AAT) carried out with dogs on the evolution of mild cognitive impairment. Giornale di Gerontologia. 2015;63(1):32-6.

4. Briones MA, Pardo-Garcia I, Escribano-Sotos F. Effectiveness of a dog-assisted therapy program to enhance quality of life in institutionalized dementia patients. Clinical Nursing Research. 2021;30(1):89-97.

5. Calvo P, Fortuny JR, Guzmán S, Macías C, Bowen J, García ML, et al. Animal assisted therapy (AAT) program as a useful adjunct to conventional psychosocial rehabilitation for patients with schizophrenia: results of a small-scale randomized controlled trial. Frontiers in psychology. 2016;7:631.

6. Chen CR, Hung CF, Lee YW, Tseng WT, Chen ML, Chen TT. Functional Outcomes in a Randomized Controlled Trial of Animal-Assisted Therapy on Middle-Aged and Older Adults with Schizophrenia. International Journal of Environmental Research and Public Health. 2022;19(10):6270.

7. Chen TT, Hsieh TL, Chen ML, Tseng WT, Hung CF, Chen CR. Animal-Assisted Therapy in Middle-Aged and Older Patients With Schizophrenia: A Randomized Controlled Trial. Frontiers in Psychiatry. 2021;12:713623.

8. Chu C, Liu C, Sun C, Lin J. The effect of animal-assisted activity on inpatients with schizophrenia. Journal of Psychosocial Nursing & Mental Health Services. 2009;47(12):42-8.

9. Friedmann E, Galik E, Thomas SA, Hall PS, Chung SY, McCune S. Evaluation of a pet-assisted living intervention for improving functional status in assisted living residents with mild to moderate cognitive impairment: A pilot study. American Journal of Alzheimer's Disease and other Dementias. 2015;30(3):276-89.

10. Fung S-c, Leung AS-m. Pilot study investigating the role of therapy dogs in facilitating social interaction among children with autism. Journal of Contemporary Psychotherapy: On the Cutting Edge of Modern Developments in Psychotherapy. 2014;44(4):253-62.

11. Hill J, Ziviani J, Driscoll C, Teoh AL, Chua JM, Cawdell-Smith J. Canine Assisted Occupational Therapy for Children on the Autism Spectrum: A Pilot Randomised Control Trial. Journal of Autism and Developmental Disorders. 2020;50(11):4106-20.

12. Majic T, Gutzmann H, Heinz A, Lang UE, Rapp MA. Animal-assisted therapy and agitation and depression in nursing home residents with dementia: A matched caseecontrol trial. American Journal of Geriatric Psychiatry. 2013;21(11):1052-9.

13. Meints K, Brelsford VL, Dimolareva M, Marechal L, Pennington K, Rowan E, et al. Can dogs reduce stress levels in school children? effects of dog-assisted interventions on salivary cortisol in children with and without special educational needs using randomized controlled trials. PLoS ONE. 2022;17(6 June):e0269333.

14. Menna LF, Santaniello A, Gerardi F, Sansone M, Di Maggio A, Di Palma A, et al. Efficacy of animal‐assisted therapy adapted to reality orientation therapy: measurement of salivary cortisol. Psychogeriatrics. 2019;19(5):510-2.

15. Nieforth LO, Guerin NA, Stehli A, Schuck S, Yi K, O'Haire ME. Observation of human-animal interaction for research (OHAIRE) behavior coding in a randomized control trial of children with attention-deficit hyperactivity disorder (ADHD) and a canine-assisted intervention. Frontiers in Psychiatry. 2024;15.

16. Olsen C, Pedersen I, Bergland A, Enders-Slegers MJ, Ihlebak C. Effect of animal-assisted activity on balance and quality of life in home-dwelling persons with dementia. Geriatric nursing (New York, NY). 2016;37(4):284‐91.

17. Olsen C, Pedersen I, Bergland A, Enders-Slegers MJ, Patil G, Ihlebaek C. Effect of animal-assisted interventions on depression, agitation and quality of life in nursing home residents suffering from cognitive impairment or dementia: a cluster randomized controlled trial. International Journal of Geriatric Psychiatry. 2016;31(12):1312-21.

18. Parra EV, Garre JMH, Perez PE. Benefits of dog-assisted therapy in patients with dementia residing in aged care centers in Spain. International Journal of Environmental Research and Public Health. 2021;18(4):1-12.

19. Parra E, Manuel Hernández Garre J, Echevarría Pérez P. Impact of Dog-Assisted Therapy for Institutionalized Patients With Dementia: A Controlled Clinical Trial. Alternative Therapies in Health & Medicine. 2022;28(1):26-31.

20. Schuck SEB, Emmerson NA, Fine AH, Lakes KD. Canine-assisted therapy for children with ADHD: Preliminary findings from the Positive Assertive Cooperative Kids study. Journal of Attention Disorders. 2015;19(2):125-37.

21. Schuck SE, Emmerson NA, Abdullah MM, Fine AH, Stehli A, Lakes KD. A Randomized Controlled Trial of Traditional Psychosocial and Canine-Assisted Interventions for Children with ADHD: CABI International; 2018. 16 p.

22. Schuck SEB, Johnson HL, Abdullah MM, Stehli A, Fine AH, Lakes KD. The Role of Animal Assisted Intervention on Improving Self-Esteem in Children With Attention Deficit/Hyperactivity Disorder. FRONTIERS IN PEDIATRICS. 2018;6.

23. Scorzato I, Zaninotto L, Romano M, Menardi C, Cavedon L, Pegoraro A, et al. Effects of Dog-Assisted Therapy on Communication and Basic Social Skills of Adults With Intellectual Disabilities: A Pilot Study. Intellectual and developmental disabilities. 2017;55(3):125-39.

24. Shih CA, Yang MH. Effect of Animal-Assisted Therapy (AAT) on Social Interaction and Quality of Life in Patients with Schizophrenia during the COVID-19 Pandemic: An Experimental Study. Asian Nurs Res (Korean Soc Nurs Sci). 2023;17(1):37-43.

25. Stefanini MC, Martino A, Bacci B, Tani F. The effect of animal-assisted therapy on emotional and behavioral symptoms in children and adolescents hospitalized for acute mental disorders. European Journal of Integrative Medicine. 2016;8(2):81-8.

26. Stefanini MC, Martino A, Allori P, Galeotti F, Tani F. The use of Animal-Assisted Therapy in adolescents with acute mental disorders: A randomized controlled study. Complement Ther Clin Pract. 2015;21(1):42-6.

27. Travers C, Perkins J, Rand J, Bartlett H, Morton J. An evaluation of dog-assisted therapy for residents of aged care facilities with dementia. Anthrozoos. 2013;26(2):213-25.

28. Vidal R, Vidal L, Ristol F, Domenec E, Segu M, Vico C, et al. Dog-assisted therapy for children and adolescents with fetal alcohol spectrum disorders a randomized controlled pilot study. Frontiers in Psychology. 2020;11.

29. Vidal R, Vidal L, Lugo J, Ristol F, Domenec E, Casas T, et al. Dog-Assisted Therapy vs Relaxation for Children and Adolescents with Fetal Alcohol Spectrum Disorder: A Randomized Controlled Study. Journal of Autism and Developmental Disorders. 2023.

30. Villalta-Gil V, Roca M, Gonzalez N, Domenec E, Cuca, Escanilla A, et al. Dog-assisted therapy in the treatment of chronic schizophrenia inpatients. Anthrozoos. 2009;22(2):149-59.

31. Wijker C, Kupper N, Leontjevas R, Spek A, Enders-Slegers MJ. The effects of Animal Assisted Therapy on autonomic and endocrine activity in adults with autism spectrum disorder: A randomized controlled trial. General Hospital Psychiatry. 2021;72:36-44.

32. Wijker C, Leontjevas R, Spek A, Enders-Slegers MJ. Effects of Dog Assisted Therapy for Adults with Autism Spectrum Disorder: An Exploratory Randomized Controlled Trial. Journal of Autism and Developmental Disorders. 2020;50(6):2153-63.

33. Wolynczyk-Gmaj D, Ziolkowska A, Rogala P, Scigala D, Bryla L, Gmaj B, et al. Can dog-assisted intervention decrease anxiety level and autonomic agitation in patients with anxiety disorders? Journal of Clinical Medicine. 2021;10(21):5171.
