# Supplementary material for: Dog-assisted interventions for children and adults with mental health or neurodevelopmental conditions: systematic review
Source: Br J Psychiatry. 2025 Apr 14;228(2):150–63. doi: 10.1192/bjp.2025.8 (PMC7617605; doi:10.1192/bjp.2025.8)
Supplement: Shoesmith et al. supplementary material 4 — Shoesmith et al. supplementary material [file S000712502500008Xsup004.docx]

| Supplementary Material 4. Study characteristics | | | | | | | | |
| --- | --- | --- | --- | --- | --- | --- | --- | --- |
| Author/  Year &  Study Aim | **Country/**  **Setting** | **Participants (n)** | **Diagnosis/**  **diagnostic criteria** | **Intervention** | **Control** | **Follow-up** | **Outcome measures** | **Main findings reported by study authors** |
| Mental health conditions | | | | | | | | |
| Allen et al. (2021)  (71)  To explore whether the integration of dogs into CBT is feasible and beneficial for children with PTSD compared to standard treatment. | USA,  Outpatient treatment clinic | Children (ages 6-17); n = 33 (17 intervention; 16 control) | PTSD; PTSD Reaction Index for DSM-V | Trauma-focused CBT plus dog-assisted therapy | Trauma-focused CBT | None (post-intervention only) | Service satisfaction scale (SSS); UCLA PTSD reaction index; Strengths and difficulties questionnaire (SDQ); Moods and feelings questionnaire (MFQ); Screen for child anxiety related disorders (SCARED) | No significant improvements were found for self-reported PTSD symptom severity in the intervention group (pre-M = 39.5, SD = 18.44; post-M = 38.38, SD = 12.74). Significant improvements were reported in the control group (pre-M = 44.63, SD = 12.38; post-M = 28.09, SD = 24.09). The difference between groups was significant.  Significant improvements in caregiver-reported PTSD symptom severity in the intervention group (pre-M = 52.29, SD = 12.25; post-M = 35.85, SD = 14.81) and the control group (pre-M = 46.25, SD = 11.34; post-M = 22.64; SD = 16.43). |
| Calvo et al. (2016)  (50)  To assess the effect of DAI as an adjunct to conventional psychosocial rehabilitation for people with schizophrenia. | Spain, Psychiatric hospital | Adults; n = 24 (16 intervention; 8 control) | Schizophrenia; DSM-IV-TR | Dog-assisted therapy | Activity from functional programme based on therapist’s criteria (e.g., art therapy, group sports) | None (post-intervention only) | Positive and Negative Syndrome Scale (PANSS); EuroQol (EQ-5D) | Both groups showed significant improvements in positive symptomatology indicated by differences in pre- and post-treatment scores (intervention M = 5.28, SD = 4.78; control M = 7.87, SD = 4.29), and overall symptomology (intervention M = 10.00, SD = 8.70; control M = 12.63, SD = 13.57). Only the intervention group showed a significant improvement in negative symptomatology (intervention M = 5.64, SD = 8.19 vs. control M = 1.87, SD = 3.44). |
| Chen et al. (2021)  (47)  To assess the efficacy of a DAI for participants with schizophrenia in a clinical setting. | Taiwan, Psychiatric rehabilitation ward and day care ward | Adults over 40 years; n = 40 (20 intervention; 20 control) | Schizophrenia; DSM-V | Dog-assisted therapy | Non-animal related nursing intervention and occupational therapy of usual treatment programmes | None (post-intervention only) | PANSS; Chinese Happiness Inventory (CHI); Depression, Anxiety Stress Scales-21 (DASS-21) | Significant improvements in the PANSS from pre-intervention (median = 11.5) to post-intervention (median = 11.0) in the intervention group compared to no change in the control group (pre-intervention median = 11.5; post-intervention median = 11.5). |
| Chen et al. (2022)  (14)  To assess the efficacy of a DAI for participants with schizophrenia in a clinical setting. | Taiwan,  Psychiatric rehabilitation ward and day care ward | Adults over 40 years; n = 40 (20 intervention; 20 control) | Schizophrenia; DSM-V | Dog-assisted therapy | Non-animal related nursing intervention and occupational therapy of usual treatment programmes | None (post-intervention only) | Montreal Cognitive Assessment (MoCA); Chair Stand test (CST); Timed Up and Go (TUG); 5-meter walk test (5MWT); Assessment of Communication and Interaction Skills (ACIS) | Significant improvements in social skills from pre-intervention (median = 65.0, IQR = 10.50) to post-intervention (median = 71.50, IQR = 6.0) in the intervention group. Post-intervention social skills in the intervention group significantly improved compared to the control group (median = 65.0, IQR = 12.50). |
| Chu et al. (2009)  (68)  To explore the effects of a DAI on self-esteem, activities of daily living, and other psycho-physiological aspects among inpatients with schizophrenia. | Taiwan, Psychiatric ward | Adults up to age of 60; n = 30 (15 intervention; 15 control) | Schizophrenia; diagnostic criteria not specified | Dog-assisted activities | Treatment as usual | 1 week | Rosenberg Self-Esteem Scale; extent of social support; increases and decreases in adverse psychiatric symptoms (specific measures not specified) | Significant improvements in self-esteem from baseline (M = 9.68) to follow-up (M = 15.71) in the intervention group compared to the control group (baseline, M = 9.48; follow-up, M = 9.29). Significant improvements in positive symptoms from baseline (M = 14.88) to follow-up (8.46) in the intervention group compared to the control group (baseline, M = 15.85; follow-up M = 16.54). |
| Shih et al. (2023)  (67)  To evaluate the effectiveness of a DAI in improving social interactions and quality of life in participants with schizophrenia during the Covid-19 pandemic. | Taiwan,  Psychiatric rehabilitation | Adults; n = 90 (45 intervention; 45 control) | Schizophrenia; DSM-V | Dog-assisted therapy | Discussion groups with short films about animals | 3 months | Mental Health-Social Functioning scale; Social Adaptive Function Scale; Taiwanese version of the WHOQOL-BREF | Significant improvements in mental health and social functioning scores from baseline (M = 50.56) to post-intervention (M = 52.80) in the intervention group, but scores at 3-month follow-up declined (M = 46.07). Significant improves in quality of life from baseline (M = 79.33) to 3-month follow-up (M = 86.64) in the intervention group. There was a significant difference of the level of increase in quality of life scores from post-intervention to 3-month follow-up compared with the control group. |
| Stefanini et al. (2015)  (66)  To compare the effects of a DAI with a standard treatment protocol in children and adolescents admitted to a psychiatric hospital. | Italy, Psychiatric hospital | Children and adolescents; n = 34 (17 intervention; 17 control) | Any acute psychiatric diagnosis; ICD-9 | Dog-assisted therapy | Standard therapeutic protocol | 3 months | Children Global Assessment Scale (C-GAS); observations | Significant changes in all observational scales from baseline to follow-up in the intervention group: participants showed higher participation (pre-M = 2.27, SD = 0.49; follow-up M = 3.21, SD = 0.60), more interaction with their animal (pre-M = 2.38, SD = 0.52; follow-up M = 3.19, SD = 0.43), and more socialised behaviour with adults (pre-M = 1.73, SD = 0.72; follow-up M = 3.17; SD = 0.72). |
| Stefanini et al. (2016)  (20)  To examine the effects of a DAI on behavioural and emotional symptoms reported by children and adolescents admitted to a psychiatric hospital. | Italy, Psychiatric hospital | Children and adolescents; n = 40 (20 intervention; 20 control) | Severe psychiatric diagnosis; ICD-9 | Dog-assisted therapy | Standard therapeutic protocol | 3 months | Global functioning; Youth Self-report; observational data | Significant improvements were found for global functioning in the intervention group (pre M = 39.75, SD = 6.88; post M = 48.65, SD = 6.87), but not in the control group (pre M = 42.15, SD = 6.04; post M = 48.65, SD = 6.87). |
| Villalta-Gil et al. (2009)  (51)  To assess the effectiveness of including a therapy dog in an intervention programme delivered to participants with schizophrenia. | Spain, Hospital | Adults; n = 21 (12 intervention; 9 control) | Schizophrenia; DSM-IV | Dog-assisted therapy | Same intervention without presence of dog | None (post-intervention only) | PANSS; Living Skills Profile (LSP); Brief WHO Quality of Life Assessment-Spanish Version (WHOQOL-BREF); Satisfaction with Treatment Questionnaire (STQ) | Significant improvements were found in the intervention group for social contact (pre-M = 13.67, SD = 2.67; post-M = 18.00, SD = 4.4); PANSS score (pre-M = 88.25, SD = 12.17; post-M = 73.64, SD = 18.69), and quality of life related with social relationships (pre-M = 2.08, SD = 0.79; post-M = 2.85, SD = 0.56), but no significant differences were found between the intervention and control group. |
| Wolynczyk-Gmaj et al. (2021)  (52)  To investigate whether interactions with a dog can reduce anxiety symptoms in participants with anxiety or mixed-depressive anxiety disorders. | Poland, Hospital | Adults up to 60 years; n = 51 (25 intervention; 26 control) | Any anxiety or mixed depressive-anxiety disorder; ICD-10 | Dog-assisted activity | Walk with medical student or doctor rather than dog and handler | None (post-intervention only) | Beck Depression Inventory (BDI): Ford Insomnia Response to Stress Test (FIRST; The Brief Symptom Inventory (BSI-18); The Visual Analogue Scale of level of fear; the State-Trait Anxiety Inventory (STAI); The Visual Analogue Scale of level of satisfaction with the intervention. | Significant improvements were found for state anxiety in the intervention group (pre-M = 46.30, SD = 9.63; post-M = 34.35, SD = 6.88) compared to the control group (pre-M = 47.24, SD = 11.01; post-M = 40.94, SD = 8.59).  Significant improvements were found in the intervention group for anxiety as a trait (pre-M = 53.90, SD = 7.78; post-M = 48.90, SD = 7.16) and fear (pre-M = 2.57, SD = 2.29; post-M = 1.05, SD = 0.97). |
| Neurodevelopmental conditions | | | | | | | | |
| Fung et al. (2014)  (53)  To explore the effectiveness of therapy dogs to facilitate the social interaction of children with autism in a play context. | Hong Kong, School | Children (ages 6-10); n = 10 (5 intervention; 5 control) | Autism Spectrum Condition; DSM-IV | Dog-assisted play therapy | Play session with baby doll | None (post-intervention only) | Natural occurrence of child's social behaviour was measured based on observations | Significant increase in verbal social behaviour from pre- to post-intervention in the intervention group (*z* = -2.02, *p* = 0.43), compared to no significant difference in the control group (*z* = -1.48, *p* = .138). |
| Hill et al. (2020)  (19)  To explore the impact of incorporating a therapy dog into occupational therapy sessions on the on-task behaviours and goal attainment of children with autism when compared to usual care occupational therapy. | Australia,  Community (information and support service for individuals with ASC) | Children (ages 4-7); n = 22 (11 intervention; 11 control) | Autism Spectrum Condition; diagnostic criteria not specified | Dog-assisted occupational therapy | Usual care occupational therapy sessions | None (post-intervention only) | Observations to record ‘on-task behaviour’; Canadian Occupational Performance Measure to assess goal attainment | Difference in percentage time of on-task behaviour between the intervention (median = 92.60%) and control group (median = 90.03%) showed an increase in on-task behaviour of +2.6% within the intervention group when compared to the control group, but this was not significant. |
| Meints et al. (2022; study 2)  (54)  To explore the effects of a DAI on salivary cortisol levels in children in special educational needs schools. | UK,  Special educational needs school | Children; n = 44 | Autism Spectrum Condition; Attention Deficit Hyperactivity Disorder; Down Syndrome; other learning difficulties | Dog-assisted activities | Relaxation intervention or no treatment control group | None (post-intervention only) | Cortisol collection | No significant changes in cortisol levels for the intervention group (pre-M = .1381 μg/dL, SD = .06; post-M = .1158 μg/dL, SD = .04); or no treatment control group (pre-M = .1486 μg/dL, SD = .05; post- M = = .1486 μg/dL, SD = .06). |
| Nieforth et al. (2024)  (49)  To report on the behaviour coding of video data collected as part of the Schuck et al. 2018 RCT and explore whether the presence of a dog would lead to an increase in social behaviours. | USA, Community | Children (ages 7-9); n = 35 (18 intervention, 17 control) | ADHD; K-SADS-PL (interview with coding criteria to guidelines of DSM-IV-TR) | Dog-assisted therapy based on cognitive-behavioural strategies and social skills training | Same curriculum but used toy dogs | 6 weeks | OHAIRE coding system: summary scores were created for the following domains: Animal Social Interaction and Human Social Interaction (further separated into Human-Adult Social Interaction and Human-Peer Social Interaction). | No significant differences in how much children interacted with the live therapy dogs vs. control toy dogs. Children showed greater increases over time in human-directed social interactions in the presence of live dogs vs. toy dogs (*p* = 0.20). |
| Schuck et al. (2015)  (13)  To explore whether a DAI leads to improvements in prosocial skills and problematic behaviours in children with ADHD. | USA, Community | Children (ages 7-9); n = 24 (12 intervention; 12 control) | ADHD; K-SADS-PL (interview with coding criteria to guidelines of DSM-IV-TR) | Dog-assisted therapy based on cognitive-behavioural strategies and social skills training | Same curriculum but used toy dogs | 6 weeks | ADHD-Rating-Scale-Fourth Edition, Home, and School Version (ADHD-RS-IV); Social Skills Improvement System-Rating Scales, Parent Form (SSIS-RS); Social Competence Inventory (SCI) | Significant improvements found for social skills in both intervention (pre-M = 75.00, SE = 8.31; post-M = 92.00, SE = 13.88) and control groups (pre-M = 76.00, SE = 10.90; post-M = 86.42, SE = 1.94).  Significant improvements found for problem behaviours in both intervention (pre-M = 129.17, SE = 11.59; post-M = 112.67, SE = 14.72) and control groups (pre-M = 127.67, SE = 9.21; post-M = 115.33, SE = 9.67).  The severity of ADHD symptoms declined in both groups but participants in the intervention group exhibited greater reductions in the severity of ADHD symptoms (M at week 2 = 28.17, SE = 6.89; M at week 10 = 19.00, SE = 7.34). |
| Schuck et al. (2018a)  (48)  To explore whether a DAI is a feasible approach to improve self-esteem among children with ADHD. | USA, Community | Children (ages 7-9); n = 81 (41 intervention; 40 control) | ADHD; K-SADS-PL (interview with coding criteria to guidelines of DSM-IV-TR) | Dog-assisted therapy based on cognitive-behavioural strategies and social skills training | Same curriculum but used toy dogs | 6 weeks | The Self-Perception Profile for Children (SPSS); Social Skills Improvement System-Parent Form (SSIS-P); ADHD-RS-IV | Self-reported scores of behavioural conduct and social competence were significantly higher post-intervention than pre-intervention in the intervention group (z = 2.320, p = .021, z = 2.631, p = .008, and z = 2.541, p = .011, respectively). Pre/post differences were not found for those in the control group. |
| Schuck et al. (2018b)  (15)  To evaluate the efficacy of the intervention and control group for improving ADHD symptoms and social outcomes. | USA,  Community | Children (ages 7-9); n = 81 (41 intervention; 40 control) | ADHD; K-SADS-PL (interview with coding criteria to guidelines of DSM-IV-TR) | Dog-assisted therapy based on cognitive-behavioural strategies and social skills training | Same curriculum but used toy dogs | 6 weeks | ADHD-RS-IV; SSIS-P; The Social Competence Inventory (SCI) | Significant improvements were found for ADHD symptom severity in the intervention group from week 2 (M = 32.32, SD = 8.62) compared to immediately post-intervention (M = 20.86, SD = 7.66).  Significant improvements within the intervention group were found for social skills (baseline M = 79.54, SD = 11.09; follow-up M = 91.00, SD = 9.39); problem behaviours (baseline M = 125.98, SD = 14.39; follow-up M = 109.37, SD = 12.43), and prosocial orientation (baseline M = 3.02, SD = 0.58; follow-up M = 3.42, SD = 0.51). |
| Scorzato et al. (2017)  (55)  To determine the effects of a DAI on the behaviour, communication and social skills of adults with intellectual disabilities. | Italy, Community centres | Adults; n = 39 (21 intervention; 18 control) | Intellectual disability; ICD-10 | Dog-assisted therapy | Comparable activities but with no dog | None (post-intervention only) | International Classification of Functioning, Disability and Health (ICF); Behavioural Assessment Battery (BAB); Learning Accomplishment Profile (LAP) | Significant improvements within the intervention group were found for attention to movement (baseline M = 6.5; follow-up M = 15.5), visuomotor coordination (baseline M = 10.48; follow-up M = 14.67), exploratory play (baseline M = 15.55; follow-up M = 24.12), and motor imitation (baseline M = 18.71; follow-up M = 25.71).  Significant improvements were found for social skills in the intervention group (baseline M = 57.4; follow-up M = 67.1) but not in the control group (baseline M = 53.5; follow-up M = 50.0). |
| Vidal et al. (2020)  (72)  To evaluate the efficacy of a DAI in children and adolescents with FASD in relation to its effects on social skills, internalised and externalised symptomatology and on severity of FASD symptoms. | Spain, Hospital | Children and adolescents; n = 33 (17 intervention; 16 control) | Fetal alcohol spectrum disorder (FASC); established by senior psychiatrists | Dog-assisted therapy | Treatment as usual | 1 week | Child Behaviour Checklist (CBCL of Achenbach) parent version; Social Skills Improvement System-Parent Form (SSIS-P); Clinical Global Impression Scale for Severity (CGI-S) | Significant improvements were found for social skills in the intervention group (baseline M = 73.25, SD = 17.08; follow-up M = 81.56, SD = 16.58) compared to the control group (baseline M = 69.31, SD = 13.86; follow-up M = 69.46, SD = 13.51).  Significant improvements were found for FASC severity in the intervention group (baseline M = 3.76, SD = 0.43; follow-up M = 3.18, SD = 0.39) compared to the control group (baseline M = 3.44, SD = 0.5; follow-up M = 3.44, SD = 0.61). |
| Vidal et al. (2023)  (56)  To evaluate the efficacy of a DAI in children and adolescents with FASD in relation to its effects on social skills, internalised and externalised symptomatology, severity of FASD symptoms, and quality of life. | Spain,  Hospital | Children and adolescents; n = 71 (38 intervention; 33 control) | Fetal alcohol spectrum disorder (FASC); established by senior psychiatrists | Dog-assisted therapy | Relaxation intervention | None (post-intervention only) | Social Skills Improvement System-Parent Form (SSIS-P); Child Behaviour Checklist (CBCL of Achenbach) parent version; KidScreen 27 Parent version; Clinical Global Impression Scale for Severity (CGI-S) | Significant improvements were found for social skills in the intervention group (baseline M = 68.42, SD = 18.46; post-intervention M = 73.71, SD = 17.51) compared to the control group (baseline M = 71.56, SD = 19.34; post-intervention M = 73.75, SD = 20.46).  Significant improvements were found for FASC severity in the intervention group (baseline M = 4.16, SD = 0.91; post-intervention M = 3.55, SD = 0.76), but these were not significantly different from the control group. |
| Wijker et al. (2020)  (21)  To explore the effects of a DAI in adults with ASD, focusing on self-perceived stress, social responsiveness, and psychological symptoms (e.g., depression and anxiety). | Netherlands, Mental health care organisation | Adults up to 60 years; n = 53 (27 intervention; 26 control) | Autism Spectrum Condition; ADI-R and DSM-V | Dog-assisted therapy | Wait list control group | 10 weeks | Perceived Stress Scale (PSS); Symptom Checklist-90 revised (SCL-90-R); Social Responsiveness Scale for Adults (SRS-A); Rosenberg Self-Esteem Scale | Significant improvements were found for social skills in the intervention group, but not in the control group. No significant improvements were found for stress, symptoms, or self-esteem. |
| Wijker et al. (2021)  (23)  To examine the psychophysiological responses of adults with ASC to a DAI session. | Netherlands, Mental health care organisation | Adults up to 60 years; n = 53 (27 intervention; 26 control) | Autism Spectrum Condition; ADI-R and DSM-V | Dog-assisted therapy | Wait list control group | 10 weeks | Cortisol and alpha-amylase collection | Significant decreases were found for cortisol at the end of an intervention session (M = 8.74, SD = 3.14) compared to the start of the session (M = 10.04, SD = 3.14). No significant differences were found for cortisol or alpha-amylase levels pre- and post-intervention or compared to the control group. |
| Dementia | | | | | | | | |
| Baek et al. (2020)  (57)  To investigate the psychological and behavioural effects of a DAI on cognitive function, emotional state, problematic behaviours and activities of daily living among adults with dementia. | South Korea, Hospital | Older adults (65 years+); n = 36 (18 intervention; 18 control) | Dementia; Korean version of the MMSE | Dog-assisted therapy | ‘Non-equivalent control group’ (not specified) | None (post-intervention only) | Korean version of the MMSE; Faces Rating Scale; Korean version of the Cornell Scale for Depression in Dementia (CSDD); Barthel Index; Problem behaviour assessment tool | No significant group differences post-intervention in mood status (intervention M = 1.57, SE = 0.17 vs. control M = 2.57, SE = 0.17); depressive symptoms (intervention M = 6.35, SE = 4.06 vs. control M = 10.71, SE = 1.27); or problematic behaviours (intervention M = 7.07, SE = 0.96 vs. control M = 8.64, SE = 0.89). |
| Bono et al. (2015)  (58)  To examine the clinical effect of a DAI to non-hospitalised participants with dementia. | Italy,  Memory clinic | Older adults (65 years+); n = 32 (16 intervention; 16 control) | Minor to mild Alzheimer’s Disease; diagnosed as outpatients | Dog-assisted therapy | No information provided | None (post-intervention only) | Barthel Index; Alzheimer Disease Assessment scale (ADAS); CSDD | Significant improvements in depressive symptoms at follow-up in the intervention group (M = 8.1, SE = 2.3) compared to the control group (M = 4.4, SE = 1.4). |
| Briones et al. (2021)  (18)  To assess the effectiveness of a DAI to enhance quality of life in participants with dementia in a public care home. | Spain, Residential aged care facility | Older adults (65 years+); n = 32 (16 intervention; 18 control) | Dementia; MEC-10 | Dog-assisted therapy | Treatment as usual | None (post-intervention only) | Quality of Life in Alzheimer’s Disease (QoL-AD); Barthel Index; MEC-30 | Both groups showed an improvement in quality of life from pre-intervention (intervention M = 30.85, SD = 1.50; control M = 29.00, SD = 1.6) to post-intervention (intervention M = 32.46, SD = 1.27; control M = 31.50, SD = 1.40) but the improvement was only significant in the control group. |
| Friedmann et al. (2015)  (59)  To evaluate the use of structured activities with a dog to prevent deterioration of physical, emotional and behavioural function in participants with dementia. | USA, Assisted living facilities | Adults over 55 years; n = 40 (22 intervention; 18 control) | Dementia; MMSE | Pet-assisted living (PAL) intervention (dog-assisted activities) | Reminiscing programme | None (post-intervention only) | Barthel Index; CSDD; Zimmerman's short version of Apathy Evaluation Scale (AES); Cohen-Mansfield Agitation Inventory (CMAI) | Significant improvements in depressive symptoms from pre-intervention (M = 8.91, SE = 1.21) to post-intervention (M = 5.21, SE = 0.77) in the intervention group compared to the control (baseline M = 8.28, SE = 1.30; follow-up M = 8.67, SE = 1.51). No significant group differences were found for apathy or agitation. |
| Majic et al. (2013)  (60)  To investigate the efficacy of a DAI on symptoms of agitation, aggression and depression in participants with dementia. | Germany, Nursing homes | Older adults; n = 65 (intervention 30; control 35) | Moderate to severe dementia; MMSE and DSM-IV | Dog-assisted therapy | Treatment as usual | None (post-intervention only) | CMAI; Dementia Mood Assessment Scale (DMAS) | No significant differences over time in the intervention group for mood (pre-M = 26.85, SD = 16.91; post-M = 21.59, SD = 16.36) or agitation (pre-M = 46.78, SD = 16.89; post-M = 45.96, SD = 15.87). No significant differences were found between the intervention and control group for mood or agitation. |
| Menna et al. (2019)  (61)  To evaluate whether a DAI adapted to formal Reality Orientation Therapy could modify salivary cortisol levels in participants with dementia. | Italy,  Adult day care centre | Older adults; n = 22 (11 intervention; 11 control) | Mild to moderate Alzheimer’s Disease; diagnostic criteria not specified | Dog-assisted therapy using Reality Orientation Therapy protocol | Activities based on Reality Orientation Therapy protocol | None (post-intervention only) | MMSE; Geriatric Depression Scale (GDS); cortisol collection | Depression levels significantly decreased in the intervention group (pre-M = 14.3, SD = 2.6; post-M = 12.3, SD = 2.37), but significantly increased in the control group (pre-M = 13.6, SD = 4.3; post-M = 14.5, SD = 4.5). |
| Olsen et al. (2016a)  (64)  To examine the effectiveness of a DAI on quality of life in participants with dementia. | Norway,  Day centres | Older adults (65 years+); n = 80 (42 intervention; 38 control) | Mild to severe dementia; MMSE | Dog-assisted activities | Treatment as usual | 3 months | Quality of Life in late-stage dementia (QUALID) | No significant improvements for quality of life were reported in the intervention group (baseline M = 15.89; follow-up M = 16.65) or the control group (baseline M = 15.94; follow-up M = 15.23). |
| Olsen et al. (2016b)  (65)  To examine the effects of a DAI on depression, agitation, and quality of life in participants with dementia. | Norway, Nursing homes | Older adults (65 years+); n = 58 (28 intervention; 30 control) | Dementia; MMSE | Dog-assisted activities | Treatment as usual | 3 months | Behavioural Activity Rating Scale (BARS); CSDD; QUALID; Clinical Dementia Rating Scale | Depression levels significantly improved in the intervention group (baseline M = 8.35, SD = 4.65; follow-up M = 7.41, SD = 5.01), compared to the control group (baseline M = 6.88, SD = 4.70; follow-up M = 9.58, SD = 6.61).  No significant differences in agitation were found between intervention (baseline M = 23.44, SD = 7.64; follow-up M 24.87, SD = 8.34) or control groups (baseline M = 23.19, SD = 11.39; follow-up M = 24.00, SD = 13.20). |
| Parra et al. (2021)  (62)  To evaluate the benefits that a DAI could provide for affective, behavioural, cognitive, and functional aspects of participants with dementia. | Spain,  Care centres | Older adults (65 years+); n = 371 (186 intervention; 185 control) | Dementia; MMSE | Dog-assisted therapy | Treatment as usual | None (post-intervention only) | MEC-30; Modified Barthel Index; CSDD; Neuropsychiatric Disorders Inventory (NPI) | Depression levels significantly improved in the intervention group (pre-M = 8.14, SD = 6.92; post-M = 4.33, SD = 5.4) and significantly worsened in the control group (pre-M = 8.14, SD = 6.91; post-M = 9.34, SD = 7.1).  Agitation levels significantly improved in the intervention group (pre-M = 10.67, SD = 19.2; post-M = 5.3, SD = 14.8), and significantly worsened in the control group (pre-M = 11.32, SD = 20.5; post-M = 12.9, SD = 22.2). |
| Parra et al. (2022)  (16)  To evaluate the benefits that a DAI could provide for emotional, behavioural, cognitive, and functional aspects of participants with dementia. | Spain, Nursing home | Older adults (65 years+); n = 21 (7 people with dementia in intervention; 7 people with dementia in control; 7 without dementia in ‘healthy group’) | Dementia; MMSE | Dog-assisted therapy | Treatment as usual | None (post-intervention only) | MMSE; Modified Barthel Index; The Yesavage Geriatric Depression Scale (GDS-VE); NPI | Significant differences found  between the intervention group and the control group in agitation and depression, with *p* < .037 and .011, respectively. |
| Travers et al. (2013)  (63)  To assess the effectiveness of a DAI on mood, quality of life, and psychosocial functioning in participants with dementia. | Australia,  Aged care facilities | Older adults; n = 67 (34 intervention; 33 control) | Mild to moderate dementia; resident’s medical record | Dog-assisted therapy | Human-therapist-only | None (post-intervention only) | Modified MMSE (MSE-3MS) QoL-AD; Medical Outcomes Study 36-item Short Form Health Survey v1 (SF-36); GDS; Multidimensional Observational Scale for Elderly Subjects (MOSES) | Significant improvements were found for quality of life in one facility post-intervention (M = 38.1, SD = 4.4) compared to the control group (M = 33.2, SD = 5.3) but were significantly lower in two other facilities (M, 34.0, SD = 7.2; M = 34.7, SD = 4.9, respectively) compared to their respective control groups (M = 38,9, SD = 5.9; M = 39.6, SD = 6.1, respectively). No significant group differences were found for other measures. |
| (PTSD) Post-traumatic stress disorder; (ADHD) attention deficit hyperactivity disorder; (DSM) Diagnostic and Statistical Manual of Mental Disorders; (ICD) International Statistical Classification of Diseases and Related Health Problems; (ADI-R) Autism Diagnostic Interview – Revised; (CBT) Cognitive-behavioural therapy; (OHAIRE) Observation of Human-Animal Interaction for Research Coding System; (MMSE) Mini mental state examination; (M) mean; (SD) standard deviation; (SE) standard error | | | | | | | | |
