# Supplementary material for: Dog-assisted interventions for children and adults with mental health or neurodevelopmental conditions: systematic review
Source: Br J Psychiatry. 2025 Apr 14;228(2):150–63. doi: 10.1192/bjp.2025.8 (PMC7617605; doi:10.1192/bjp.2025.8)
Supplement: Shoesmith et al. supplementary material 5 — Shoesmith et al. supplementary material [file S000712502500008Xsup005.docx]

**Supplementary Material 5.** Intervention characteristics

| Author/  Year | Content | Frequency/  duration | Group/  individual | Dog breed | Delivered by | Space |
| --- | --- | --- | --- | --- | --- | --- |
| Allen et al. (2021) | The first four sessions focused on providing psychoeducation, teaching relaxation and coping skills. Session 5 - 9 focused on the narrative of the trauma experience while reinforcing approach behaviours that facilitate effective coping. The final four sessions included the youth discussing the trauma narrative with the caregiver in a joined session. A dog was required to be in the room for all sessions where the youth were engaged in treatment, including the joined session with the caregiver. The manner in which the youth interacted with the dog was not dictated. The clinician provided cues for the dog to approach or move away in accordance with the youth's preferences. During situations where the youth appeared stressed, the clinician offered interaction with the dog as a coping skill alongside previously taught skills but did not otherwise introduce the dog into the activities of the sessions | 12 x 90 minute sessions | Individual sessions (with some joined session with caregiver) | 5 x Labrador retrievers | Clinicians trained to Trauma-focused CBT certification requirements and trained by handlers in understanding canine body language | Therapy room |
| Baek et al. (2020) | Sessions grouped into three stages (introductory, developmental, and final stages). Aims of the developmental stage were to enhance interaction, emotional stability, ADL, and cognitive function. Participants trimmed dogs’ fur, trained, and walked them, and talked to them about their feelings | 2 x 60 minute sessions/  weekly for 8 weeks | Group | Breed not specified | Animal mediation psychologist | Large space in hospital for both participant and therapy dog (99m^2^) |
| Bono et al. (2015) | Activities included cognitive stimulation (spatial-temporal orientation, mnemonic stimulation, dog ‘introduction’ to the participant, identification of the dog breed, learning basic commands, etc.); communication (learning the body language of the animal); motor activity strolling with the dog, petting the dog, throwing a small ball), and wellbeing and entertainment | Biweekly, 60 minute sessions for 8 months | Individual | 1 x Golden Retriever, 2 x Border Collies, 1 x Weimaraner | Animal trainer | Rooms at the Memory Clinic |
| Briones et al. (2021) | Content of the sessions were planned to achieve specific objectives. The technicians established the content of the sessions, which, responding to specific objectives, comprised activities focused on cognitive and motor functions, communication, recreational pursuits, and well-being | Weekly 50 minute sessions for 9 months | Group | 1 x Labrador retriever, 1 x golden retriever | Animal-assisted therapy technicians | Room used exclusively for intervention |
| Calvo et al. (2016) | Three types of sessions were involved: (1) sessions to develop the emotional bond between participants and dogs. The participants were taught to handle and take care of dogs correctly. In this type of session, concepts of animal welfare and responsible ownership were explained and practiced; (2) sessions involving walking the dogs. During the first half of the programme, the dogs were walked in a large natural park so patients could learn to walk the dogs in a calm and controlled manner. For the rest of the programme, participants walked the dogs in the city so they could experience dog walking in a social context; (3) sessions to train and play the dogs: patients learned to give instructions to the dogs and train them using positive reinforcement training techniques | 2 x weekly 60 minute sessions for 6 months | Group | Not specified | Animal-assisted therapy technicians | Various settings as sessions involved leaving the hospital to various locations (e.g., parks/city for dog walking). |
| Chen et al. (2021; 2022) | The primary goal was to improve negative symptoms and general psychopathology symptoms. Secondary goals were aimed at improving positive symptoms and patient wellbeing. The dog approached the participants in turn, and each participant walked the dog around the classroom. Each session was carried out with a similar structure: 15-min warm up, 45-min therapeutic activities, 5 min feedback. In the warm-up, the therapist greeted each participant, introduced the dog, reviewed the contents of the last session and oriented participants to the activities. There were four types of therapeutic activities: activity for positive emotion (e.g., touching the dog, massaging the dog, playing with the dog), social activity (e.g., cooperating in the games with each other and the dog), cognitive activity (e.g., training the dog), and physical activity (e.g., walking, handling, feeding, grooming the dog). Each activity was performed for three sessions with gradually increasing levels of difficulty. Therapists gave feedback on what the group did during the therapeutic activities, asked how they felt with the dog, and previewed the content of the next session | 60 minute sessions over a 12 week period | Group | 1 x Corgi, 1 x Labrador | Animal-assisted therapist, occupational therapist, and dog-handler pair | Conducted in a spacious and quiet classroom with the participants sat in a semi-circle |
| Chu et al. (2009) | A different activity was held each week. Session one: Introduction and getting acquainted with the dog; Session two: walk with the dogs; Session three: walk with the dogs over barriers; Session four: carry the dogs on a cart, touch the dogs, play with the dogs; Session five: throw and chase big and small balls and play with dogs; Session six: throw and fetch small balls and play with the dog; Session seven: raise the dogs attention/play with the dogs; Session eight: group discussion and exchange of feelings. Participants were encouraged to interact with the dogs and freely express their feelings and thoughts. The activities were not rigidly designed; rather, they allowed the patients and animals to interact freely. No specific goals or achievements were set before each visit | Weekly x 50 minute sessions over 2 months | Group | Not specified | Research investigator | Hospital garden but took place in the activity hall during inclement weather and during first 2 weeks to not distract participants |
| Friedmann et al. (2015) | Programme included activities designed to encourage maintenance of physical and emotional function: feeding the dog, brushing the dog’s teeth and hair, dressing the dog in a bandana (activities of daily living), throwing a ball, grooming the dog (range of motion), adjusting a collar, feeding the dog a treat, petting the dog (small motor skills) opening treats and giving the dog a treat (sequencing events), and talking to the dog (social skills) | 1 x 60-90 minute session/  biweekly for 12 weeks | Group | Cardigan Welsh Corgi | Nurse practitioner | Group living room |
| Fung et al. (2014) | The children could have physical contact with the dogs, including such activities as identifying their body parts and petting them. The children would also be guided to take care of them, for example, combing their hair and feeding them. Moreover, they would act as social partners of the children. The children would walk the dogs and throw balls to them | 20 minute sessions, 3 x weekly for 7 weeks | Individual | 2 x Golden retrievers | Trained animal-assisted therapist | All sessions conducted at the school in a multipurpose room with which the participants were already familiar |
| Hill et al. (2020) | All therapy sessions consisted of occupation focused, goal directed interventions. For example, if the goal was the development of a dynamic tripod grasp in preparation for starting school, the activity would involve colouring in cartoon bones to symbolise the number of treats the child would be able to give to the therapy dog | 1 x weekly 60 minute sessions for 9 weeks | 7 sessions with parent and child; 2 parent sessions for goal setting (child not present) | Labradoodle | Trained animal-assisted therapist | Sessions took place within an animal-assisted psychology practice, specifically suited to facilitation of therapy dogs working within the clinic |
| Majic et al. (2013) | In the initial session, the dog was presented to the participant, introducing the name of the dog and when the dog would be visiting. The sessions started with interaction between the therapy guide and the dog, the participant speaking to the dog, and then physical interaction such as stroking/petting the dog. The last 15 minutes of the sessions included spontaneous dynamic processes between dog and participants, allowing for free interaction between the two | 1 x 45-minute session, weekly for 10 weeks | Not specified | Border collies | Qualified dog handler | Not specified |
| Meints et al. (2022; study 2) | Each intervention began and ended with the child greeting the dog as advised by the dog handler and time for petting the dog if appropriate—this phase of active contact lasted roughly 5 minutes. The next and central part of the session was based on the dog with children learning facts about the dogs from the handler, watching the dog, talking about, and interacting with the dog. This was child-led and sessions varied in verbal content depending on questions children asked about the dog, and it lasted approximately 10 minutes. The last part of the session was ‘saying goodbye’, and again a chance to pet the dog. Any child worried about meeting the dog did not need to take part–but, if they wanted to, was purposely seated next to the researcher and the greeting process was child-led. | 8 x 20 minute sessions held twice a week for four weeks | Either individual or in small groups (up to 7 children) | 1 x Greek Hare-Hound, 2 x Cavalier King Charles Spaniel and Miniature Poodle crossbreed, 1 x Labrador and miniature Poodle crossbreed, 2 x German Short-Haired Pointers, 2 x Miniature Schnauzers, 3 x Labradors and 1 x Labrador crossbreed, 2 x Tibetan Mastiffs, 1 x Border Terrier, 1 x Scottish Terrier, 1 x Lurcher, 1 x Clumber Spaniel, 1 x Yorkshire Terrier, 1 x Pekingese, 1 x Smooth Collie, 1 x Cocker Spaniel and 1 x Golden Retriever. | Professional animal handler | Not specified |
| Menna et al. (2019) | Therapy with dog using Reality Orientation Therapy (ROT) – first 15 minutes involving introducing the dog, and the next 20 minutes included structured activity (e.g., play fetch, hiding the ball, caring for the dog). The last 10 minutes involved the same ending activity each time. There was a closing speech and washing of hands. The interactions with the animal were structured according to ROT, with some adaptations. | 1 x weekly session for 3 months | Group | Labrador retriever | Veterinarian | Not specified |
| Olsen et al. (2016a) | Various activities (e.g., petting the dog, brushing the dog, feeding the dog, throwing a toy for the dog to fetch). Idea of the sessions was that physical functions would be enhanced by doing different physical tasks (e.g., bending down, reaching out, lifting arms, throwing balls). It was also assumed cognition, self-efficacy and fine motor skills may be enhanced if participants were to give the dog commands and reward the dog. All sessions followed a main protocol but were individually tailored to each participant based on the care workers’ knowledge of the individual. | 1 x 30 minute session/  twice a week for 12 weeks | Groups of 3 – 7 | Not specified | Qualified dog handler | Randomly seated in a half circle in a room |
| Olsen et al. (2016b) | Various activities (e.g., petting the dog, brushing the dog, feeding the dog, throwing a toy for the dog to fetch). Idea of the sessions was that physical functions would be enhanced by doing different physical tasks (e.g., bending down, reaching out, lifting arms, throwing balls). It was also assumed cognition, self-efficacy and fine motor skills may be enhanced if participants were to give the dog commands and reward the dog. All sessions followed a main protocol but were individually tailored to each participant based on the care workers’ knowledge of the individual. | 1 x 30 minute session/  twice a week for 12 weeks | Groups of 3 – 7 | Not specified | Qualified dog handler | Randomly seated in a half circle in a room |
| Parra et al. (2021) | First sessions introduced participants and dog, and the following sessions consisted of working on the area under study through direct interaction with the dog and a final phase of farewell. The strategies used according to the objectives to be worked on were: (1) affective area - caressing and brushing the dog; (2) behavioural area – promotion of sustained attention to the dog and to the activity; (3) functional area – activities aimed at working motor skills, and (4) cognitive area – working on spatial and temporal orientation. | 1 x 45 minute session weekly for 8 months | Groups of 10 | Not specified | Dog-assisted therapy technician | Not specified |
| Parra et al. (2022) | The objectives for each session were set by the occupational therapist and the DAT technician based on participants’ needs. For large group sessions, activities included: (1) Perringo, a bingo game in which the dog has the numbers in a container on its harness’ chest panel; (2) snakes and ladders, a team game in which participants need to pass a series of tests with the dog until reaching the last box with the last test; (3) Simon Says, a game in which participants must imitate the positions adopted by the dog; and (4) You Say, a game in which participants must ask the dog to adopt a position depending on the colour shown by the dice. For small group sessions, content was designed to work on specific therapeutic objectives. Activities included: six sessions to establish a bond with the dog, during which the dog was presented as an animal and as an individual; participants received information about its needs, learning, methods of communication with the dog, and correct handling; (2) six sessions with different topics—animals, seasons of the year, numbers, words, and food—aimed primarily at stimulating the cognitive area; (3) six sessions aimed primarily at stimulating the functional area—fine and gross motor skills, walking, and balance and coordination; and (4) six sessions aimed primarily at socioemotional stimulation, recognising and expressing emotions, interaction with others, and participation. | 2 x 45 minute weekly sessions over a period of 6 months | Groups (large or small depending on session) | Not specified | Dog-assisted therapy technician and occupational therapist | Not specified |
| Scorzato et al. (2017) | The activities were structured according to a pattern of growing complexity and interaction (i.e., from simple observation with no interaction toward more complex group activities). The programme was designed to be as inclusive as possible, and the different activities were conceived to be simple and flexible in order to adapt to each participant's needs and abilities. | 30 minute 1 x weekly sessions for 20 sessions | Group of 3 | 2 x Bernese Mountain Dogs, 1 x Bergamasco Shepherd, 1 x French Bulldog | Professional dog handler | A room at the Pet Therapy Centre, about 42sqm and included tables, chairs and different toys |
| Schuck et al. (2015) | Children were supported in ‘training’ basic commands (come, sit, stay) with certified therapy dogs. The social skills curriculum used within the programme combines cognitive-behavioural and behavioural theories with behaviour modification techniques and social problem solving strategies to promote adaptive skill acquisition. The model, derived from research on programmes designed to teach social skills and friendship making skills has been adapted for children with ADHD and utilises a combination of didactic instruction, modelling, and role-play, while implementing a token economy, group and individual contingencies, and differential positive reinforcement of adaptive behaviours that are incompatible with problem behaviours. | 2 x weekly for 12 weeks (one weekday evening for 2 hours and one weekend day for 2.5 hours) | Group | Not specified | Professional dog handler | Not specified |
| Schuck et al. (2018a; 2018b); Nieforth et al. (2024) | Children were supported in ‘training’ basic commands (come, sit, stay) with certified therapy dogs. The social skills curriculum used within the programme combines cognitive-behavioural and behavioural theories with behaviour modification techniques and social problem solving strategies to promote adaptive skill acquisition. The model, derived from research on programmes designed to teach social skills and friendship making skills has been adapted for children with ADHD and utilises a combination of didactic instruction, modelling, and role-play, while implementing a token economy, group and individual contingencies, and differential positive reinforcement of adaptive behaviours that are incompatible with problem behaviours. | 2 x weekly for 12 weeks (one weekday evening for 2 hours and one weekend day for 2.5 hours) | Group | Not specified | Professional dog handler | Not specified |
| Shih et al. (2023) | The intervention was implemented through support groups to help participants develop their skills in  social interaction and emotional expression. Week 1 and 2 involved building relationships with peers and the dogs. Weeks 3 – 6 involved brief interactions with the dogs (e.g., instructing the dog to sit down, walking with the dog, feeding the dog). Weeks 7-12 involved deeper interactions with the dog (e.g., grooming and talking to the dog, going through tunnels with the dogs, interacting with the dogs without seeing the dogs, and engaging in creative activities about the dog). | Weekly sessions (60 minutes) for 12 weeks | Group | Not specified | Researchers and social workers, along with the participation of two animal-assisted therapists. | Reception hall |
| Stefanini et al. (2015) | Consisted of structured sessions in accordance with the individual therapeutic goals for each patient. This programme had four phases: 1) familiarisation with the animal and the handler; 2) individual intervention; 3) group activity; 4) discussion of the CAT experience. During the session, participants interacted with a dog and the handler. The repertoire of CAT intervention covers a wide range of activities and consists in play activities, physical contact, grooming, cleaning, basic obedience commands, walking, and agility routes | Weekly sessions (approx. 45 minutes) for 3 months | Both individual and group | Not specified | Qualified animal handler | Conducted in the hospital's garden or in an activity room when weather was bad. |
| Stefanini et al. (2016) | The CAT program had four phases: (1) familiarization with the animal and the handler: patients were already familiarized with their animal in two introductory sessions and an appropriate animal-patient pairing was established until the end of treatment; (2) individual intervention: the aim of the individual session was to gradually experiment the ways to interact with the animal; the relations with the animal were given priority; the patient spontaneously engaged in taking care of the animal and becoming familiar with non-verbal language; (3) group activity: in group sessions the objective was to test psychosocial functioning, the activity gave priority to a cooperative relationship with the other members of the group and to team work; (4) discussion of the CAT experience: the team looked at the video recordings for the purpose of compiling the observation forms and discussed the progress of the treatment. During each session the participant interacted with a dog and its handler in the following way: play activities, physical contact, grooming, cleaning, basic obedience commands, walking, and agility routes. | 10 sessions (45 minutes) for three months | Both (5 in group, 5 in individual) | Not specified | Staff members trained in dog-assisted therapy programmes | Conducted in the hospital's garden or in an activity room when weather was bad. |
| Travers et al. (2015) | Semi-structured session protocols were developed and implemented. Each session involved an introductory activity, general discussion, opportunity for participant to individually interact with the dog (playing, petting, feeding), and concluded by reading a short story to the group. Only one dog was present at a session, and the dogs were used in a rotating order to minimise stress to dogs | 2 x 40-50 minute sessions/  weekly (two consecutive days) for 11 weeks | Group of approx. 10 | 1 x miniature poodle, 1 x Staffordshire terrier, 1 x German shepherd | Therapist who was also a veterinarian and a registered psychiatric nurse | Private room to conduct the sessions in each facility |
| Vidal et al. (2020) | Sessions in the individual module were as follows: session 1, getting to know each other to determine the strengths and weaknesses of each patient; session 2, frustration tolerance and motivation; session 3, impulsivity management (self-control strategies, sequential thinking); session 4, emotional self-regulation (identifying emotional triggers and alarm signs); session 5, executive functions (planning, cause effect thinking); session 6, review of the contents. The last six group sessions focused on social skills (criticism management, communication and cooperation, adaptive behaviour, assertiveness training and empathy). Dogs facilitated the achievement of the therapeutic goals set by the psychologist who conducted the intervention. | 12 sessions approx. 45 minutes over 3 months | 6 individual; 6 group (3 – 4) | Not specified | 2 dog-assisted therapist professionals and psychologist | Not specified |
| Vidal et al. (2023) | Sessions in the individual module were as follows: session 1, getting to know each other to determine the strengths and weaknesses of each patient; session 2, frustration tolerance and motivation; session 3, impulsivity management (self-control strategies, sequential thinking); session 4, emotional self-regulation (identifying emotional triggers and alarm signs); session 5, executive functions (planning, cause effect thinking); session 6, review of the contents. The last six group sessions focused on social skills (criticism management, communication and cooperation, adaptive behaviour, assertiveness training and empathy). Dogs facilitated the achievement of the therapeutic goals set by the psychologist who conducted the intervention. | 1 x weekly sessions (45 minutes) for 16 weeks | 8 individual; 8 group (3-4) | Not specified | One technician specialising in dog-assisted therapy and a psychologist for individual group modules | Not specified |
| Villalta-Gil et al. (2009) | Intervention was based on Integrated Psychological Treatment (IPT). It is a group intervention structured programme with five subprogrammes: cognitive differentiation, social perception, verbal communication, social skills training, and interpersonal problem solving. In this study, it was partially modified to bring dogs into the therapy sessions. Sessions for the dog assisted group were designed so the handler interacted with the dog and the therapist, the therapist interacted with the patient and the handler, and patients interacted with the dog and the therapist. Sessions were also designed so materials used in the IPT (cards, sentences, etc.) were substituted by, or were referenced to, the dog. The dog had an active role within the sessions. | 2 x weekly sessions (45 minutes) for 25 sessions | Small groups (3-4) | Labrador | Trained psychologist | Not specified |
| Wijker et al. (2020; 2021) | The programme was developed by therapists and dog behavioural specialists from a Dutch service dog foundation and psychologists from a mental health care organisation who had a specialisation in Autism. The programme had a structured protocol, and a therapy dog was involved during all sessions. During the sessions, the therapists used a semi-structured therapy protocol. The aim of the intervention was to reduce stress and stress-related outcomes, such as depression and anxiety and to improve social and communication skills. | 10 weekly sessions (60 minutes) | Individual | 2 x Labradors, 4 x Labrador crossbreeds, 1 x golden retriever, 3 x golden retriever crossbreeds, 2 x poodles, 1 German Wirehaired Pointer | Therapists who had completed advanced courses in dog behaviour and welfare. | Therapy room |
| Wolynczyk-Gmaj et al. (2021) | The participant exited the building and was introduced to the dog’s handler and the assistance dog, who were already waiting in the surrounding green area. The handler was asked to focus the conversation on the dog and the participant’s experience with dogs in general and he could also show the skills of his dog, so the test conditions were comparable for all subjects. Physical contact with the dog (such as petting) was permitted, the participant was free to decide on the level of interaction. | One 15-20 minute session | Individual | German Shepherd | Professional animal handler | Garden outside the ward |
