# Supplementary material for: Dog-assisted interventions for children and adults with mental health or neurodevelopmental conditions: systematic review
Source: Br J Psychiatry. 2025 Apr 14;228(2):150–63. doi: 10.1192/bjp.2025.8 (PMC7617605; doi:10.1192/bjp.2025.8)

**Supplementary Material 6.** Risk of bias summary: review authors’ judgements about each risk of bias item for each included paper.


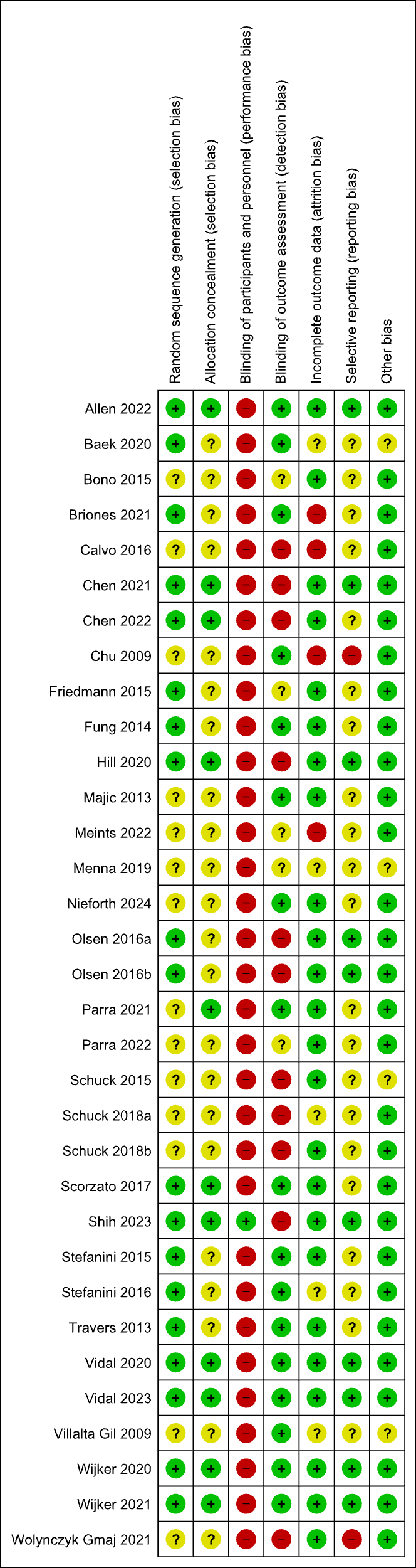

Supplement: Shoesmith et al. supplementary material 6 — Shoesmith et al. supplementary material [file S000712502500008Xsup006.docx]
