# Supplementary material for: Dog-assisted interventions for children and adults with mental health or neurodevelopmental conditions: systematic review
Source: Br J Psychiatry. 2025 Apr 14;228(2):150–63. doi: 10.1192/bjp.2025.8 (PMC7617605; doi:10.1192/bjp.2025.8)
Supplement: Shoesmith et al. supplementary material 7 — Shoesmith et al. supplementary material [file S000712502500008Xsup007.docx]

| Supplementary Table 7. Definitions/terminology used by authors to classify DAIs | | | | |  |
| --- | --- | --- | --- | --- | --- |
| Author (year) | **DAI type** | **Description of goals** | **Description of animal handler/animal teams** | **Description of dogs involved** | **Category** |
| Mental health conditions | | | | | |
| Allen et al. (2022) | Dog-assisted therapy | Involvement of dog to facilitate therapeutic goals associated with trauma | Professionally trained dog handlers observed all sessions but were not directly involved, and trained clinician prior to sessions | Team of trained service dogs via a local service dog organisation that performs breeding, training and placement of service dogs | Clear alignment |
| Calvo et al. (2016) | Dog-assisted therapy | No goals reported, and authors state these sessions were ‘activities’ between psychosocial rehabilitation sessions | Delivered by a member of the research team | Study only refers to ‘therapy dogs’ | Misalignment |
| Chen et al. (2021; 2022) | Dog-assisted therapy | Content designed to improve negative symptoms and general psychopathology symptoms associated with schizophrenia | Delivered by an animal-assisted therapist, an occupational therapist and a dog-handler pair (breeder). | Therapy dogs (and breeders) had been certified by the Professional Animal-Assisted Therapy Association of Taiwan | Clear alignment |
| Chu et al. (2009) | Dog-assisted activity | N/A | Delivered by a member of the research team | Trained dogs provided by trainers, but trainers did not accompany dogs during activities | Clear alignment |
| Shih et al. (2023) | Dog-assisted therapy | Content designed to improve skills in social interaction and emotional expression, with activities with dog designed to meet these goals | Delivered by qualified animal-assisted therapists with experience providing services to people with disabilities | Two service dogs with their handlers | Clear alignment |
| Stefanini et al. (2015) | Dog-assisted therapy | Structured sessions in accordance with individual therapeutic goals for each participant | Delivered by qualified animal-assisted therapists | Dogs trained for animal-assisted therapy and activities to the standards of Pet-Partner®, ex-Delta Society | Clear alignment |
| Stefanini et al. (2016) | Dog-assisted therapy | Intervention followed individual objectives for each participant and was integrated into the participant’s plan of care | Delivered by qualified animal-assisted therapists | Dogs trained for animal-assisted therapy and activities to the standards of Pet-Partner®, ex-Delta Society | Clear alignment |
| Wolynczyk-Gmaj et al. (2021) | Dog-assisted activity | N/A | Delivered by dog handler (with no further information) | Dog involved was owned by the dog handler | Clear alignment |
| Neurodevelopmental conditions | | | | | |
| Fung et al. (2014) | Dog-assisted therapy | Structured, therapist-directed play therapy. Involvement of dog to facilitate therapeutic outcomes | Delivered by qualified therapist with additional training in animal-assisted therapy | Qualified therapy dogs recruited from the Animal Asia Foundation. All dogs had passed an assessment of temperament, obedience skills, and coping with stress. | Clear alignment |
| Hill et al. (2020) | Dog-assisted therapy | Intervention began with goal setting, and all subsequent sessions consisted of occupation focused, goal-directed content | Delivered by qualified therapist with additional training in animal-assisted therapy | Trained therapy dog who had completed training and assessment as a therapy dog with her handler (qualified therapist delivering sessions) | Clear alignment |
| Meints et al. (2022; Study 2) | Dog-assisted activity | N/A | Delivered by volunteers who were members of Pets as Therapy | Dogs of volunteers who had passed assessments by independent dog behaviour specialists on their suitability to work with children | Clear alignment |
| Schuck et al. (2015) | Dog-assisted therapy | Content based on cognitive-behavioural therapies to enhance specific skills for children with ADHD. Involvement of dog aimed to facilitate these therapeutic outcomes | Delivered by qualified animal handlers with their dogs | Certified therapy dogs | Clear alignment |
| Schuck et al (2018a; 2018b); Nieforth et al. (2024) | Dog-assisted therapy | Content based on cognitive-behavioural therapies to enhance specific skills for children with ADHD. Involvement of dog aimed to facilitate these therapeutic outcomes | Delivered by qualified animal handlers with their dogs | Certified therapy dogs | Clear alignment |
| Scorzato et al. (2017) | Dog-assisted therapy | Content designed for rehabilitation, with different activities adapted to meet individual participant’s needs and abilities | Delivered by a qualified dog handler | Specially trained dogs meeting specific health prerequisites for animal-assisted interventions | Clear alignment |
| Vidal et al. (2020) | Dog-assisted therapy | Dogs facilitated the achievement of therapeutic goals set by the psychologist who conducted the intervention. | Delivered by a dog-assisted therapist and a psychologist | Certified therapy dogs who were trained and tested to work with people, and their mental and physical health care was strictly monitored | Clear alignment |
| Vidal et al. (2023) | Dog-assisted therapy | Content was designed to meet specific goals each week (e.g., frustration tolerance, emotional self-regulation, impulsivity management) | Delivered by a qualified dog-assisted therapy technician and a psychologist | Certified therapy dogs | Clear alignment |
| Villalta-Gil et al. (2009) | Dog-assisted therapy | Content had been developed to work on cognitive and social functioning, and was a structured programme to meet these goals | Delivered by a trained psychologist and qualified animal handler | Certified therapy dog | Clear alignment |
| Wijker et al. (2020; 2021) | Dog-assisted therapy | Semi-structured therapy protocol was developed by therapists and dog behavioural specialists, with the goals to reduce stress, depression, anxiety, and improve social skills | Delivered by a qualified psychologist who had completed advanced courses on dog behaviour | Trained service dogs, selected and trained by the Dutch Service Dog Foundation | Clear alignment |
| Dementia | | | | | |
| Baek et al. (2020) | Dog-assisted therapy | Content designed to enhance cognitive function, emotional status, problematic behaviours, and activities of daily living | Team consisted of three qualified animal mediation psychologists and one animal handler | Therapy dog who had completed all health, aptitude and technical tests and had experience of working in therapy sessions | Clear alignment |
| Bono et al. (2015) | Dog-assisted therapy | Not reported | Study only refers to ‘animals and their tutors’ | Study only refers to ‘animals and their tutors’ | Unclear alignment |
| Briones et al. (2021) | Dog-assisted therapy | Content designed to improve cognitive capacities, motor skills, and enhance social relations | Delivered by a trained animal-assisted therapy technician | Trained dogs with prior experience of working in therapy sessions | Clear alignment |
| Friedmann et al. (2015) | Dog-assisted activity | N/A | Delivered by a nurse practitioner experienced with working with older adults with dementia | Therapy dogs with experience of visiting care homes | Clear alignment |
| Majic et al. (2013) | Dog-assisted therapy | Content designed to enhance social interaction and functioning | Delivered by a qualified animal handler | Specially educated therapy dogs, with an exclusive relationship to their handler and a good level of obedience | Clear alignment |
| Menna et al. (2019) | Dog-assisted therapy | Goals not reported but based on formal reality orientation therapy with a specific protocol | Delivered by a veterinary zootherapist-handler | Study only refers to ‘therapy dog’ | Unclear alignment |
| Olsen et al. (2016a) | Dog-assisted activity | N/A | Delivered by a qualified dog handler | Dogs passed screening tests to assess personality traits and behaviour when interacting with humans | Clear alignment |
| Olsen et al. (2016b) | Dog-assisted activity | N/A | Delivered by a qualified dog handler | Dogs passed screening tests to assess personality traits and behaviour when interacting with humans | Clear alignment |
| Parra et al. (2021) | Dog-assisted therapy | Content of sessions was developed according to goals, aimed to enhance affective, behavioural, functional and cognitive domains | Delivered by a qualified dog-assisted therapy technician | Dogs were selected according to good health, high sociability, high impulse control and high learning ability. They were trained by their handlers for specific exercises of the intervention | Clear alignment |
| Parra et al. (2022) | Dog-assisted therapy | Goals set by occupational therapist and the dog-assisted therapy technician based on participants’ needs, and content was aligned to meet these goals | Delivered by a qualified dog-assisted therapy technician | Trained dog who had many years of dog-assisted therapy experience ad had passed temperament, health, and fitness for grooming tests | Clear alignment |
| Travers et al. (2013) | Dog-assisted therapy | Semi-structured session protocols were developed, and dogs were brought into therapy sessions to stimulate discussion | Delivered by a qualified therapist who was a veterinarian and had received training in dog-assisted therapy for people with dementia | Dogs owned by the therapist were involved, and were either Delta Society accredited or accredited by the consultant therapist | Clear alignment |
