# Supplementary material for: Dog-assisted interventions for children and adults with mental health or neurodevelopmental conditions: a systematic review
Source: Br J Psychiatry. Author manuscript; Available in PMC 2026 Feb 1. (PMC7617605; doi:10.1192/bjp.2025.8)
Supplement: Supplementary file 1 [file EMS202681-supplement-Supplementary_Materials.pdf]

Supplementary Material 1: Search Strategy for EMBASE

1. Emot$ OR ment$ OR psychological$ OR psychiatr$ OR risk OR disorder
2. ((Chronic$ or severe$ or persistent$) adj (mental$ OR psychological$) adj (disorder$ or ill$))
3. Exp Neuro$ Disorders/
4. Exp Neurodevelopmental Disorders/
5. Exp Intellectual Disability/
6. Exp autism spectrum disorder/ OR exp autistic disorder/
7. Autistic or autism or Asperger$
8. Exp Attention deficit disorder/ OR disruptive behaviour/ OR Attention deficit disorder with hyperactivity/
9. 1 OR 2 OR 3 OR 4 OR 5 OR 6 OR 7 OR 8
10. ((pet$ OR animal$ OR dog$ or canine$) adj2 (therap$ or activit$ or intervention$))
11. Exp animal assisted intervention/
12. Therap$ pet
13. Therap$ animal
14. Therap$ dog
15. Human animal bond$
16. Human animal interaction$
17. ((Assistance or service) adj2 (animal$ OR dog$))
18. 10 OR 11 OR 12 OR 13 OR 14 OR 15 OR 16 OR 17
19. 9 and 18
20. Limit 19 to English Language

Supplementary Material 2. Complete list of data extracted from included papers

1	Author/Year
2	Country
3	Research design
4	Registration number/name of trial registry
5	Setting
6	Participant demographics (e.g., age, gender, ethnicity)
7	Sample size (intervention/control)
8	Participant diagnosis
9	Diagnostic criteria
10	Dog-related inclusion/exclusion criteria
11	Recruitment rate
12	Attrition rate
13	Intervention attendance
14	Randomisation procedure
15	Blinding
16	Intervention name (e.g., therapy, activity)
17	Intervention content (including any tailoring/adaptation)
18	Intervention frequency/duration
19	Intervention space
20	Mode of delivery (e.g., group/individual)
21	Number and breed of dogs involved
22	Facilitator role
23	Facilitator training
24	Reference to infection control
25	Dog selection process
26	Dog training process
27	Information related to dog safety
28	Adverse events
29	Control name
30	Control content
31	Control frequency/duration
32	Control space
33	Control mode of delivery (e.g., group/individual)
34	Control facilitator role
35	Control facilitator training
36	Follow-up length
37	Outcome measures
38	Key findings
39	Key outcome means and standard deviations
40	Reported barriers
41	Reported enablers
42	Perceptions of intervention
43	Source of funding/other support

Supplementary Material 3. List of included papers (1-33)

1. Allen B, Shenk CE, Dreschel NE, Wang M, Bucher AM, Desir MP, et al. Integrating Animal-Assisted Therapy Into TF-CBT for Abused Youth With PTSD: A Randomized Controlled Feasibility Trial. Child maltreatment. 2022;27(3):466-77.
2. Baek SM, Lee Y, Sohng KY. The psychological and behavioural effects of an animal-assisted therapy programme in Korean older adults with dementia. Psychogeriatrics. 2020;20(5):645-53.
3. Bono AV, Benvenuti C, Buzzi M, Ciatti R, Chiarelli V, Chiambretto P, et al. Effects of animal assisted therapy (AAT) carried out with dogs on the evolution of mild cognitive impairment. Giornale di Gerontologia. 2015;63(1):32-6.
4. Briones MA, Pardo-Garcia I, Escribano-Sotos F. Effectiveness of a dog-assisted therapy program to enhance quality of life in institutionalized dementia patients. Clinical Nursing Research. 2021;30(1):89-97.
5. Calvo P, Fortuny JR, Guzmán S, Macías C, Bowen J, García ML, et al. Animal assisted therapy (AAT) program as a useful adjunct to conventional psychosocial rehabilitation for patients with schizophrenia: results of a small-scale randomized controlled trial. Frontiers in psychology. 2016;7:631.
6. Chen CR, Hung CF, Lee YW, Tseng WT, Chen ML, Chen TT. Functional Outcomes in a Randomized Controlled Trial of Animal-Assisted Therapy on Middle-Aged and Older Adults with Schizophrenia. International Journal of Environmental Research and Public Health. 2022;19(10):6270.
7. Chen TT, Hsieh TL, Chen ML, Tseng WT, Hung CF, Chen CR. Animal-Assisted Therapy in Middle-Aged and Older Patients With Schizophrenia: A Randomized Controlled Trial. Frontiers in Psychiatry. 2021;12:713623.
8. Chu C, Liu C, Sun C, Lin J. The effect of animal-assisted activity on inpatients with schizophrenia. Journal of Psychosocial Nursing & Mental Health Services. 2009;47(12):42-8.
9. Friedmann E, Galik E, Thomas SA, Hall PS, Chung SY, McCune S. Evaluation of a pet-assisted living intervention for improving functional status in assisted living residents with mild to moderate cognitive impairment: A pilot study. American Journal of Alzheimer's Disease and other Dementias. 2015;30(3):276-89.
10. Fung S-c, Leung AS-m. Pilot study investigating the role of therapy dogs in facilitating social interaction among children with autism. Journal of Contemporary Psychotherapy: On the Cutting Edge of Modern Developments in Psychotherapy. 2014;44(4):253-62.
11. Hill J, Ziviani J, Driscoll C, Teoh AL, Chua JM, Cawdell-Smith J. Canine Assisted Occupational Therapy for Children on the Autism Spectrum: A Pilot Randomised Control Trial. Journal of Autism and Developmental Disorders. 2020;50(11):4106-20.
12. Majic T, Gutzmann H, Heinz A, Lang UE, Rapp MA. Animal-assisted therapy and agitation and depression in nursing home residents with dementia: A matched caseecontrol trial. American Journal of Geriatric Psychiatry. 2013;21(11):1052-9.
13. Meints K, Brelsford VL, Dimolareva M, Marechal L, Pennington K, Rowan E, et al. Can dogs reduce stress levels in school children? effects of dog-assisted interventions on salivary cortisol in children with and without special educational needs using randomized controlled trials. PLoS ONE. 2022;17(6 June):e0269333.
14. Menna LF, Santaniello A, Gerardi F, Sansone M, Di Maggio A, Di Palma A, et al. Efficacy of animal-assisted therapy adapted to reality orientation therapy: measurement of salivary cortisol. Psychogeriatrics. 2019;19(5):510-2.
15. Nieforth LO, Guerin NA, Stehli A, Schuck S, Yi K, O'Haire ME. Observation of human-animal interaction for research (OHAIRE) behavior coding in a randomized control trial of children with attention-deficit hyperactivity disorder (ADHD) and a canine-assisted intervention. Frontiers in Psychiatry. 2024;15.
16. Olsen C, Pedersen I, Bergland A, Enders-Slegers MJ, Ihlebak C. Effect of animal-assisted activity on balance and quality of life in home-dwelling persons with dementia. Geriatric nursing (New York, NY). 2016;37(4):284-91.

17. Olsen C, Pedersen I, Bergland A, Enders-Slegers MJ, Patil G, Ihlebaek C. Effect of animal-assisted interventions on depression, agitation and quality of life in nursing home residents suffering from cognitive impairment or dementia: a cluster randomized controlled trial. International Journal of Geriatric Psychiatry. 2016;31(12):1312-21.
18. Parra EV, Garre JMH, Perez PE. Benefits of dog-assisted therapy in patients with dementia residing in aged care centers in Spain. International Journal of Environmental Research and Public Health. 2021;18(4):1-12.
19. Parra E, Manuel Hernández Garre J, Echevarría Pérez P. Impact of Dog-Assisted Therapy for Institutionalized Patients With Dementia: A Controlled Clinical Trial. Alternative Therapies in Health & Medicine. 2022;28(1):26-31.
20. Schuck SEB, Emmerson NA, Fine AH, Lakes KD. Canine-assisted therapy for children with ADHD: Preliminary findings from the Positive Assertive Cooperative Kids study. Journal of Attention Disorders. 2015;19(2):125-37.
21. Schuck SE, Emmerson NA, Abdullah MM, Fine AH, Stehli A, Lakes KD. A Randomized Controlled Trial of Traditional Psychosocial and Canine-Assisted Interventions for Children with ADHD: CABI International; 2018. 16 p.
22. Schuck SEB, Johnson HL, Abdullah MM, Stehli A, Fine AH, Lakes KD. The Role of Animal Assisted Intervention on Improving Self-Esteem in Children With Attention Deficit/Hyperactivity Disorder. FRONTIERS IN PEDIATRICS. 2018;6.
23. Scorzato I, Zaninotto L, Romano M, Menardi C, Cavedon L, Pegoraro A, et al. Effects of Dog-Assisted Therapy on Communication and Basic Social Skills of Adults With Intellectual Disabilities: A Pilot Study. Intellectual and developmental disabilities. 2017;55(3):125-39.
24. Shih CA, Yang MH. Effect of Animal-Assisted Therapy (AAT) on Social Interaction and Quality of Life in Patients with Schizophrenia during the COVID-19 Pandemic: An Experimental Study. Asian Nurs Res (Korean Soc Nurs Sci). 2023;17(1):37-43.
25. Stefanini MC, Martino A, Bacci B, Tani F. The effect of animal-assisted therapy on emotional and behavioral symptoms in children and adolescents hospitalized for acute mental disorders. European Journal of Integrative Medicine. 2016;8(2):81-8.
26. Stefanini MC, Martino A, Allori P, Galeotti F, Tani F. The use of Animal-Assisted Therapy in adolescents with acute mental disorders: A randomized controlled study. Complement Ther Clin Pract. 2015;21(1):42-6.
27. Travers C, Perkins J, Rand J, Bartlett H, Morton J. An evaluation of dog-assisted therapy for residents of aged care facilities with dementia. Anthrozoos. 2013;26(2):213-25.
28. Vidal R, Vidal L, Ristol F, Domenec E, Segu M, Vico C, et al. Dog-assisted therapy for children and adolescents with fetal alcohol spectrum disorders a randomized controlled pilot study. Frontiers in Psychology. 2020;11.
29. Vidal R, Vidal L, Lugo J, Ristol F, Domenec E, Casas T, et al. Dog-Assisted Therapy vs Relaxation for Children and Adolescents with Fetal Alcohol Spectrum Disorder: A Randomized Controlled Study. Journal of Autism and Developmental Disorders. 2023.
30. Villalta-Gil V, Roca M, Gonzalez N, Domenec E, Cuca, Escanilla A, et al. Dog-assisted therapy in the treatment of chronic schizophrenia inpatients. Anthrozoos. 2009;22(2):149-59.
31. Wijker C, Kupper N, Leontjevas R, Spek A, Enders-Slegers MJ. The effects of Animal Assisted Therapy on autonomic and endocrine activity in adults with autism spectrum disorder: A randomized controlled trial. General Hospital Psychiatry. 2021;72:36-44.
32. Wijker C, Leontjevas R, Spek A, Enders-Slegers MJ. Effects of Dog Assisted Therapy for Adults with Autism Spectrum Disorder: An Exploratory Randomized Controlled Trial. Journal of Autism and Developmental Disorders. 2020;50(6):2153-63.
33. Wolynczyk-Gmaj D, Ziolkowska A, Rogala P, Scigala D, Bryla L, Gmaj B, et al. Can dog-assisted intervention decrease anxiety level and autonomic agitation in patients with anxiety disorders? Journal of Clinical Medicine. 2021;10(21):5171.

Supplementary Material 4. Study characteristics

Author/ Year & Study Aim	Country/ Setting	Participants (n)	Diagnosis/ diagnostic criteria	Intervention	Control	Follow-up	Outcome measures	Main findings reported by study authors
				**Mental health conditions**				
**Allen et al. (2021) (71)**<br><br>To explore whether the integration of dogs into CBT is feasible and beneficial for children with PTSD compared to standard treatment.	USA, Outpatient treatment clinic	Children (ages 6-17); n = 33 (17 intervention; 16 control)	PTSD; PTSD Reaction Index for DSM-V	Trauma-focused CBT plus dog-assisted therapy	Trauma-focused CBT	None (post-intervention only)	Service satisfaction scale (SSS); UCLA PTSD reaction index; Strengths and difficulties questionnaire (SDQ); Moods and feelings questionnaire (MFQ); Screen for child anxiety related disorders (SCARED)	No significant improvements were found for self-reported PTSD symptom severity in the intervention group (pre-M = 39.5, SD = 18.44; post-M = 38.38, SD = 12.74). Significant improvements were reported in the control group (pre-M = 44.63, SD = 12.38; post-M = 28.09, SD = 24.09). The difference between groups was significant.<br><br>Significant improvements in caregiver-reported PTSD symptom severity in the intervention group (pre-M = 52.29, SD = 12.25; post-M = 35.85, SD = 14.81) and the control group (pre-M = 46.25, SD = 11.34; post-M = 22.64; SD = 16.43).
**Calvo et al. (2016) (50)**<br><br>To assess the effect of DAI as an adjunct to conventional psychosocial rehabilitation for people with schizophrenia.	Spain, Psychiatric hospital	Adults; n = 24 (16 intervention; 8 control)	Schizophrenia; DSM-IV-TR	Dog-assisted therapy	Activity from functional programme based on therapist's criteria (e.g., art therapy, group sports)	None (post-intervention only)	Positive and Negative Syndrome Scale (PANSS); EuroQol (EQ-5D)	Both groups showed significant improvements in positive symptomatology indicated by differences in pre- and post-treatment scores (intervention M = 5.28, SD = 4.78; control M = 7.87, SD = 4.29), and overall symptomology (intervention M = 10.00, SD = 8.70; control M = 12.63, SD = 13.57). Only the intervention group showed a significant improvement in

								negative symptomatology (intervention M = 5.64, SD = 8.19 vs. control M = 1.87, SD = 3.44).
**Chen et al. (2021) (47)**<br><br>To assess the efficacy of a DAI for participants with schizophrenia in a clinical setting.	Taiwan, Psychiatric rehabilitation ward and day care ward	Adults over 40 years; n = 40 (20 intervention; 20 control)	Schizophrenia; DSM-V	Dog-assisted therapy	Non-animal related nursing intervention and occupational therapy of usual treatment programmes	None (post-intervention only)	PANSS; Chinese Happiness Inventory (CHI); Depression, Anxiety Stress Scales-21 (DASS-21)	Significant improvements in the PANSS from pre-intervention (median = 11.5) to post-intervention (median = 11.0) in the intervention group compared to no change in the control group (pre-intervention median = 11.5; post-intervention median = 11.5).
**Chen et al. (2022) (14)**<br><br>To assess the efficacy of a DAI for participants with schizophrenia in a clinical setting.	Taiwan, Psychiatric rehabilitation ward and day care ward	Adults over 40 years; n = 40 (20 intervention; 20 control)	Schizophrenia; DSM-V	Dog-assisted therapy	Non-animal related nursing intervention and occupational therapy of usual treatment programmes	None (post-intervention only)	Montreal Cognitive Assessment (MoCA); Chair Stand test (CST); Timed Up and Go (TUG); 5-meter walk test (5MWT); Assessment of Communication and Interaction Skills (ACIS)	Significant improvements in social skills from pre-intervention (median = 65.0, IQR = 10.50) to post-intervention (median = 71.50, IQR = 6.0) in the intervention group. Post-intervention social skills in the intervention group significantly improved compared to the control group (median = 65.0, IQR = 12.50).
**Chu et al. (2009) (68)**<br><br>To explore the effects of a DAI on self-esteem, activities of daily living, and other psycho-physiological aspects among inpatients with schizophrenia.	Taiwan, Psychiatric ward	Adults up to age of 60; n = 30 (15 intervention; 15 control)	Schizophrenia; diagnostic criteria not specified	Dog-assisted activities	Treatment as usual	1 week	Rosenberg Self-Esteem Scale; extent of social support; increases and decreases in adverse psychiatric symptoms (specific measures not specified)	Significant improvements in self-esteem from baseline (M = 9.68) to follow-up (M = 15.71) in the intervention group compared to the control group (baseline, M = 9.48; follow-up, M = 9.29). Significant improvements in positive symptoms from baseline (M = 14.88) to follow-up (8.46) in the intervention group compared to the control group (baseline, M = 15.85; follow-up M = 16.54).

**Shih et al. (2023) (67)**<br><br>To evaluate the effectiveness of a DAI in improving social interactions and quality of life in participants with schizophrenia during the Covid-19 pandemic.	Taiwan, Psychiatric rehabilitation	Adults; n = 90 (45 intervention; 45 control)	Schizophrenia; DSM-V	Dog-assisted therapy	Discussion groups with short films about animals	3 months	Mental Health-Social Functioning scale; Social Adaptive Function Scale; Taiwanese version of the WHOQOL-BREF	Significant improvements in mental health and social functioning scores from baseline (M = 50.56) to post-intervention (M = 52.80) in the intervention group, but scores at 3-month follow-up declined (M = 46.07). Significant improves in quality of life from baseline (M = 79.33) to 3-month follow-up (M = 86.64) in the intervention group. There was a significant difference of the level of increase in quality of life scores from post-intervention to 3-month follow-up compared with the control group.
**Stefanini et al. (2015) (66)**<br><br>To compare the effects of a DAI with a standard treatment protocol in children and adolescents admitted to a psychiatric hospital.	Italy, Psychiatric hospital	Children and adolescents; n = 34 (17 intervention; 17 control)	Any acute psychiatric diagnosis; ICD-9	Dog-assisted therapy	Standard therapeutic protocol	3 months	Children Global Assessment Scale (C-GAS); observations	Significant changes in all observational scales from baseline to follow-up in the intervention group: participants showed higher participation (pre-M = 2.27, SD = 0.49; follow-up M = 3.21, SD = 0.60), more interaction with their animal (pre-M = 2.38, SD = 0.52; follow-up M = 3.19, SD = 0.43), and more socialised behaviour with adults (pre-M = 1.73, SD = 0.72; follow-up M = 3.17; SD = 0.72).
**Stefanini et al. (2016) (20)**<br><br>To examine the effects of a DAI on behavioural and emotional	Italy, Psychiatric hospital	Children and adolescents; n = 40 (20 intervention; 20 control)	Severe psychiatric diagnosis; ICD-9	Dog-assisted therapy	Standard therapeutic protocol	3 months	Global functioning; Youth Self-report; observational data	Significant improvements were found for global functioning in the intervention group (pre M = 39.75, SD = 6.88; post M = 48.65, SD = 6.87), but not in the control group (pre M = 42.15, SD = 6.04; post M = 48.65, SD = 6.87).

symptoms reported by children and adolescents admitted to a psychiatric hospital.								
**Villalta-Gil et al. (2009) (51)**<br><br>To assess the effectiveness of including a therapy dog in an intervention programme delivered to participants with schizophrenia.	Spain, Hospital	Adults; n = 21 (12 intervention; 9 control)	Schizophrenia; DSM-IV	Dog-assisted therapy	Same intervention without presence of dog	None (post-intervention only)	PANSS; Living Skills Profile (LSP); Brief WHO Quality of Life Assessment-Spanish Version (WHOQOL-BREF); Satisfaction with Treatment Questionnaire (STQ)	Significant improvements were found in the intervention group for social contact (pre-M = 13.67, SD = 2.67; post-M = 18.00, SD = 4.4); PANSS score (pre-M = 88.25, SD = 12.17; post-M = 73.64, SD = 18.69), and quality of life related with social relationships (pre-M = 2.08, SD = 0.79; post-M = 2.85, SD = 0.56), but no significant differences were found between the intervention and control group.
**Wolynczyk-Gmaj et al. (2021) (52)**<br><br>To investigate whether interactions with a dog can reduce anxiety symptoms in participants with anxiety or mixed-depressive anxiety disorders.	Poland, Hospital	Adults up to 60 years; n = 51 (25 intervention; 26 control)	Any anxiety or mixed depressive-anxiety disorder; ICD-10	Dog-assisted activity	Walk with medical student or doctor rather than dog and handler	None (post-intervention only)	Beck Depression Inventory (BDI): Ford Insomnia Response to Stress Test (FIRST; The Brief Symptom Inventory (BSI-18); The Visual Analogue Scale of level of fear; the State-Trait Anxiety Inventory (STAI); The Visual Analogue Scale of level of satisfaction with the intervention.	Significant improvements were found for state anxiety in the intervention group (pre-M = 46.30, SD = 9.63; post-M = 34.35, SD = 6.88) compared to the control group (pre-M = 47.24, SD = 11.01; post-M = 40.94, SD = 8.59).<br><br>Significant improvements were found in the intervention group for anxiety as a trait (pre-M = 53.90, SD = 7.78; post-M = 48.90, SD = 7.16) and fear (pre-M = 2.57, SD = 2.29; post-M = 1.05, SD = 0.97).

				**Neurodevelopmental conditions**				
**Fung et al. (2014) (53)**<br><br>To explore the effectiveness of therapy dogs to facilitate the social interaction of children with autism in a play context.	Hong Kong, School	Children (ages 6-10); n = 10 (5 intervention; 5 control)	Autism Spectrum Condition; DSM-IV	Dog-assisted play therapy	Play session with baby doll	None (post-intervention only)	Natural occurrence of child's social behaviour was measured based on observations	Significant increase in verbal social behaviour from pre- to post-intervention in the intervention group (*z* = -2.02, *p* = 0.43), compared to no significant difference in the control group (*z* = -1.48, *p* = .138).
**Hill et al. (2020) (19)**<br><br>To explore the impact of incorporating a therapy dog into occupational therapy sessions on the on-task behaviours and goal attainment of children with autism when compared to usual care occupational therapy.	Australia, Community (information and support service for individuals with ASC)	Children (ages 4-7); n = 22 (11 intervention; 11 control)	Autism Spectrum Condition; diagnostic criteria not specified	Dog-assisted occupational therapy	Usual care occupational therapy sessions	None (post-intervention only)	Observations to record 'on-task behaviour'; Canadian Occupational Performance Measure to assess goal attainment	Difference in percentage time of on-task behaviour between the intervention (median = 92.60%) and control group (median = 90.03%) showed an increase in on-task behaviour of +2.6% within the intervention group when compared to the control group, but this was not significant.
**Meints et al. (2022; study 2) (54)**<br><br>To explore the effects of a DAI on salivary cortisol levels in children in special educational needs schools.	UK, Special educational needs school	Children; n = 44	Autism Spectrum Condition; Attention Deficit Hyperactivity Disorder; Down Syndrome; other learning difficulties	Dog-assisted activities	Relaxation intervention or no treatment control group	None (post-intervention only)	Cortisol collection	No significant changes in cortisol levels for the intervention group (pre-M = .1381 μg/dL, SD = .06; post-M = .1158 μg/dL, SD = .04); or no treatment control group (pre-M = .1486 μg/dL, SD = .05; post-M = = .1486 μg/dL, SD = .06).

**Nieforth et al. (2024) (49)**<br><br>To report on the behaviour coding of video data collected as part of the Schuck et al. 2018 RCT and explore whether the presence of a dog would lead to an increase in social behaviours.	USA, Community	Children (ages 7-9); n = 35 (18 intervention, 17 control)	ADHD; K-SADS-PL (interview with coding criteria to guidelines of DSM-IV-TR)	Dog-assisted therapy based on cognitive-behavioural strategies and social skills training	Same curriculum but used toy dogs	6 weeks	OHAIRE coding system: summary scores were created for the following domains: Animal Social Interaction and Human Social Interaction (further separated into Human-Adult Social Interaction and Human-Peer Social Interaction).	No significant differences in how much children interacted with the live therapy dogs vs. control toy dogs. Children showed greater increases over time in human-directed social interactions in the presence of live dogs vs. toy dogs (p = 0.20).
**Schuck et al. (2015) (13)**<br><br>To explore whether a DAI leads to improvements in prosocial skills and problematic behaviours in children with ADHD.	USA, Community	Children (ages 7-9); n = 24 (12 intervention; 12 control)	ADHD; K-SADS-PL (interview with coding criteria to guidelines of DSM-IV-TR)	Dog-assisted therapy based on cognitive-behavioural strategies and social skills training	Same curriculum but used toy dogs	6 weeks	ADHD-Rating-Scale-Fourth Edition, Home, and School Version (ADHD-RS-IV); Social Skills Improvement System-Rating Scales, Parent Form (SSIS-RS); Social Competence Inventory (SCI)	Significant improvements found for social skills in both intervention (pre-M = 75.00, SE = 8.31; post-M = 92.00, SE = 13.88) and control groups (pre-M = 76.00, SE = 10.90; post-M = 86.42, SE = 1.94).<br><br>Significant improvements found for problem behaviours in both intervention (pre-M = 129.17, SE = 11.59; post-M = 112.67, SE = 14.72) and control groups (pre-M = 127.67, SE = 9.21; post-M = 115.33, SE = 9.67).<br><br>The severity of ADHD symptoms declined in both groups but participants in the intervention group exhibited greater reductions in the severity of ADHD symptoms (M

								at week 2 = 28.17, SE = 6.89; M at week 10 = 19.00, SE = 7.34).
**Schuck et al. (2018a) (48)**<br><br>To explore whether a DAI is a feasible approach to improve self-esteem among children with ADHD.	USA, Community	Children (ages 7-9); n = 81 (41 intervention; 40 control)	ADHD; K-SADS-PL (interview with coding criteria to guidelines of DSM-IV-TR)	Dog-assisted therapy based on cognitive-behavioural strategies and social skills training	Same curriculum but used toy dogs	6 weeks	The Self-Perception Profile for Children (SPSS); Social Skills Improvement System-Parent Form (SSIS-P); ADHD-RS-IV	Self-reported scores of behavioural conduct and social competence were significantly higher post-intervention than pre-intervention in the intervention group (z = 2.320, p = .021, z = 2.631, p = .008, and z = 2.541, p = .011, respectively). Pre/post differences were not found for those in the control group.
**Schuck et al. (2018b) (15)**<br><br>To evaluate the efficacy of the intervention and control group for improving ADHD symptoms and social outcomes.	USA, Community	Children (ages 7-9); n = 81 (41 intervention; 40 control)	ADHD; K-SADS-PL (interview with coding criteria to guidelines of DSM-IV-TR)	Dog-assisted therapy based on cognitive-behavioural strategies and social skills training	Same curriculum but used toy dogs	6 weeks	ADHD-RS-IV; SSIS-P; The Social Competence Inventory (SCI)	Significant improvements were found for ADHD symptom severity in the intervention group from week 2 (M = 32.32, SD = 8.62) compared to immediately post-intervention (M = 20.86, SD = 7.66).<br><br>Significant improvements within the intervention group were found for social skills (baseline M = 79.54, SD = 11.09; follow-up M = 91.00, SD = 9.39); problem behaviours (baseline M = 125.98, SD = 14.39; follow-up M = 109.37, SD = 12.43), and prosocial orientation (baseline M = 3.02, SD = 0.58; follow-up M = 3.42, SD = 0.51).
**Scorzato et al. (2017) (55)**<br><br>To determine the effects of a DAI on the behaviour,	Italy, Community centres	Adults; n = 39 (21 intervention; 18 control)	Intellectual disability; ICD-10	Dog-assisted therapy	Comparable activities but with no dog	None (post-intervention only)	International Classification of Functioning, Disability and Health (ICF); Behavioural Assessment	Significant improvements within the intervention group were found for attention to movement (baseline M = 6.5; follow-up M = 15.5), visuomotor coordination (baseline M = 10.48; follow-up M = 14.67), exploratory play

communication and social skills of adults with intellectual disabilities.							Battery (BAB); Learning Accomplishment Profile (LAP)	(baseline M = 15.55; follow-up M = 24.12), and motor imitation (baseline M = 18.71; follow-up M = 25.71).<br><br>Significant improvements were found for social skills in the intervention group (baseline M = 57.4; follow-up M = 67.1) but not in the control group (baseline M = 53.5; follow-up M = 50.0).
**Vidal et al. (2020) (72)**<br><br>To evaluate the efficacy of a DAI in children and adolescents with FASD in relation to its effects on social skills, internalised and externalised symptomatology and on severity of FASD symptoms.	Spain, Hospital	Children and adolescents; n = 33 (17 intervention; 16 control)	Fetal alcohol spectrum disorder (FASC); established by senior psychiatrists	Dog-assisted therapy	Treatment as usual	1 week	Child Behaviour Checklist (CBCL of Achenbach) parent version; Social Skills Improvement System-Parent Form (SSIS-P); Clinical Global Impression Scale for Severity (CGI-S)	Significant improvements were found for social skills in the intervention group (baseline M = 73.25, SD = 17.08; follow-up M = 81.56, SD = 16.58) compared to the control group (baseline M = 69.31, SD = 13.86; follow-up M = 69.46, SD = 13.51).<br><br>Significant improvements were found for FASC severity in the intervention group (baseline M = 3.76, SD = 0.43; follow-up M = 3.18, SD = 0.39) compared to the control group (baseline M = 3.44, SD = 0.5; follow-up M = 3.44, SD = 0.61).
**Vidal et al. (2023) (56)**<br><br>To evaluate the efficacy of a DAI in children and adolescents with FASD in relation to its effects on social skills, internalised	Spain, Hospital	Children and adolescents; n = 71 (38 intervention; 33 control)	Fetal alcohol spectrum disorder (FASC); established by senior psychiatrists	Dog-assisted therapy	Relaxation intervention	None (post-intervention only)	Social Skills Improvement System-Parent Form (SSIS-P); Child Behaviour Checklist (CBCL of Achenbach) parent version; KidScreen 27 Parent version; Clinical Global	Significant improvements were found for social skills in the intervention group (baseline M = 68.42, SD = 18.46; post-intervention M = 73.71, SD = 17.51) compared to the control group (baseline M = 71.56, SD = 19.34; post-intervention M = 73.75, SD = 20.46).

and externalised symptomatology, severity of FASD symptoms, and quality of life.							Impression Scale for Severity (CGI-S)	Significant improvements were found for FASC severity in the intervention group (baseline M = 4.16, SD = 0.91; post-intervention M = 3.55, SD = 0.76), but these were not significantly different from the control group.
**Wijker et al. (2020) (21)**<br><br>To explore the effects of a DAI in adults with ASD, focusing on self-perceived stress, social responsiveness, and psychological symptoms (e.g., depression and anxiety).	Netherlands, Mental health care organisation	Adults up to 60 years; n = 53 (27 intervention; 26 control)	Autism Spectrum Condition; ADI-R and DSM-V	Dog-assisted therapy	Wait list control group	10 weeks	Perceived Stress Scale (PSS); Symptom Checklist-90 revised (SCL-90-R); Social Responsiveness Scale for Adults (SRS-A); Rosenberg Self-Esteem Scale	Significant improvements were found for social skills in the intervention group, but not in the control group. No significant improvements were found for stress, symptoms, or self-esteem.
**Wijker et al. (2021) (23)**<br><br>To examine the psychophysiological responses of adults with ASC to a DAI session.	Netherlands, Mental health care organisation	Adults up to 60 years; n = 53 (27 intervention; 26 control)	Autism Spectrum Condition; ADI-R and DSM-V	Dog-assisted therapy	Wait list control group	10 weeks	Cortisol and alpha-amylase collection	Significant decreases were found for cortisol at the end of an intervention session (M = 8.74, SD = 3.14) compared to the start of the session (M = 10.04, SD = 3.14). No significant differences were found for cortisol or alpha-amylase levels pre- and post-intervention or compared to the control group.
**Dementia**								
**Baek et al. (2020) (57)**	South Korea, Hospital	Older adults (65 years+); n = 36 (18 intervention; 18 control)	Dementia; Korean version of the MMSE	Dog-assisted therapy	'Non-equivalent control	None (post-intervention only)	Korean version of the MMSE; Faces Rating Scale; Korean version of	No significant group differences post-intervention in mood status (intervention M = 1.57, SE = 0.17 vs. control M = 2.57, SE =

To investigate the psychological and behavioural effects of a DAI on cognitive function, emotional state, problematic behaviours and activities of daily living among adults with dementia.					group' (not specified)		the Cornell Scale for Depression in Dementia (CSDD); Barthel Index; Problem behaviour assessment tool	0.17); depressive symptoms (intervention M = 6.35, SE = 4.06 vs. control M = 10.71, SE = 1.27); or problematic behaviours (intervention M = 7.07, SE = 0.96 vs. control M = 8.64, SE = 0.89).
**Bono et al. (2015) (58)**<br><br>To examine the clinical effect of a DAI to non-hospitalised participants with dementia.	Italy, Memory clinic	Older adults (65 years+); n = 32 (16 intervention; 16 control)	Minor to mild Alzheimer's Disease; diagnosed as outpatients	Dog-assisted therapy	No information provided	None (post-intervention only)	Barthel Index; Alzheimer Disease Assessment scale (ADAS); CSDD	Significant improvements in depressive symptoms at follow-up in the intervention group (M = 8.1, SE = 2.3) compared to the control group (M = 4.4, SE = 1.4).
**Briones et al. (2021)<br>(18)**<br><br>To assess the effectiveness of a DAI to enhance quality of life in participants with dementia in a public care home.	Spain, Residential aged care facility	Older adults (65 years+); n = 32 (16 intervention; 18 control)	Dementia; MEC-10	Dog-assisted therapy	Treatment as usual	None (post-intervention only)	Quality of Life in Alzheimer's Disease (QoL-AD); Barthel Index; MEC-30	Both groups showed an improvement in quality of life from pre-intervention (intervention M = 30.85, SD = 1.50; control M = 29.00, SD = 1.6) to post-intervention (intervention M = 32.46, SD = 1.27; control M = 31.50, SD = 1.40) but the improvement was only significant in the control group.
**Friedmann et al. (2015)<br>(59)**<br><br>To evaluate the use of structured activities with a dog to prevent	USA, Assisted living facilities	Adults over 55 years; n = 40 (22 intervention; 18 control)	Dementia; MMSE	Pet-assisted living (PAL) intervention (dog-assisted activities)	Reminiscing programme	None (post-intervention only)	Barthel Index; CSDD; Zimmerman's short version of Apathy Evaluation Scale (AES); Cohen-Mansfield Agitation Inventory (CMAI)	Significant improvements in depressive symptoms from pre-intervention (M = 8.91, SE = 1.21) to post-intervention (M = 5.21, SE = 0.77) in the intervention group compared to the control (baseline M = 8.28, SE = 1.30; follow-up M = 8.67,

deterioration of physical, emotional and behavioural function in participants with dementia.								SE = 1.51). No significant group differences were found for apathy or agitation.
**Majic et al. (2013) (60)**<br><br>To investigate the efficacy of a DAI on symptoms of agitation, aggression and depression in participants with dementia.	Germany, Nursing homes	Older adults; n = 65 (intervention 30; control 35)	Moderate to severe dementia; MMSE and DSM-IV	Dog-assisted therapy	Treatment as usual	None (post-intervention only)	CMAI; Dementia Mood Assessment Scale (DMAS)	No significant differences over time in the intervention group for mood (pre-M = 26.85, SD = 16.91; post-M = 21.59, SD = 16.36) or agitation (pre-M = 46.78, SD = 16.89; post-M = 45.96, SD = 15.87). No significant differences were found between the intervention and control group for mood or agitation.
**Menna et al. (2019) (61)**<br><br>To evaluate whether a DAI adapted to formal Reality Orientation Therapy could modify salivary cortisol levels in participants with dementia.	Italy, Adult day care centre	Older adults; n = 22 (11 intervention; 11 control)	Mild to moderate Alzheimer's Disease; diagnostic criteria not specified	Dog-assisted therapy using Reality Orientation Therapy protocol	Activities based on Reality Orientation Therapy protocol	None (post-intervention only)	MMSE; Geriatric Depression Scale (GDS); cortisol collection	Depression levels significantly decreased in the intervention group (pre-M = 14.3, SD = 2.6; post-M = 12.3, SD = 2.37), but significantly increased in the control group (pre-M = 13.6, SD = 4.3; post-M = 14.5, SD = 4.5).
**Olsen et al. (2016a) (64)**<br><br>To examine the effectiveness of a DAI on quality of life in participants with dementia.	Norway, Day centres	Older adults (65 years+); n = 80 (42 intervention; 38 control)	Mild to severe dementia; MMSE	Dog-assisted activities	Treatment as usual	3 months	Quality of Life in late-stage dementia (QUALID)	No significant improvements for quality of life were reported in the intervention group (baseline M = 15.89; follow-up M = 16.65) or the control group (baseline M = 15.94; follow-up M = 15.23).

**Olsen et al. (2016b) (65)**<br><br>To examine the effects of a DAI on depression, agitation, and quality of life in participants with dementia.	Norway, Nursing homes	Older adults (65 years+); n = 58 (28 intervention; 30 control)	Dementia; MMSE	Dog-assisted activities	Treatment as usual	3 months	Behavioural Activity Rating Scale (BARS); CSDD; QUALID; Clinical Dementia Rating Scale	Depression levels significantly improved in the intervention group (baseline M = 8.35, SD = 4.65; follow-up M = 7.41, SD = 5.01), compared to the control group (baseline M = 6.88, SD = 4.70; follow-up M = 9.58, SD = 6.61).<br><br>No significant differences in agitation were found between intervention (baseline M = 23.44, SD = 7.64; follow-up M 24.87, SD = 8.34) or control groups (baseline M = 23.19, SD = 11.39; follow-up M = 24.00, SD = 13.20).
**Parra et al. (2021) (62)**<br><br>To evaluate the benefits that a DAI could provide for affective, behavioural, cognitive, and functional aspects of participants with dementia.	Spain, Care centres	Older adults (65 years+); n = 371 (186 intervention; 185 control)	Dementia; MMSE	Dog-assisted therapy	Treatment as usual	None (post-intervention only)	MEC-30; Modified Barthel Index; CSDD; Neuropsychiatric Disorders Inventory (NPI)	Depression levels significantly improved in the intervention group (pre-M = 8.14, SD = 6.92; post-M = 4.33, SD = 5.4) and significantly worsened in the control group (pre-M = 8.14, SD = 6.91; post-M = 9.34, SD = 7.1).<br><br>Agitation levels significantly improved in the intervention group (pre-M = 10.67, SD = 19.2; post-M = 5.3, SD = 14.8), and significantly worsened in the control group (pre-M = 11.32, SD = 20.5; post-M = 12.9, SD = 22.2).
**Parra et al. (2022) (16)**<br><br>To evaluate the benefits that a DAI	Spain, Nursing home	Older adults (65 years+); n = 21 (7 people with dementia in intervention; 7	Dementia; MMSE	Dog-assisted therapy	Treatment as usual	None (post-intervention only)	MMSE; Modified Barthel Index; The Yesavage Geriatric Depression Scale (GDS-VE); NPI	Significant differences found between the intervention group and the control group in agitation and depression, with *p* < .037 and .011, respectively.

could provide for emotional, behavioural, cognitive, and functional aspects of participants with dementia.		people with dementia in control; 7 without dementia in 'healthy group')						
**Travers et al. (2013)** **(63)**<br><br>To assess the effectiveness of a DAI on mood, quality of life, and psychosocial functioning in participants with dementia.	Australia, Aged care facilities	Older adults; n = 67 (34 intervention; 33 control)	Mild to moderate dementia; resident's medical record	Dog-assisted therapy	Human-therapist-only	None (post-intervention only)	Modified MMSE (MSE-3MS) QoL-AD; Medical Outcomes Study 36-item Short Form Health Survey v1 (SF-36); GDS; Multidimensional Observational Scale for Elderly Subjects (MOSES)	Significant improvements were found for quality of life in one facility post-intervention (M = 38.1, SD = 4.4) compared to the control group (M = 33.2, SD = 5.3) but were significantly lower in two other facilities (M, 34.0, SD = 7.2; M = 34.7, SD = 4.9, respectively) compared to their respective control groups (M = 38,9, SD = 5.9; M = 39.6, SD = 6.1, respectively). No significant group differences were found for other measures.

(PTSD) Post-traumatic stress disorder; (ADHD) attention deficit hyperactivity disorder; (DSM) Diagnostic and Statistical Manual of Mental Disorders; (ICD) International Statistical Classification of Diseases and Related Health Problems; (ADI-R) Autism Diagnostic Interview – Revised; (CBT) Cognitive-behavioural therapy; (OHAIRE) Observation of Human-Animal Interaction for Research Coding System; (MMSE) Mini mental state examination; (M) mean; (SD) standard deviation; (SE) standard error

Supplementary Material 5. Intervention characteristics

Author/ Year	Content	Frequency/ duration	Group/ individual	Dog breed	Delivered by	Space
**Allen et al. (2021)**	The first four sessions focused on providing psychoeducation, teaching relaxation and coping skills. Session 5 - 9 focused on the narrative of the trauma experience while reinforcing approach behaviours that facilitate effective coping. The final four sessions included the youth discussing the trauma narrative with the caregiver in a joined session. A dog was required to be in the room for all sessions where the youth were engaged in treatment, including the joined session with the caregiver. The manner in which the youth interacted with the dog was not dictated. The clinician provided cues for the dog to approach or move away in accordance with the youth's preferences. During situations where the youth appeared stressed, the clinician offered interaction with the dog as a coping skill alongside previously taught skills but did not otherwise introduce the dog into the activities of the sessions	12 x 90 minute sessions	Individual sessions (with some joined session with caregiver)	5 x Labrador retrievers	Clinicians trained to Trauma-focused CBT certification requirements and trained by handlers in understanding canine body language	Therapy room
**Baek et al. (2020)**	Sessions grouped into three stages (introductory, developmental, and final stages). Aims of the developmental stage were to enhance interaction, emotional stability, ADL, and cognitive function. Participants trimmed dogs' fur, trained, and walked them, and talked to them about their feelings	2 x 60 minute sessions/ weekly for 8 weeks	Group	Breed not specified	Animal mediation psychologist	Large space in hospital for both participant and therapy dog (99m^2)
**Bono et al. (2015)**	Activities included cognitive stimulation (spatial-temporal orientation, mnemonic stimulation, dog 'introduction' to the participant, identification of the dog breed, learning basic commands, etc.); communication (learning the body language of the animal); motor activity strolling with the dog, petting the dog, throwing a small ball), and wellbeing and entertainment	Biweekly, 60 minute sessions for 8 months	Individual	1 x Golden Retriever, 2 x Border Collies, 1 x Weimaraner	Animal trainer	Rooms at the Memory Clinic

**Briones et al. (2021)**	Content of the sessions were planned to achieve specific objectives. The technicians established the content of the sessions, which, responding to specific objectives, comprised activities focused on cognitive and motor functions, communication, recreational pursuits, and well-being	Weekly 50 minute sessions for 9 months	Group	1 x Labrador retriever, 1 x golden retriever	Animal-assisted therapy technicians	Room used exclusively for intervention
**Calvo et al. (2016)**	Three types of sessions were involved: (1) sessions to develop the emotional bond between participants and dogs. The participants were taught to handle and take care of dogs correctly. In this type of session, concepts of animal welfare and responsible ownership were explained and practiced; (2) sessions involving walking the dogs. During the first half of the programme, the dogs were walked in a large natural park so patients could learn to walk the dogs in a calm and controlled manner. For the rest of the programme, participants walked the dogs in the city so they could experience dog walking in a social context; (3) sessions to train and play the dogs: patients learned to give instructions to the dogs and train them using positive reinforcement training techniques	2 x weekly 60 minute sessions for 6 months	Group	Not specified	Animal-assisted therapy technicians	Various settings as sessions involved leaving the hospital to various locations (e.g., parks/city for dog walking).
**Chen et al. (2021; 2022)**	The primary goal was to improve negative symptoms and general psychopathology symptoms. Secondary goals were aimed at improving positive symptoms and patient wellbeing. The dog approached the participants in turn, and each participant walked the dog around the classroom. Each session was carried out with a similar structure: 15-min warm up, 45-min therapeutic activities, 5 min feedback. In the warm-up, the therapist greeted each participant, introduced the dog, reviewed the contents of the last session and oriented participants to the activities. There were four types of therapeutic activities: activity for positive emotion (e.g., touching the dog, massaging the dog, playing with the dog), social activity (e.g., cooperating in the games with each other and the dog), cognitive activity (e.g., training the dog), and physical activity (e.g., walking, handling,	60 minute sessions over a 12 week period	Group	1 x Corgi, 1 x Labrador	Animal-assisted therapist, occupational therapist, and dog-handler pair	Conducted in a spacious and quiet classroom with the participants sat in a semi-circle

	feeding, grooming the dog). Each activity was performed for three sessions with gradually increasing levels of difficulty. Therapists gave feedback on what the group did during the therapeutic activities, asked how they felt with the dog, and previewed the content of the next session					
**Chu et al. (2009)**	A different activity was held each week. Session one: Introduction and getting acquainted with the dog; Session two: walk with the dogs; Session three: walk with the dogs over barriers; Session four: carry the dogs on a cart, touch the dogs, play with the dogs; Session five: throw and chase big and small balls and play with dogs; Session six: throw and fetch small balls and play with the dog; Session seven: raise the dogs attention/play with the dogs; Session eight: group discussion and exchange of feelings. Participants were encouraged to interact with the dogs and freely express their feelings and thoughts. The activities were not rigidly designed; rather, they allowed the patients and animals to interact freely. No specific goals or achievements were set before each visit	Weekly x 50 minute sessions over 2 months	Group	Not specified	Research investigator	Hospital garden but took place in the activity hall during inclement weather and during first 2 weeks to not distract participants
**Friedmann et al. (2015)**	Programme included activities designed to encourage maintenance of physical and emotional function: feeding the dog, brushing the dog's teeth and hair, dressing the dog in a bandana (activities of daily living), throwing a ball, grooming the dog (range of motion), adjusting a collar, feeding the dog a treat, petting the dog (small motor skills) opening treats and giving the dog a treat (sequencing events), and talking to the dog (social skills)	1 x 60-90 minute session/ biweekly for 12 weeks	Group	Cardigan Welsh Corgi	Nurse practitioner	Group living room
**Fung et al. (2014)**	The children could have physical contact with the dogs, including such activities as identifying their body parts and petting them. The children would also be guided to take care of them, for example, combing their hair and feeding them. Moreover, they would act as social partners of the children. The children would walk the dogs and throw balls to them	20 minute sessions, 3 x weekly for 7 weeks	Individual	2 x Golden retrievers	Trained animal-assisted therapist	All sessions conducted at the school in a multipurpose room with which the participants

						were already familiar
**Hill et al. (2020)**	All therapy sessions consisted of occupation focused, goal directed interventions. For example, if the goal was the development of a dynamic tripod grasp in preparation for starting school, the activity would involve colouring in cartoon bones to symbolise the number of treats the child would be able to give to the therapy dog	1 x weekly 60 minute sessions for 9 weeks	7 sessions with parent and child; 2 parent sessions for goal setting (child not present)	Labradoodle	Trained animal-assisted therapist	Sessions took place within an animal-assisted psychology practice, specifically suited to facilitation of therapy dogs working within the clinic
**Majic et al. (2013)**	In the initial session, the dog was presented to the participant, introducing the name of the dog and when the dog would be visiting. The sessions started with interaction between the therapy guide and the dog, the participant speaking to the dog, and then physical interaction such as stroking/petting the dog. The last 15 minutes of the sessions included spontaneous dynamic processes between dog and participants, allowing for free interaction between the two	1 x 45-minute session, weekly for 10 weeks	Not specified	Border collies	Qualified dog handler	Not specified
**Meints et al. (2022; study 2)**	Each intervention began and ended with the child greeting the dog as advised by the dog handler and time for petting the dog if appropriate—this phase of active contact lasted roughly 5 minutes. The next and central part of the session was based on the dog with children learning facts about the dogs from the handler, watching the dog, talking about, and interacting with the dog. This was child-led and sessions varied in verbal content depending on questions children asked about the dog, and it lasted approximately 10 minutes. The last part of the session was 'saying goodbye', and again a chance to pet the dog. Any child worried about meeting the dog did not need to take part–but, if they wanted to, was purposely seated next to the researcher and the greeting process was child-led.	8 x 20 minute sessions held twice a week for four weeks	Either individual or in small groups (up to 7 children)	1 x Greek Hare-Hound, 2 x Cavalier King Charles Spaniel and Miniature Poodle crossbreed, 1 x Labrador and miniature Poodle crossbreed,	Professional animal handler	Not specified

				2 x German Short-Haired Pointers, 2 x Miniature Schnauzers, 3 x Labradors and 1 x Labrador crossbreed, 2 x Tibetan Mastiffs, 1 x Border Terrier, 1 x Scottish Terrier, 1 x Lurcher, 1 x Clumber Spaniel, 1 x Yorkshire Terrier, 1 x Pekingese, 1 x Smooth Collie, 1 x Cocker Spaniel and 1 x Golden Retriever.		
**Menna et al. (2019)**	Therapy with dog using Reality Orientation Therapy (ROT) – first 15 minutes involving introducing the dog, and the next 20 minutes included structured activity (e.g., play fetch, hiding the ball, caring for the dog). The last 10 minutes involved the same ending activity each time. There was a closing speech and washing of hands. The interactions with the animal were structured according to ROT, with some adaptations.	1 x weekly session for 3 months	Group	Labrador retriever	Veterinarian	Not specified

**Olsen et al. (2016a)**	Various activities (e.g., petting the dog, brushing the dog, feeding the dog, throwing a toy for the dog to fetch). Idea of the sessions was that physical functions would be enhanced by doing different physical tasks (e.g., bending down, reaching out, lifting arms, throwing balls). It was also assumed cognition, self-efficacy and fine motor skills may be enhanced if participants were to give the dog commands and reward the dog. All sessions followed a main protocol but were individually tailored to each participant based on the care workers' knowledge of the individual.	1 x 30 minute session/ twice a week for 12 weeks	Groups of 3 – 7	Not specified	Qualified dog handler	Randomly seated in a half circle in a room
**Olsen et al. (2016b)**	Various activities (e.g., petting the dog, brushing the dog, feeding the dog, throwing a toy for the dog to fetch). Idea of the sessions was that physical functions would be enhanced by doing different physical tasks (e.g., bending down, reaching out, lifting arms, throwing balls). It was also assumed cognition, self-efficacy and fine motor skills may be enhanced if participants were to give the dog commands and reward the dog. All sessions followed a main protocol but were individually tailored to each participant based on the care workers' knowledge of the individual.	1 x 30 minute session/ twice a week for 12 weeks	Groups of 3 – 7	Not specified	Qualified dog handler	Randomly seated in a half circle in a room
**Parra et al. (2021)**	First sessions introduced participants and dog, and the following sessions consisted of working on the area under study through direct interaction with the dog and a final phase of farewell. The strategies used according to the objectives to be worked on were: (1) affective area - caressing and brushing the dog; (2) behavioural area – promotion of sustained attention to the dog and to the activity; (3) functional area – activities aimed at working motor skills, and (4) cognitive area – working on spatial and temporal orientation.	1 x 45 minute session weekly for 8 months	Groups of 10	Not specified	Dog-assisted therapy technician	Not specified
**Parra et al. (2022)**	The objectives for each session were set by the occupational therapist and the DAT technician based on participants' needs. For large group sessions, activities included: (1) Perringo, a bingo game in which the dog has the numbers in a container on its harness'	2 x 45 minute weekly sessions over a	Groups (large or small depending on session)	Not specified	Dog-assisted therapy technician and occupational therapist	Not specified

	chest panel; (2) snakes and ladders, a team game in which participants need to pass a series of tests with the dog until reaching the last box with the last test; (3) Simon Says, a game in which participants must imitate the positions adopted by the dog; and (4) You Say, a game in which participants must ask the dog to adopt a position depending on the colour shown by the dice. For small group sessions, content was designed to work on specific therapeutic objectives. Activities included: six sessions to establish a bond with the dog, during which the dog was presented as an animal and as an individual; participants received information about its needs, learning, methods of communication with the dog, and correct handling; (2) six sessions with different topics—animals, seasons of the year, numbers, words, and food—aimed primarily at stimulating the cognitive area; (3) six sessions aimed primarily at stimulating the functional area—fine and gross motor skills, walking, and balance and coordination; and (4) six sessions aimed primarily at socioemotional stimulation, recognising and expressing emotions, interaction with others, and participation.	period of 6 months				
**Scorzato et al. (2017)**	The activities were structured according to a pattern of growing complexity and interaction (i.e., from simple observation with no interaction toward more complex group activities). The programme was designed to be as inclusive as possible, and the different activities were conceived to be simple and flexible in order to adapt to each participant's needs and abilities.	30 minute 1 x weekly sessions for 20 sessions	Group of 3	2 x Bernese Mountain Dogs, 1 x Bergamasco Shepherd, 1 x French Bulldog	Professional dog handler	A room at the Pet Therapy Centre, about 42sqm and included tables, chairs and different toys
**Schuck et al. (2015)**	Children were supported in 'training' basic commands (come, sit, stay) with certified therapy dogs. The social skills curriculum used within the programme combines cognitive-behavioural and behavioural theories with behaviour modification techniques and social problem solving strategies to promote adaptive skill acquisition. The model, derived from research on programmes designed to teach social skills and friendship making	2 x weekly for 12 weeks (one weekday evening for 2 hours and one weekend	Group	Not specified	Professional dog handler	Not specified

	skills has been adapted for children with ADHD and utilises a combination of didactic instruction, modelling, and role-play, while implementing a token economy, group and individual contingencies, and differential positive reinforcement of adaptive behaviours that are incompatible with problem behaviours.	day for 2.5 hours)				
**Schuck et al. (2018a; 2018b); Nieforth et al. (2024)**	Children were supported in 'training' basic commands (come, sit, stay) with certified therapy dogs. The social skills curriculum used within the programme combines cognitive-behavioural and behavioural theories with behaviour modification techniques and social problem solving strategies to promote adaptive skill acquisition. The model, derived from research on programmes designed to teach social skills and friendship making skills has been adapted for children with ADHD and utilises a combination of didactic instruction, modelling, and role-play, while implementing a token economy, group and individual contingencies, and differential positive reinforcement of adaptive behaviours that are incompatible with problem behaviours.	2 x weekly for 12 weeks (one weekday evening for 2 hours and one weekend day for 2.5 hours)	Group	Not specified	Professional dog handler	Not specified
**Shih et al. (2023)**	The intervention was implemented through support groups to help participants develop their skills in social interaction and emotional expression. Week 1 and 2 involved building relationships with peers and the dogs. Weeks 3 – 6 involved brief interactions with the dogs (e.g., instructing the dog to sit down, walking with the dog, feeding the dog). Weeks 7-12 involved deeper interactions with the dog (e.g., grooming and talking to the dog, going through tunnels with the dogs, interacting with the dogs without seeing the dogs, and engaging in creative activities about the dog).	Weekly sessions (60 minutes) for 12 weeks	Group	Not specified	Researchers and social workers, along with the participation of two animal-assisted therapists.	Reception hall
**Stefanini et al. (2015)**	Consisted of structured sessions in accordance with the individual therapeutic goals for each patient. This programme had four phases: 1) familiarisation with the animal and the handler; 2) individual intervention; 3) group activity; 4) discussion of the CAT experience. During the session, participants interacted with a dog	Weekly sessions (approx. 45 minutes) for 3 months	Both individual and group	Not specified	Qualified animal handler	Conducted in the hospital's garden or in an activity room when weather was bad.

	and the handler. The repertoire of CAT intervention covers a wide range of activities and consists in play activities, physical contact, grooming, cleaning, basic obedience commands, walking, and agility routes					
**Stefanini et al. (2016)**	The CAT program had four phases: (1) familiarization with the animal and the handler: patients were already familiarized with their animal in two introductory sessions and an appropriate animal-patient pairing was established until the end of treatment; (2) individual intervention: the aim of the individual session was to gradually experiment the ways to interact with the animal; the relations with the animal were given priority; the patient spontaneously engaged in taking care of the animal and becoming familiar with non-verbal language; (3) group activity: in group sessions the objective was to test psychosocial functioning, the activity gave priority to a cooperative relationship with the other members of the group and to team work; (4) discussion of the CAT experience: the team looked at the video recordings for the purpose of compiling the observation forms and discussed the progress of the treatment. During each session the participant interacted with a dog and its handler in the following way: play activities, physical contact, grooming, cleaning, basic obedience commands, walking, and agility routes.	10 sessions (45 minutes) for three months	Both (5 in group, 5 in individual)	Not specified	Staff members trained in dog-assisted therapy programmes	Conducted in the hospital's garden or in an activity room when weather was bad.
**Travers et al. (2015)**	Semi-structured session protocols were developed and implemented. Each session involved an introductory activity, general discussion, opportunity for participant to individually interact with the dog (playing, petting, feeding), and concluded by reading a short story to the group. Only one dog was present at a session, and the dogs were used in a rotating order to minimise stress to dogs	2 x 40-50 minute sessions/ weekly (two consecutive days) for 11 weeks	Group of approx. 10	1 x miniature poodle, 1 x Staffordshire terrier, 1 x German shepherd	Therapist who was also a veterinarian and a registered psychiatric nurse	Private room to conduct the sessions in each facility
**Vidal et al. (2020)**	Sessions in the individual module were as follows: session 1, getting to know each other to determine the strengths and weaknesses of each patient; session 2,	12 sessions approx. 45 minutes	6 individual; 6 group (3 – 4)	Not specified	2 dog-assisted therapist professionals and psychologist	Not specified

	frustration tolerance and motivation; session 3, impulsivity management (self-control strategies, sequential thinking); session 4, emotional self-regulation (identifying emotional triggers and alarm signs); session 5, executive functions (planning, cause effect thinking); session 6, review of the contents. The last six group sessions focused on social skills (criticism management, communication and cooperation, adaptive behaviour, assertiveness training and empathy). Dogs facilitated the achievement of the therapeutic goals set by the psychologist who conducted the intervention.	over 3 months				
**Vidal et al. (2023)**	Sessions in the individual module were as follows: session 1, getting to know each other to determine the strengths and weaknesses of each patient; session 2, frustration tolerance and motivation; session 3, impulsivity management (self-control strategies, sequential thinking); session 4, emotional self-regulation (identifying emotional triggers and alarm signs); session 5, executive functions (planning, cause effect thinking); session 6, review of the contents. The last six group sessions focused on social skills (criticism management, communication and cooperation, adaptive behaviour, assertiveness training and empathy). Dogs facilitated the achievement of the therapeutic goals set by the psychologist who conducted the intervention.	1 x weekly sessions (45 minutes) for 16 weeks	8 individual; 8 group (3-4)	Not specified	One technician specialising in dog-assisted therapy and a psychologist for individual group modules	Not specified
**Villalta-Gil et al. (2009)**	Intervention was based on Integrated Psychological Treatment (IPT). It is a group intervention structured programme with five subprogrammes: cognitive differentiation, social perception, verbal communication, social skills training, and interpersonal problem solving. In this study, it was partially modified to bring dogs into the therapy sessions. Sessions for the dog assisted group were designed so the handler interacted with the dog and the therapist, the therapist interacted with the patient and the handler, and	2 x weekly sessions (45 minutes) for 25 sessions	Small groups (3-4)	Labrador	Trained psychologist	Not specified

	patients interacted with the dog and the therapist. Sessions were also designed so materials used in the IPT (cards, sentences, etc.) were substituted by, or were referenced to, the dog. The dog had an active role within the sessions.					
**Wijker et al. (2020; 2021)**	The programme was developed by therapists and dog behavioural specialists from a Dutch service dog foundation and psychologists from a mental health care organisation who had a specialisation in Autism. The programme had a structured protocol, and a therapy dog was involved during all sessions. During the sessions, the therapists used a semi-structured therapy protocol. The aim of the intervention was to reduce stress and stress-related outcomes, such as depression and anxiety and to improve social and communication skills.	10 weekly sessions (60 minutes)	Individual	2 x Labradors, 4 x Labrador crossbreeds , 1 x golden retriever, 3 x golden retriever crossbreeds , 2 x poodles, 1 German Wirehaired Pointer	Therapists who had completed advanced courses in dog behaviour and welfare.	Therapy room
**Wolynczyk-Gmaj et al. (2021)**	The participant exited the building and was introduced to the dog's handler and the assistance dog, who were already waiting in the surrounding green area. The handler was asked to focus the conversation on the dog and the participant's experience with dogs in general and he could also show the skills of his dog, so the test conditions were comparable for all subjects. Physical contact with the dog (such as petting) was permitted, the participant was free to decide on the level of interaction.	One 15-20 minute session	Individual	German Shepherd	Professional animal handler	Garden outside the ward

Supplementary Material 6. Risk of bias summary: review authors' judgements about each risk of bias item for each included paper.

	Random sequence generation (selection bias)	Allocation concealment (selection bias)	Blinding of participants and personnel (performance bias)	Blinding of outcome assessment (detection bias)	Incomplete outcome data (attrition bias)	Selective reporting (reporting bias)	Other bias
Allen 2022	+	+	−	+	+	+	+
Baek 2020	+	?	−	+	?	?	?
Bono 2015	?	?	−	?	+	?	+
Briones 2021	+	?	−	+	−	?	+
Calvo 2016	?	?	−	−	−	?	+
Chen 2021	+	+	−	−	+	+	+
Chen 2022	+	+	−	−	+	?	+
Chu 2009	?	?	−	+	−	−	+
Friedmann 2015	+	?	−	?	+	?	+
Fung 2014	+	?	−	+	+	?	+
Hill 2020	+	+	−	−	+	+	+
Majic 2013	?	?	−	+	+	?	+
Meints 2022	?	?	−	?	−	?	+
Menna 2019	?	?	−	?	?	?	?
Nieforth 2024	?	?	−	+	+	?	+
Olsen 2016a	+	?	−	−	+	+	+
Olsen 2016b	+	?	−	−	+	+	+
Parra 2021	?	+	−	+	+	?	+
Parra 2022	?	?	−	?	+	?	+
Schuck 2015	?	?	−	−	+	?	?
Schuck 2018a	?	?	−	−	?	?	+
Schuck 2018b	?	?	−	−	+	?	+
Scorzato 2017	+	+	−	+	+	?	+
Shih 2023	+	+	+	−	+	+	+
Stefanini 2015	+	?	−	+	+	?	+
Stefanini 2016	+	?	−	+	?	?	+
Travers 2013	+	?	−	+	+	?	+
Vidal 2020	+	+	−	+	+	+	+
Vidal 2023	+	+	−	+	+	+	+
Villalta Gil 2009	?	?	−	+	?	?	?
Wijker 2020	+	+	−	+	+	+	+
Wijker 2021	+	+	−	+	+	+	+
Wolynczyk Gmaj 2021	?	?	−	−	+	−	+

Supplementary Table 7. Definitions/terminology used by authors to classify DAIs

Author (year)	DAI type	Description of goals	Description of animal handler/animal teams	Description of dogs involved	Category
**Mental health conditions**					
Allen et al. (2022)	Dog-assisted therapy	Involvement of dog to facilitate therapeutic goals associated with trauma	Professionally trained dog handlers observed all sessions but were not directly involved, and trained clinician prior to sessions	Team of trained service dogs via a local service dog organisation that performs breeding, training and placement of service dogs	Clear alignment
Calvo et al. (2016)	Dog-assisted therapy	No goals reported, and authors state these sessions were 'activities' between psychosocial rehabilitation sessions	Delivered by a member of the research team	Study only refers to 'therapy dogs'	Misalignment
Chen et al. (2021; 2022)	Dog-assisted therapy	Content designed to improve negative symptoms and general psychopathology symptoms associated with schizophrenia	Delivered by an animal-assisted therapist, an occupational therapist and a dog-handler pair (breeder).	Therapy dogs (and breeders) had been certified by the Professional Animal-Assisted Therapy Association of Taiwan	Clear alignment
Chu et al. (2009)	Dog-assisted activity	N/A	Delivered by a member of the research team	Trained dogs provided by trainers, but trainers did not accompany dogs during activities	Clear alignment
Shih et al. (2023)	Dog-assisted therapy	Content designed to improve skills in social interaction and emotional expression, with activities with dog designed to meet these goals	Delivered by qualified animal-assisted therapists with experience providing services to people with disabilities	Two service dogs with their handlers	Clear alignment
Stefanini et al. (2015)	Dog-assisted therapy	Structured sessions in accordance with individual therapeutic goals for each participant	Delivered by qualified animal-assisted therapists	Dogs trained for animal-assisted therapy and activities to the standards of Pet-Partner®, ex-Delta Society	Clear alignment
Stefanini et al. (2016)	Dog-assisted therapy	Intervention followed individual objectives for each participant and was	Delivered by qualified animal-assisted therapists	Dogs trained for animal-assisted therapy and activities to the standards of Pet-Partner®, ex-Delta Society	Clear alignment

		integrated into the participant's plan of care			
Wolynczyk-Gmaj et al. (2021)	Dog-assisted activity	N/A	Delivered by dog handler (with no further information)	Dog involved was owned by the dog handler	Clear alignment
			**Neurodevelopmental conditions**		
Fung et al. (2014)	Dog-assisted therapy	Structured, therapist-directed play therapy. Involvement of dog to facilitate therapeutic outcomes	Delivered by qualified therapist with additional training in animal-assisted therapy	Qualified therapy dogs recruited from the Animal Asia Foundation. All dogs had passed an assessment of temperament, obedience skills, and coping with stress.	Clear alignment
Hill et al. (2020)	Dog-assisted therapy	Intervention began with goal setting, and all subsequent sessions consisted of occupation focused, goal-directed content	Delivered by qualified therapist with additional training in animal-assisted therapy	Trained therapy dog who had completed training and assessment as a therapy dog with her handler (qualified therapist delivering sessions)	Clear alignment
Meints et al. (2022; Study 2)	Dog-assisted activity	N/A	Delivered by volunteers who were members of Pets as Therapy	Dogs of volunteers who had passed assessments by independent dog behaviour specialists on their suitability to work with children	Clear alignment
Schuck et al. (2015)	Dog-assisted therapy	Content based on cognitive-behavioural therapies to enhance specific skills for children with ADHD. Involvement of dog aimed to facilitate these therapeutic outcomes	Delivered by qualified animal handlers with their dogs	Certified therapy dogs	Clear alignment
Schuck et al (2018a; 2018b); Nieforth et al. (2024)	Dog-assisted therapy	Content based on cognitive-behavioural therapies to enhance specific skills for children with ADHD. Involvement of dog aimed to facilitate	Delivered by qualified animal handlers with their dogs	Certified therapy dogs	Clear alignment

		these therapeutic outcomes			
Scorzato et al. (2017)	Dog-assisted therapy	Content designed for rehabilitation, with different activities adapted to meet individual participant's needs and abilities	Delivered by a qualified dog handler	Specially trained dogs meeting specific health prerequisites for animal-assisted interventions	Clear alignment
Vidal et al. (2020)	Dog-assisted therapy	Dogs facilitated the achievement of therapeutic goals set by the psychologist who conducted the intervention.	Delivered by a dog-assisted therapist and a psychologist	Certified therapy dogs who were trained and tested to work with people, and their mental and physical health care was strictly monitored	Clear alignment
Vidal et al. (2023)	Dog-assisted therapy	Content was designed to meet specific goals each week (e.g., frustration tolerance, emotional self-regulation, impulsivity management)	Delivered by a qualified dog-assisted therapy technician and a psychologist	Certified therapy dogs	Clear alignment
Villalta-Gil et al. (2009)	Dog-assisted therapy	Content had been developed to work on cognitive and social functioning, and was a structured programme to meet these goals	Delivered by a trained psychologist and qualified animal handler	Certified therapy dog	Clear alignment
Wijker et al. (2020; 2021)	Dog-assisted therapy	Semi-structured therapy protocol was developed by therapists and dog behavioural specialists, with the goals to reduce stress, depression, anxiety, and improve social skills	Delivered by a qualified psychologist who had completed advanced courses on dog behaviour	Trained service dogs, selected and trained by the Dutch Service Dog Foundation	Clear alignment
			**Dementia**		

Baek et al. (2020)	Dog-assisted therapy	Content designed to enhance cognitive function, emotional status, problematic behaviours, and activities of daily living	Team consisted of three qualified animal mediation psychologists and one animal handler	Therapy dog who had completed all health, aptitude and technical tests and had experience of working in therapy sessions	Clear alignment
Bono et al. (2015)	Dog-assisted therapy	Not reported	Study only refers to 'animals and their tutors'	Study only refers to 'animals and their tutors'	Unclear alignment
Briones et al. (2021)	Dog-assisted therapy	Content designed to improve cognitive capacities, motor skills, and enhance social relations	Delivered by a trained animal-assisted therapy technician	Trained dogs with prior experience of working in therapy sessions	Clear alignment
Friedmann et al. (2015)	Dog-assisted activity	N/A	Delivered by a nurse practitioner experienced with working with older adults with dementia	Therapy dogs with experience of visiting care homes	Clear alignment
Majic et al. (2013)	Dog-assisted therapy	Content designed to enhance social interaction and functioning	Delivered by a qualified animal handler	Specially educated therapy dogs, with an exclusive relationship to their handler and a good level of obedience	Clear alignment
Menna et al. (2019)	Dog-assisted therapy	Goals not reported but based on formal reality orientation therapy with a specific protocol	Delivered by a veterinary zootherapist-handler	Study only refers to 'therapy dog'	Unclear alignment
Olsen et al. (2016a)	Dog-assisted activity	N/A	Delivered by a qualified dog handler	Dogs passed screening tests to assess personality traits and behaviour when interacting with humans	Clear alignment
Olsen et al. (2016b)	Dog-assisted activity	N/A	Delivered by a qualified dog handler	Dogs passed screening tests to assess personality traits and behaviour when interacting with humans	Clear alignment
Parra et al. (2021)	Dog-assisted therapy	Content of sessions was developed according to goals, aimed to enhance affective, behavioural, functional and cognitive domains	Delivered by a qualified dog-assisted therapy technician	Dogs were selected according to good health, high sociability, high impulse control and high learning ability. They were trained by their handlers for specific exercises of the intervention	Clear alignment

Parra et al. (2022)	Dog-assisted therapy	Goals set by occupational therapist and the dog-assisted therapy technician based on participants' needs, and content was aligned to meet these goals	Delivered by a qualified dog-assisted therapy technician	Trained dog who had many years of dog-assisted therapy experience ad had passed temperament, health, and fitness for grooming tests	Clear alignment
Travers et al. (2013)	Dog-assisted therapy	Semi-structured session protocols were developed, and dogs were brought into therapy sessions to stimulate discussion	Delivered by a qualified therapist who was a veterinarian and had received training in dog-assisted therapy for people with dementia	Dogs owned by the therapist were involved, and were either Delta Society accredited or accredited by the consultant therapist	Clear alignment
